# Insect glycerol transporters evolved by functional co-option and gene replacement

**DOI:** 10.1038/ncomms8814

**Published:** 2015-07-17

**Authors:** Roderick Nigel Finn, François Chauvigné, Jon Anders Stavang, Xavier Belles, Joan Cerdà

**Affiliations:** 1 Department of Biology, Bergen High Technology Center, University of Bergen, PO Box 7803, N-5020 Bergen, Norway; 2 Institute of Marine Research, PO Box 1870 Nordnes, 5817 Bergen, Norway; 3 Institut de Recerca i Tecnologia Agroalimentàries (IRTA)-Institut de Ciències del Mar, Consejo Superior de Investigaciones Científicas (CSIC), Passeig Marítim 37-49, 08003 Barcelona, Spain; 4 Institute of Evolutionary Biology (CSIC-Universitat Pompeu Fabra), Passeig Marítim 37-49, 08003 Barcelona, Spain

## Abstract

Transmembrane glycerol transport is typically facilitated by aquaglyceroporins in Prokaryota and Eukaryota. In holometabolan insects however, aquaglyceroporins are absent, yet several species possess polyol permeable aquaporins. It thus remains unknown how glycerol transport evolved in the Holometabola. By combining phylogenetic and functional studies, here we show that a more efficient form of glycerol transporter related to the water-selective channel AQP4 specifically evolved and multiplied in the insect lineage, resulting in the replacement of the ancestral branch of aquaglyceroporins in holometabolan insects. To recapitulate this evolutionary process, we generate specific mutants in distantly related insect aquaporins and human AQP4 and show that a single mutation in the selectivity filter converted a water-selective channel into a glycerol transporter at the root of the crown clade of hexapod insects. Integration of phanerozoic climate models suggests that these events were associated with the emergence of complete metamorphosis and the unparalleled radiation of insects.

## Abstract (web summary)

Insects can accumulate high levels of glycerol as an adaptive response to dessication and freezing. Here, the authors show that glycerol transporters evolved from water-selective channels that co-opted the glycerol transport function of ancestral aquaglyceroporins in the oldest lineages of insects.

## Graphical abstract

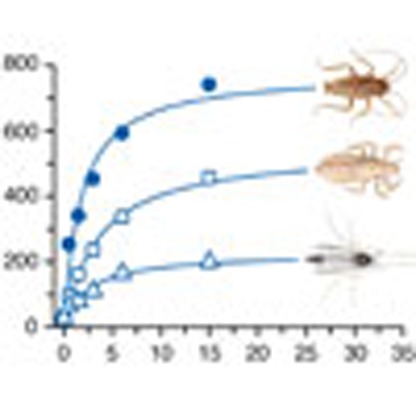

Established models suggest that evolutionary novelty typically arises from gene duplication followed by gradual neofunctionalization or subfunctionalization^1^,^2^. Due to the modular and interrelated nature of multigene families, however, an alternative non-linear pathway of gene evolution could occur if a chance mutation in a duplicated gene lead to the gain-of-function of a distantly related member of the superfamily. The potential for such a rewired evolutionary pathway has been observed in engineered immotile strains of bacteria that can recover their lost flagella in just a few mutational steps^3^. Whether real world examples of such functional co-option and gene replacement have occurred through natural selection remains to be established.

In terms of numbers of species, the holometabolan insects (Holometabola; or superorder Endopterygota) that undergo complete metamorphosis mediated by a unique pupal stage are the most successful clade of organisms in the history of life, with current estimates accounting for more than half of the world's eukaryotic biodiversity^4^. As in other insects, holometabolan species can accumulate high levels of colligative polyols, such as glycerol or sorbitol, as an adaptive response to dessication and freezing temperatures^5^,^6^,^7^. While maximum glycerol accumulation and cold tolerance predominantly occurs in the pupal stage^8^,^9^,^10^, recent phylogenetic studies of insect aquaporins have suggested that members of the aquaporin gene superfamily known as aquaglyceroporin (Glp) channels, which typically transport water and glycerol, are absent in holometabolan insects^11^,^12^,^13^. This notion nevertheless contrasts with experimental studies showing that some aquaporins from different insect species can transport glycerol^14^,^15^,^16^,^17^,^18^,^19^. Such opposing observations represent a paradox since it has been established that the aquaporin superfamily split into two major phyletic branches before the evolution of Eukaryota, one that includes multifunctional Glps and the other water-selective aquaporins^13^,^20^,^21^. The structural basis for the selective aquaporin permeation properties was resolved through crystallographic studies, which determined that conserved central Asn-Pro-Ala (NPA) motifs and a quartet of aromatic arginine (ar/R) residues in the outer channel vestibule form the major selectivity filters^22^,^23^. Experimental studies have also shown that at least two ar/R point mutations are required to convert a typical water-selective channel, such as mammalian aquaporin-1 (AQP1), into a glycerol transporter^24^, yet no such substitutions have been observed in the vertically transferred aquaporin repertoires of extant biota, despite billions of years of evolution^25^. We therefore phylogenetically investigated the interrelationships of insect aquaporins and glycerol transporters in relation to the genomic repertoires of other arthropods and used mutagenesis studies to experimentally probe the origin and functional evolution of the insect glycerol transporters. Our data reveal that mutated water-selective channels specifically co-opted the glycerol transport function of the ancestral Glps in the oldest lineages of hexapod insects. Uniquely, these new, more efficient forms of glycerol transporters were positively selected in the megadiverse holometabolan insects resulting in the replacement of the Glps as the major vehicles of polyol conductance.

## Results and discussion

### Insects genomes encode non-classical glycerol transporters

**Figure 1:**
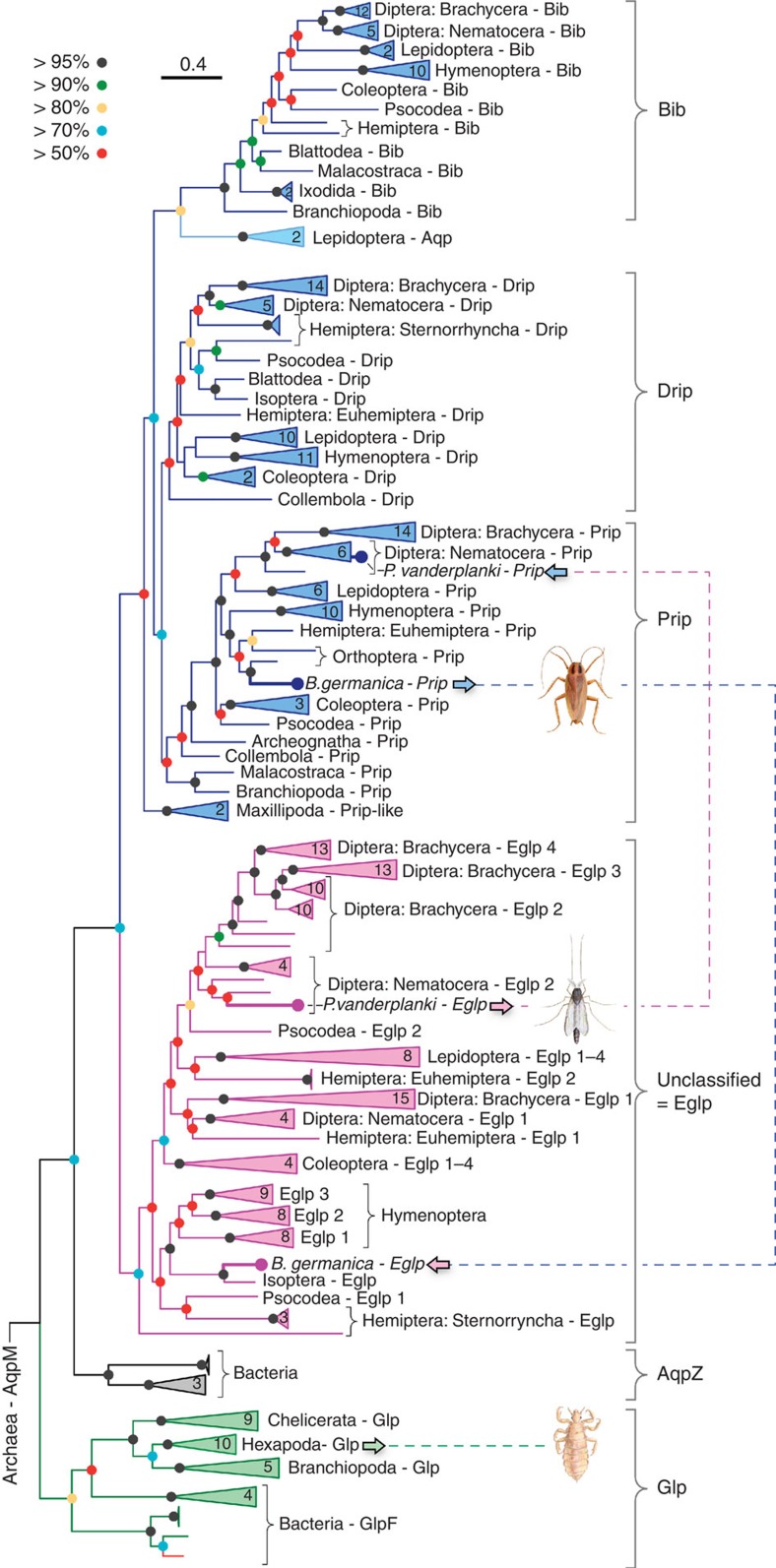
Molecular phylogeny of arthropod orthodox aquaporins. The tree was inferred through maximum likelihood (3,002,760 heuristic rearrangements) and Bayesian analysis (15 million MCMC generations) of 219,583 nucleotide sites in a codon alignment of 269 non-redundant aquaporins, and was rooted with aqpM. The number of taxa in collapsed (triangular) nodes are indicated, with coloured circles at each node indicating posterior probabilites as defined by the key. Scale bar represents the rate of nucleotide substitution per site. Dotted lines and insect images illustrate the conducted experiments.

To resolve the glycerol paradox in holometabolan insects, we conducted an indepth phylogenetic analysis of 269 non-redundant arthropod aquaporins assembled from available whole-genome and transcriptome shotgun sequences. These data show that the arthropod orthodox aquaporin superfamily consists of five major subfamilies, including classical Glps, water and urea-transporting channels termed *Pyrocoelia rufa* integral proteins (Prip)^26^,^27^, water-specific channels termed *Drosophila* intrinsic proteins (Drip)^28^,^29^,^30^,^31^,^32^, the cation permeating big brain channels (Bib)^33^,^34^, and a previously unclassified clade (Fig. 1). A sixth group of unorthodox aquaporins related to the vertebrate Aqp12 channels is also identified in all insects and other protostome animals (Supplementary Table 1). Although we identify *glp* genes in approximately half of the extant orders of insects (see below), BLAST searches using nucleotides or peptides corresponding to the exons as queries did not uncover classical *glps* in 46 of the 47 species of holometabolan insect studied. Only an N-terminal fragment corresponding to the first of seven exons of the human body louse (*Pediculus humanus*) *glp* was detected in the neuropteran Ant lion (*Euroleon nostras*). To support these observations, we conducted an alignview synteny analysis using the Genomicus database (Supplementary Fig. 1), which allows an intuitive visualization of gene loss or gene gain during evolution^35^. These separate analyses based on currently available genome information further suggest the loss of *glps* in holometabolan insects, thus confirming previous reports^11^,^12^,^13^.

**Figure 2:**
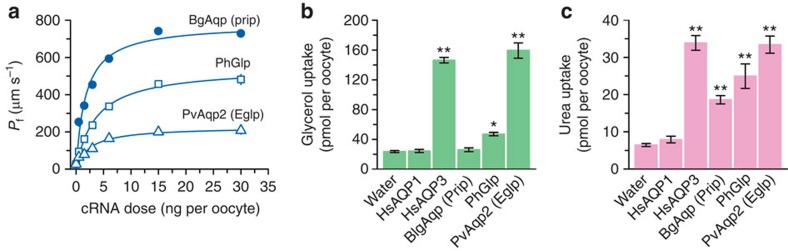
Permeation competence of insect glycerol transporters. (**a**) Osmotic water permeability (*P*_f_) of *X. laevis* oocytes injected with water (controls) or expressing the cockroach water channel (BgAqp), a human body louse aquaglyceroporin (PhGlp) and the sleeping chironomid Eglp (PvAqp2), in relation to the cRNA dose injected of each aquaporin. (**b**) Glycerol and (**c**) urea uptake by oocytes injected with water, or 15 ng of BgAqp, PhGlp or PvAqp2. Human AQP1 and -3 were respectively used as negative and positive controls. Data (mean±s.e.m.) are from three separate experiments (6–10 oocytes per group in each experiment). *P<0.05, **P<0.01 versus water-injected controls (one-way analysis of variance).

Strikingly, the phylogenetic analyses indicate that lepidopteran (moth), hemipteran (true bug) and dipteran (mosquito) aquaporins associated with glycerol transport^14^,^15^,^16^,^17^,^18^,^19^, all resolve within the previously unclassified clade of aquaporins rather than in the classical Glp clade (Fig. 1). Since the previously unclassified clade of aquaporins clusters above bacterial AqpZ as a sister clade to the Prip and Drip subfamilies, each of which are on the water-selective branch of superfamily, the phylogenetic data suggest that non-classical glycerol-transporting aquaporins may have evolved from classical water-selective channels in certain species of insect. However, other hemipteran and dipteran aquaporins, including *Lygus hesperus* LhAqp2 and -3 and *Polypedilum vanderplanki* PvAqp2, respectively, that are reported to be water specific^36^,^37^, also resolve within the unclassified cluster, suggesting that not all members of this cluster are capable of permeating glycerol. Inspection of the ar/R selectivity residues^22^,^23^ nevertheless reveals that, as in the lepidopteran, hemipteran and dipteran channels that do permeate glycerol^14^,^15^,^16^,^19^, PvAqp2 encodes an uncharged residue (Ala174) on transmembrane domain 5 (TMD5) rather than the fully conserved His of the water-selective Prip and Drip channels (Supplementary Table 2). We therefore re-evaluated the permeability properties of PvAqp2 in relation to evolutionary divergent glycerol and water transporters from insects (*P*. *humanus* PhGlp; *Blattella germanica* BgAqp) and mammals (human AQP1, AQP3) using a heterologous *Xenopus laevis* oocyte expression system. For comparative purposes we first established that 15-ng injected cRNA is the dose at which maximal water permeability is obtained in the PvAqp2, PhGlp and BgAqp channels (Fig. 2a). Subsequent experiments using this cRNA injection dose confirm the water and urea selectivity of the Prip channel (BgAqp^27^), but also demonstrate that in contrast to a previous report^36^, PvAqp2 indeed transports glycerol and urea as efficiently as human AQP3, and more effectively than the classical insect Glp, PhGlp (Fig. 2b,c). To reaffirm the origin of this new form of glycerol transporter, we used Bayesian inference to compare 77 related proteins in 20 basal insect orders to 117 Prip-like proteins in the four extant lineages of arthropods, Chelicerata (scorpions, spiders and ticks), Myriapoda (centipedes), Crustacea (copepods and water fleas) and Hexapoda (insects). The results provide robust statistical evidence that the neofunctionalized glycerol-transporting genes are a unique innovation found only in hexapods (Supplementary Fig. 2), and we therefore term this insect-specific group of aquaporins ‘Entomoglyceroporins' (Eglp).

### Entomoglyceroporins evolved in basal hexapods

Interestingly, unlike the cluster pattern of the single Prip channels in palaeopteran and polyneopteran insects, which reflect the phylogenetic rank of the species investigated, multiple Prip-like paralogs exist in the oldest hexapod lineages (Entognatha and Archeognatha) (Supplementary Fig. 2). The products of these latter gene duplicates either cluster within the Prip subfamily (Diplura and Collembola Ile and Val paralogs) or as an intermediary group between the Prip subfamily and the Eglp cluster in Protura (Ile and Ser paralogs). Conversely, in Archeognatha, three separate clusters are seen as Prip, midway between the Prip and Eglp clusters, and a basal member of the Eglp cluster (Supplementary Fig. 2). To test whether the glycerol-transporting function evolved in the oldest hexapod lineages, we synthesized full-length transcripts of the duplicated collembolan Prip-like and proturan Eglp-like channels that respectively harbour a Val or Ser in the TMD5 selectivity filter and examined their solute uptake properties in oocytes in relation to positive (human AQP3) and negative (human AQP1) controls using 15 ng of injected cRNA. The results show that both of the basal hexapod Prip-like and Eglp-like channels are functional water and glycerol transporters (Supplementary Fig. 3). On the basis of the published values for the divergence times of Hexapoda^38^, the above findings indicate that the Eglp group of genes most likely evolved from duplicated Prip-like water channels at the Ordovician dawn of Hexapoda, but did not comprise an independent subfamily until the early Silurian rise of true insects (Insecta). Conversely, Bayesian inference of panarthropod Glps, supports the existence of multiple gene copies in Tardigrada^39^, Chelicerata, Crustacea and the basal hexapod lineages (Supplementary Fig. 4), while single Glps are encoded in the genomes of roughly half of the extant insect orders (Supplementary Fig. 5). These data thus reveal that classical Glps co-existed with neofunctionalized Eglps in the older insect lineages, but were replaced by Eglps in the megadiverse Holometabola.

### Evolution by functional co-option

**Figure 3:**
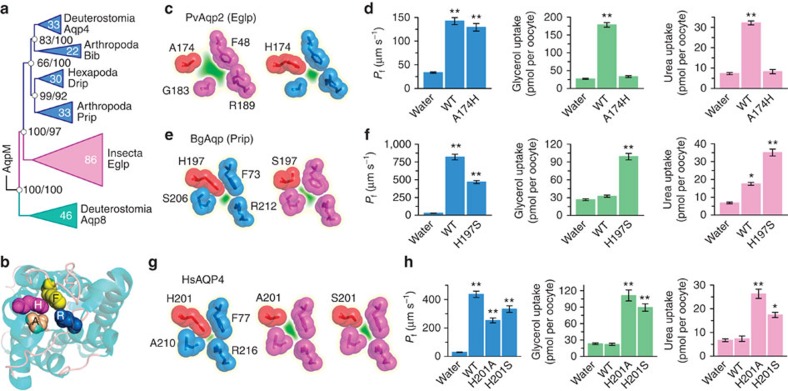
Effect of a single mutation in the ar/R selectivity filter of AQP4-related aquaporins on glycerol and urea permeability. (**a**) Bayesian inference (5 million MCMC generations) of 202,071 nucleotide sites and 65,196 amino acid sites of 247 non-redundant hexapod and deuterostome aquaporins. The tree is rooted with aqpM. Posterior probabilities resulting from analyses of the codon/amino acid alignments are shown at each node, with the scale bar indicating the rate of substitutions per site. (**b**) Extracellular view (cartoon render) of HsAQP4 (3GD8) illustrating the ar/R selectivity filter (spacefill). (**c**, **e** and **g**) The ar/R models of wild-type (WT) PvAqp2 (Eglp) and PvAqp2-A174H mutant (**c**), BgAqp-WT and BgAqp-H197S (**e**), and HsAQP4-WT, HsAQP4-H201A and HsAQP4-H201S (**g**), illustrating the effects of the mutations on the narrowing of the channel pore *in silico*. (**d**, **f** and **h**) Osmotic water permeability (*P*_f_) and solute uptake of *Xenopus laevis* oocytes expressing the WT and mutant aquaporins indicated in **c**, **e** and **g**. Data (mean±s.e.m.) are from three separate experiments (6–10 oocytes per group in each experiment). *P<0.05, **P<0.01 versus water-injected controls (one-way analysis of variance).

A second question concerning the origin of the Eglp subfamily relates to its deeper history from ancestral grades of aquaporin. Since the metazoan aquaporin superfamily includes both Aqp8 and Aqp4 grades^21^, we expected the Eglp subfamily to be a derivative of Aqp8, because some vertebrate Aqp8 orthologs have been shown to transport glycerol^40^. However, Bayesian inference of 247 non-redundant hexapod and deuterostome aquaporins provide robust statistical support (>97% posterior probability) that insect Eglp channels are orthologs of Aqp4 (Fig. 3a). A more extensive analysis of 713 non-redundant prokaryotic, protist, fungal and metazoan aquaporins confirms the Aqp4 orthology of the Eglp channels (Supplementary Fig. 6). This surprising finding not only suggests that arthropod genomes have lost Aqp8-related orthologs, but that the Eglp subfamily specifically evolved from a branch of water-selective channels that sterically exclude the passage of glycerol due to the His residue that lies directly in the ar/R selectivity filter on TMD5 (Fig. 3b, ref. 41). Interestingly, in each of the 207 Eglp channels examined, the TMD5 His residue is substituted for uncharged amino acids, with the majority (73%) of substitutions incorporating Ala or Ser (Supplementary Table 2).

**Figure 4:**
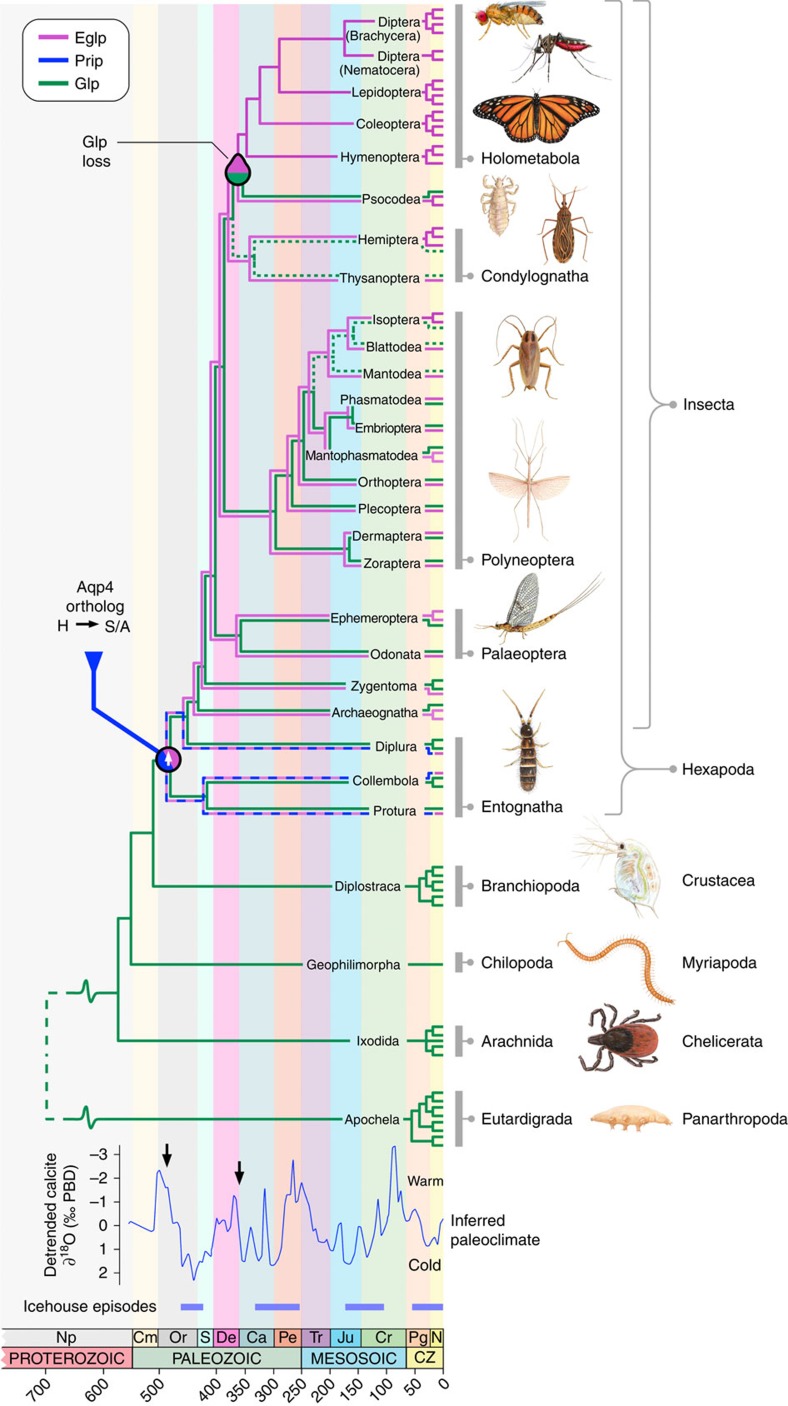
Emergence and extinction of insect glycerol transporters. Schematic overview of aquaglyceroporin (Glp) and entomoglyceroporin (Eglp) evolution in relation to the divergence times of Arthropoda, with multiple terminal branches representing the genomic copy number. Entwined blue and magenta lines indicate incomplete lineage separation of Prip and Eglp genes, while green dotted lines indicate putative loss of Glps. Arthropod divergence times are based on molecular estimates^38^,^53^, with dashed lines indicating uncertain age. Two saltatory events (vertical arrows on inferred paleoclimate) are highlighted in the early Ordovician and Carboniferous periods, when Elgps respectively evolved from Aqp4-related Prip orthologs and were subsequently positively selected in the last common ancestor of the Holometabola. The inferred paleoclimate and icehouse episodes are redrawn from 5/10 detrended running means of δ^18^O calcitic shells^44^. Np, neoproterozoic; Cm, Cambrian; Or, Ordovician; S, Silurian; De, Devonian; Ca, Carboniferous; Pe, Permian; Tr, Triassic; Ju, Jurassic; Cr, Cretaceous; Pg, Paleogene; N, Neogene. Values in the abcissa are millions of years before present.

The above observations prompted us to experimentally probe the molecular evolutionary basis for the functional transformation of a Prip to an Eglp-type channel. To verify that A174 is critical for PvAqp2 to function as an Eglp, we generated a PvAqp2-A174H mutant to mimic the structural constraints of the ancestral Prip channel (Fig. 3c). Expression of the cRNAs in *X. laevis* oocytes shows that the glycerol- and urea-transporting function of the wild type is abolished in the A174H mutant (Fig. 3d). We then selected the Prip of a distantly related blattodean cockroach (*B. germanica*, last common ancestor >380 Ma, ref. 38), which transports water and is also slightly permeable to urea, but not glycerol^27^. The phylogenetic analyses show that *B. germanica* encodes both the Prip and an Eglp channel in which the TMD5 ar/R residues are His and Ser, respectively (Fig. 3e). We therefore generated a BgAqp-H197S mutant and compared the transport kinetics with the wild type in *X. laevis* oocytes. The results confirm the water and urea-conducting properties of the wild-type BgAqp, but also demonstrate that mutating His197 to a Ser likely opens the pore sufficiently to allow the passage of glycerol (Fig. 3f). These findings indicate that all Eglp-type proteins displaying an Ala, Val or Ser residue in the TMD5 ar/R selectivity filter evolved the glycerol transport function. Rather than test each of the hexapod lineages that display this trait, we chose to investigate the most distantly related channel in the phylogeny, human AQP4, with a divergence time from arthropods deep in the Precambrian^42^. Human AQP4 is a water-selective channel displaying the conserved His on TMD5, which reduces the pore to ∼1.5 Å (ref. 41). We generated two AQP4 mutants (H201A and H201S) to infer the functional convergence of the Prip to Eglp-type channel in the majority of hexapods (Fig. 3g), and expressed each in oocytes. The results confirm the water selectivity of wild-type AQP4, and show that both the AQP4-H201A and AQP4-H201S mutants facilitate glycerol and urea transport (Fig. 3h). These data thus experimentally demonstrate that a single mutation in the ar/R selectivity filter is sufficient to convert an integral membrane channel from the water-selective branch of the aquaporin superfamily into a glycerol transporter, a striking innovation that coincided with the emergence of Hexapoda (Fig. 4).

### Glp replacement and Eglp expansion in holometabolan insects

The importance of Eglps for the Holometabola is reflected not only in the fact that they replaced prototypical Glps as the major vehicles of glycerol transport, but in their repeated tandem duplications in the genomes (Fig. 4). Greatest expansion of the *eglp* gene clusters specifically occurred in the holometabolan species that accumulate high levels of polyols within the drought-resistant and cold-hardy pupal stage of the life cycle^8^,^9^,^10^. Consequently, our data suggest that the improved glycerol transport capacity of Eglps over ancestral Glps was an intrinsic molecular trait on which darwinian selection acted. However, since the origin of the holometabolan insects can be traced to the early Carboniferous^38^, with major radiations occuring at the end of the Carboniferous and during the Permian Periods^43^, it seems likely that other extrinsic selective forces were also at play. Phanerozoic climate models show that these periods coincide with fluctuating global temperatures leading to icehouse episodes (Fig. 4)^44^. It thus seems plausible that the positive selection of neofunctionalized water channels that facilitate the efficient accumulation of colligative polyols as a response to drought and freezing conditions was a key adaptation associated with the emergence of holometaboly and the unprecedented radiation of the megadiverse insect groups.

## Methods

### Phylogenetic and *in silico* analyses

Data sets that included bacterial, protist, fungal and metazoan aquaporins were initally assembled from public databases (Ensembl, GenBank). Subsequently aquaporin orthologs were sourced from whole-genome shotgun, transcriptome shotgun and expressed sequence tag databases (NCBI) via tblastn using exon-deduced peptides as queries. Contiguous nucleotide sequences were then retrieved from the respective DNA contigs and trimmed to match each peptide fragment, and subsequently concatenated to construct a putative cDNA for each gene. Combined with the recently assembled deuterostome aquaporin data set a total of 1,560 aquaporins were assembled from non-deuterstome organisms, including the complete superfamilies from 132 panarthropod genomes. Data sets of deduced amino acids were aligned with default t-coffee v9.01 (ref. 45) or L-INS-I MAFFT v7.058b (ref. 46) algorithms, and subsequently converted to codon alignments using Pal2Nal (ref. 47) before Bayesian (Mr Bayes v3.2.2; ref. 48) and maximum likelihood (PAUP v4b10-x86-macosx) methods as described previously^21^,^49^. To detect errors generated by the automated algorithms, alignments were sorted according to the resulting trees and inconsistencies corrected manually using MacVector (MacVector Inc, Cambridge, UK). Phylogenetic analyses of the metazoan superfamily and the arthropod superfamily were performed on the conserved transmembrane regions between Thr31–Phe258 (human AQP4), and Thr61–Ser279 (*Drosophila melanogaster* Bib), respectively, following removal of the N and C termini, while all other analyses were performed on full-length sequences. Bayesian model parameters were nucmodel=4by4, nst=2, rates=gamma for codon alignments and aamodel=mixed for amino acid alignments. Markov chain Monte Carlo (MCMC) algorithms were run with three heated and one cold chain with resulting probability distributions examined for convergence using Tracer version 1.6 (tree.bio.ed.ac.uk/software/tracer/), and majority rule consensus trees summarized with a burnin of 25%. All trees generated were processed with Archaeopteryx^50^ and rendered with Geneious (Biomatters Ltd, New Zealand). A full list of accession numbers is provided in Supplementary Table 2.

The three-dimensional structure of human AQP4 (3GD8) was obtained from the protein data bank (rcsb.org), and *in silico* models of insect and mutant aquaporins built using the model leverage option in the Modeller server (modbase.compbio.ucsf.edu), based on the human AQP4 template. The best scoring models were selected using the slow (Seq-Prf, PSI-BLAST) assignment method and rendered with MacPymol (pymol.org). Synteny analyses were conducted using the Genomicus v25.01 database^35^.

### Aquaporin cDNAs and site-directed mutagenesis

Full-length BgAQP^27^, and synthesized (Life Technologies) PvAqp2, PhGlp, TbPrip-like and AsEglp-like channels following GenBank accession numbers AB281620, XP_002430403, GAXI01015403 and GAXE01015084, respectively, were subcloned into the pT7Ts expression vector. The human AQP1, -3 and -4 cDNAs were kindly provided by Prof. P.M.T. Deen (Radboud University Nijmegen Medical Centre, The Netherlands) and also cloned into pT7Ts. The Quickchange site-directed mutagenesis kit (Agilent Technologies) was used to introduce single nucleotide substitutions in PvAqp2, BgAqp and human AQP4 in pT7Ts. Selected clones were sequenced by BigDye Terminator v3.1 cycle sequencing on ABI PRISM 377 DNA analyzer (Applied Biosystems) to confirm that only the desired mutations were produced.

### Functional expression in *X. laevis* oocytes

The cRNAs for microinjection were synthesized with T7 RNA polymerase (Roche) from XbaI or SalI-linearized pT7Ts-aquaporin (depending on the restriction sites identified in the aquaporin sequence). Isolation of *X. laevis* stages V and VI oocytes and microinjection was performed as previously described^51^. Procedures relating to the care and use of *X. laevis* were approved by the Ethics Committee from IRTA in accordance with the Guiding Principles for the Care and Use of Laboratory Animals. Oocytes were transferred to modified Barth's medium (MBS) containing 88 mM NaCl, 1 mM KCl, 2.4 mM NaHCO_3_, 0.82 mM MgSO_4_, 0.33 mM Ca(NO_3_)_2_, 0.41 mM CaCl_2_, 10 mM HEPES and 25 μg ml^−1^ gentamycin, pH 7.5, and injected with 50 nl of distilled water (negative control) or 50 nl of water solution containing 0.5, 1.5, 3, 6, 15 or 30-ng cRNA. One day after injection, oocytes were manually defolliculated and subsequently maintained in MBS at 18 °C.

For the determination of the osmotic water permeability (*P*_f_), 2 days after injection the oocytes were transferred from isotonic MBS (200 mOsm) to 10-fold diluted MBS (20 mOsm). Oocyte swelling was followed by video microscopy using serial images at 2 s intervals during the first 20 s period using a Nikon Color view video camera coupled to a stereomicroscope (SMZ1000, Nikon). The *P*_f_ values were calculated taking into account the time-course changes in relative oocyte volume [d(*V*/*V*_0_)/d*t*], the partial molar volume of water (*V*_W_=18 cm^3^ mol^−1^) and the oocyte surface area (*S*) using the formula *V*_0_[d(*V*/*V*_0_)/d*t*]/[SVW(Osm_in_−Osm_out_)]. The surface area of the oocyte was considered to be nine times the apparent area because of membrane folding^52^.

Glycerol and urea uptake by *X. laevis* oocytes expressing aquaporins were determined under isotonic conditions. Groups of 10 oocytes injected with water or 15-ng aquaporin cRNA were incubated at room temperature in 200 μl of isotonic MBS containing 5 μM (20 μCi) of [1,2,3-3H]glycerol (50 Ci mmol^−1^) or [^14^C]urea (58 mCi mmol^−1^) (American Radiolabelled Chemicals Inc.) and cold glycerol or urea at 1 mM final concentration. After 10-min exposure to radioactive compounds (including zero time for subtraction of the signal from externally bound solute), oocytes were washed rapidly in ice-cold MBS three times, and individual oocytes were dissolved for 1 h in 400 μl of 10% SDS before scintillation counting

### Statistics

Functional data (mean±s.e.m.) from oocytes were derived from three independent experiments using three different batches of oocytes (6–10 oocytes per treatment). Data were statistically analysed by one-way analysis of variance; *P* values <0.05 were considered significant.

## Additional information

**How to cite this article:** Finn, R. N. *et al*. Insect glycerol transporters evolved by functional co-option and gene replacement. *Nat. Commun.* 6:7814 doi: 10.1038/ncomms8814 (2015).

## Supplementary Material

Supplementary Information Supplementary Figures 1-6 and Supplementary Tables 1-2
