# Supplementary material for: Insect glycerol transporters evolved by functional co-option and gene replacement
Source: Nat Commun. 2015 Jul 17;6:7814. doi: 10.1038/ncomms8814 (PMC4518291; doi:10.1038/ncomms8814)
Supplement: Supplementary Information — Supplementary Figures 1-6 and Supplementary Tables 1-2 [file ncomms8814-s1.pdf]

Supplementary Information

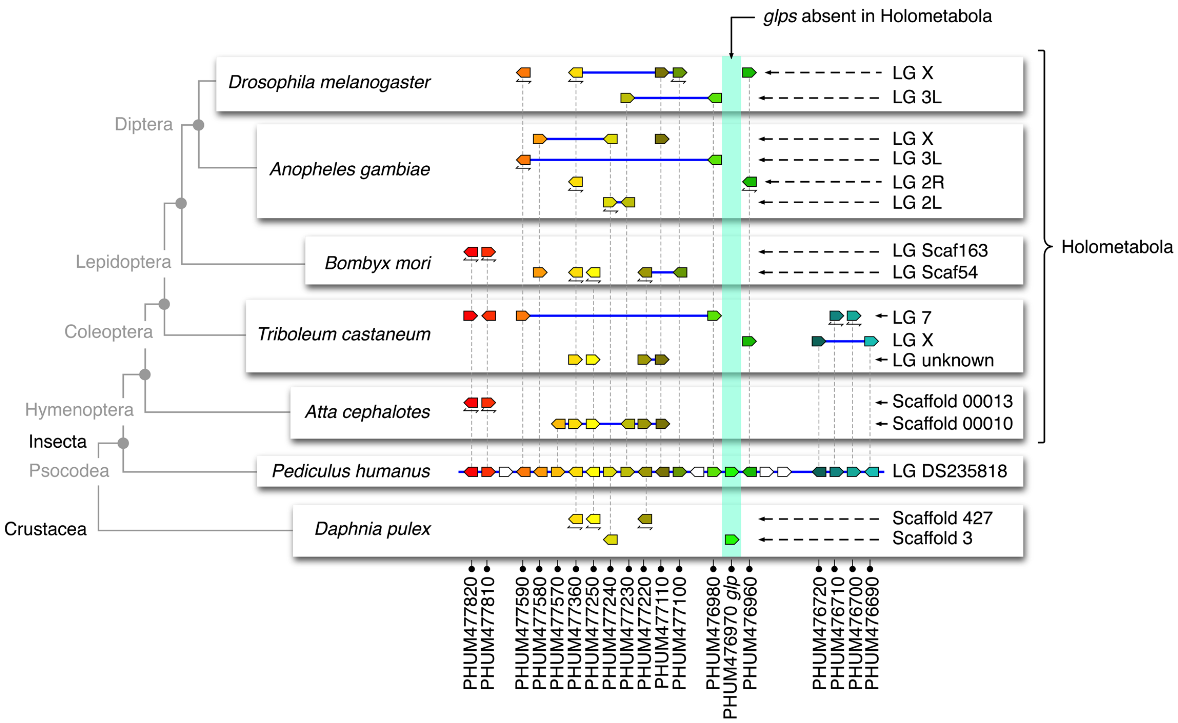

Supplementary Figure 1. Synteny analysis of insect aquaglyceroporins. Data are assembled using the alignview of the Genomicus v25.01 database with human body louse (*Pediculus humanus*) *glp* as the reference gene. White gene symbols represent genes that are not syntenic.

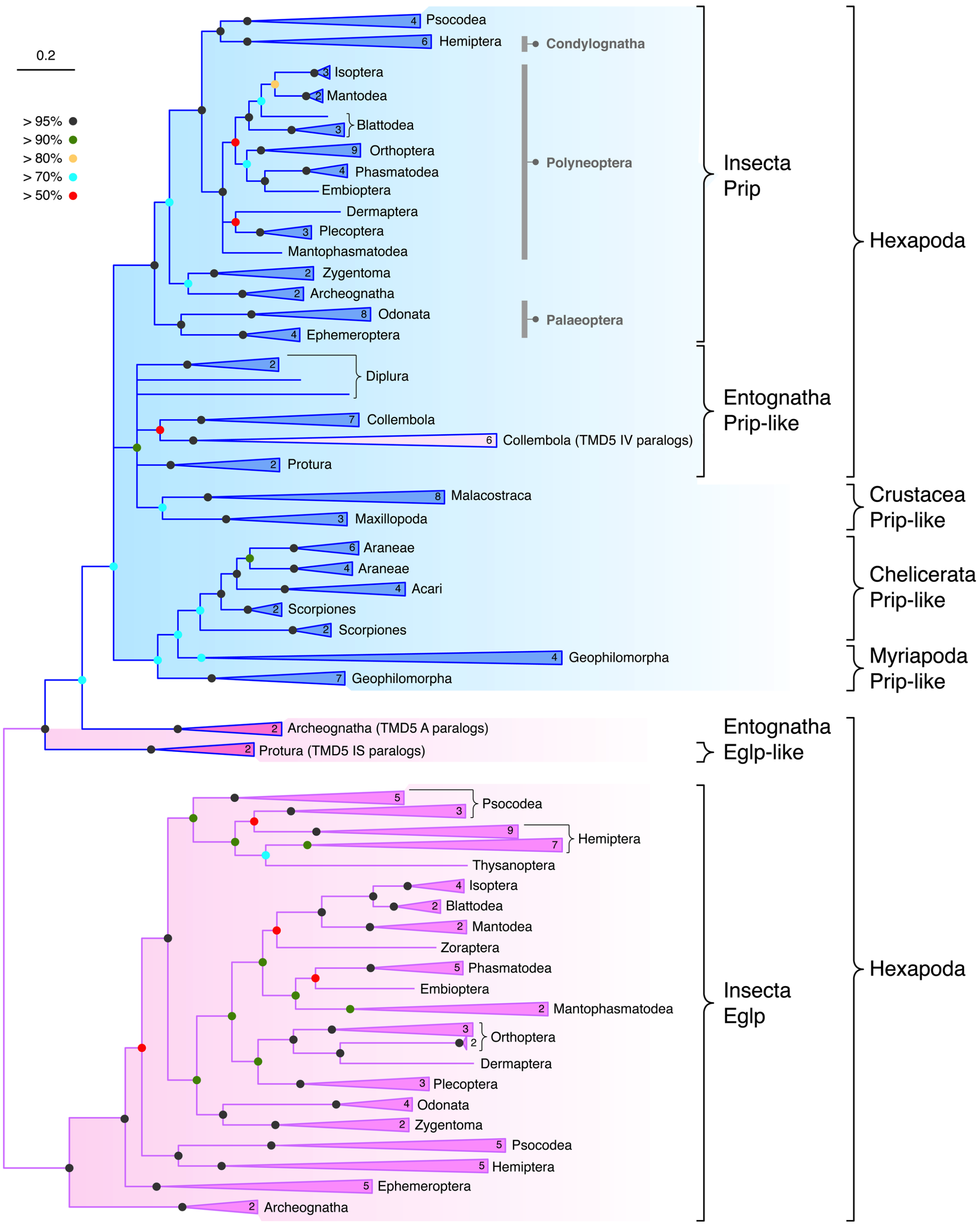

Supplementary Figure 2. Molecular phylogeny of hexapod water and glycerol transporters. Midpoint rooted Bayesian majority rule consensus tree of 194 non-redundant arthropod Prip- and Eglp-like aquaporins inferred from 25 million MCMC generations of 71,605 amino acid sites. The number of taxa in collapsed (triangular) nodes are indicated, with colored circles at each node indicating posterior probabilites as defined by the key. Scale bar represents the rate of amino acid substitution per site. Taxa that do not cluster as Elps, but in which the ar/R His on TMD5 His is naturally mutated are indicated.

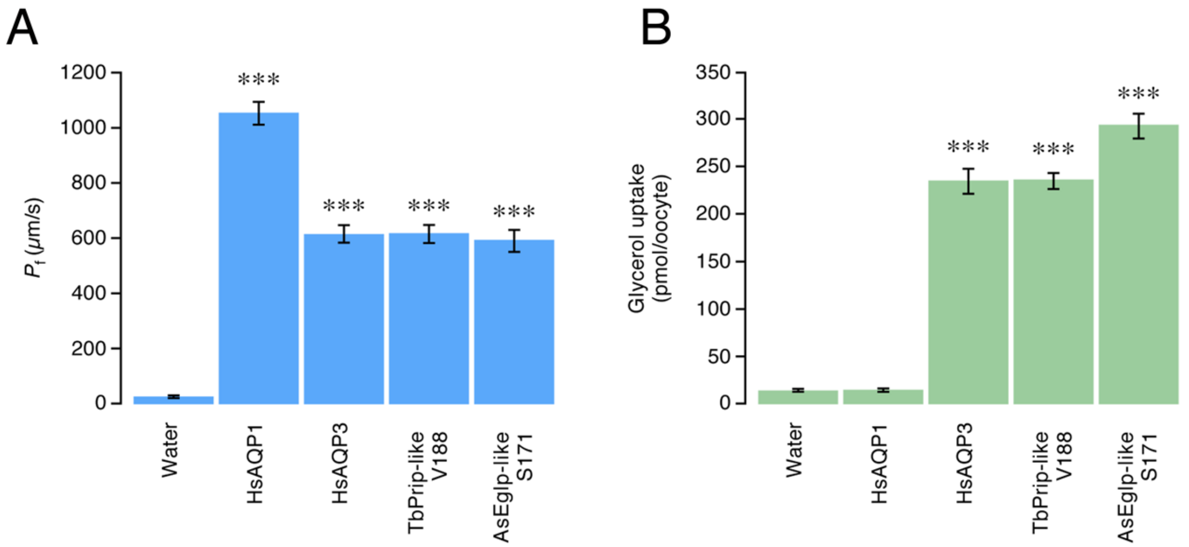

Supplementary Figure 3. Permeation competence of basal hexapod glycerol transporters. (A) Osmotic water permeability (P_f) of *X. laevis* oocytes injected with water (controls), human AQP1 and -3 or 15 ng of the giant springtail (*Tetrodontophora bielanensis*, Collembola) TbPrip-like V188 channel and the conehead (*Acerentomon* sp., Protura) AsEglp-like S171 channel. (B) Glycerol uptake by oocytes injected with water, or 15 ng of the TbPrip-like V188 and AsEglp-like S171 channels. Human AQP1 and -3 were respectively used as negative and positive controls. Data are the mean ± SEM (n = 20 oocytes from two different batches of oocytes). *** P < 0.001 vs water-injected controls (one-way ANOVA).

Supplementary Information

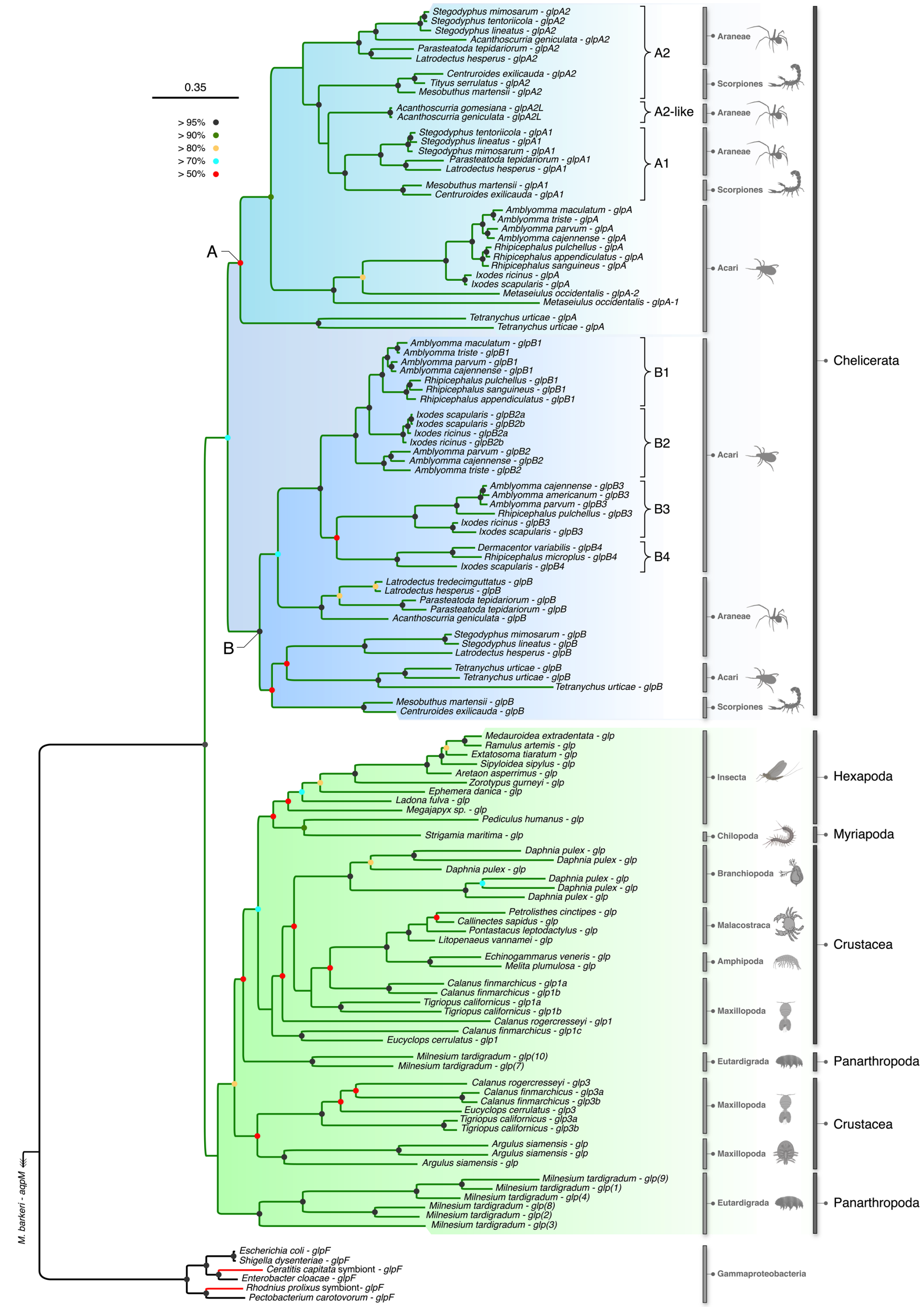

Supplementary Figure 4. Molecular phylogeny of arthropod aquaglyceroporins. AqpM rooted Bayesian majority rule consensus tree of 123 non-redundant arthropod aquaglyceroporins (Glp) inferred from 2 million MCMC generations of 109,126 nucleotide sites. Colored circles at each node indicate posterior probabilites as defined by the key. Scale bar represents the rate of nucleotide substitution per site. The data show that two major clades of Glp (A and B) evolved in Chelicerata, which subsequently underwent differential expansions in scorpions (Scorpiones) and spiders (Araneae) (GlpA1-2) and ticks (Acari) (GlpB1-4). Conversely serial duplications of Glps in water bears (Eutardigrada) and crustaceans (Copepoda and Branchiopoda) occurred independently. Aquaporins putatively derived from prokaryotic symbionts are indicated with red branches.

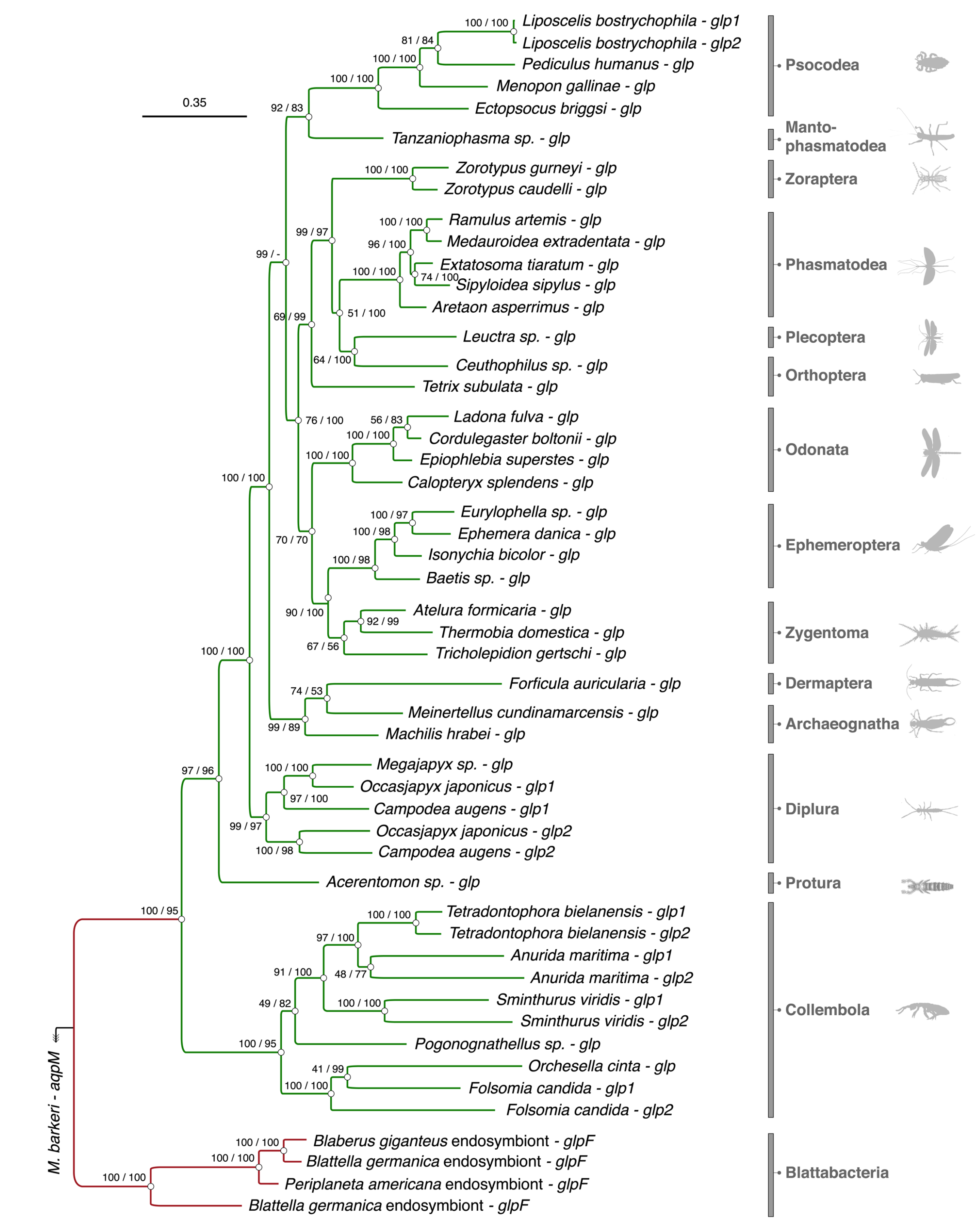

Supplementary Figure 5. Molecular phylogeny of insect aquaglyceroporins. Midpoint rooted Bayesian majority rule consensus tree of 51 non-redundant hexapod aquaglyceroporins (Glp) inferred from 1 million MCMC generations of 65,131 nucleotide sites. Posterior probabilities resulting from the codon/amino acid analyses are shown at each node. Scale bar represents the rate of nucleotide substitution per site. The data show that Glp duplication in Collembola, Diplura and Psocodea is lineage specific. Aquaporins derived from symbiotic Blattabacteria are indicated with red branches.

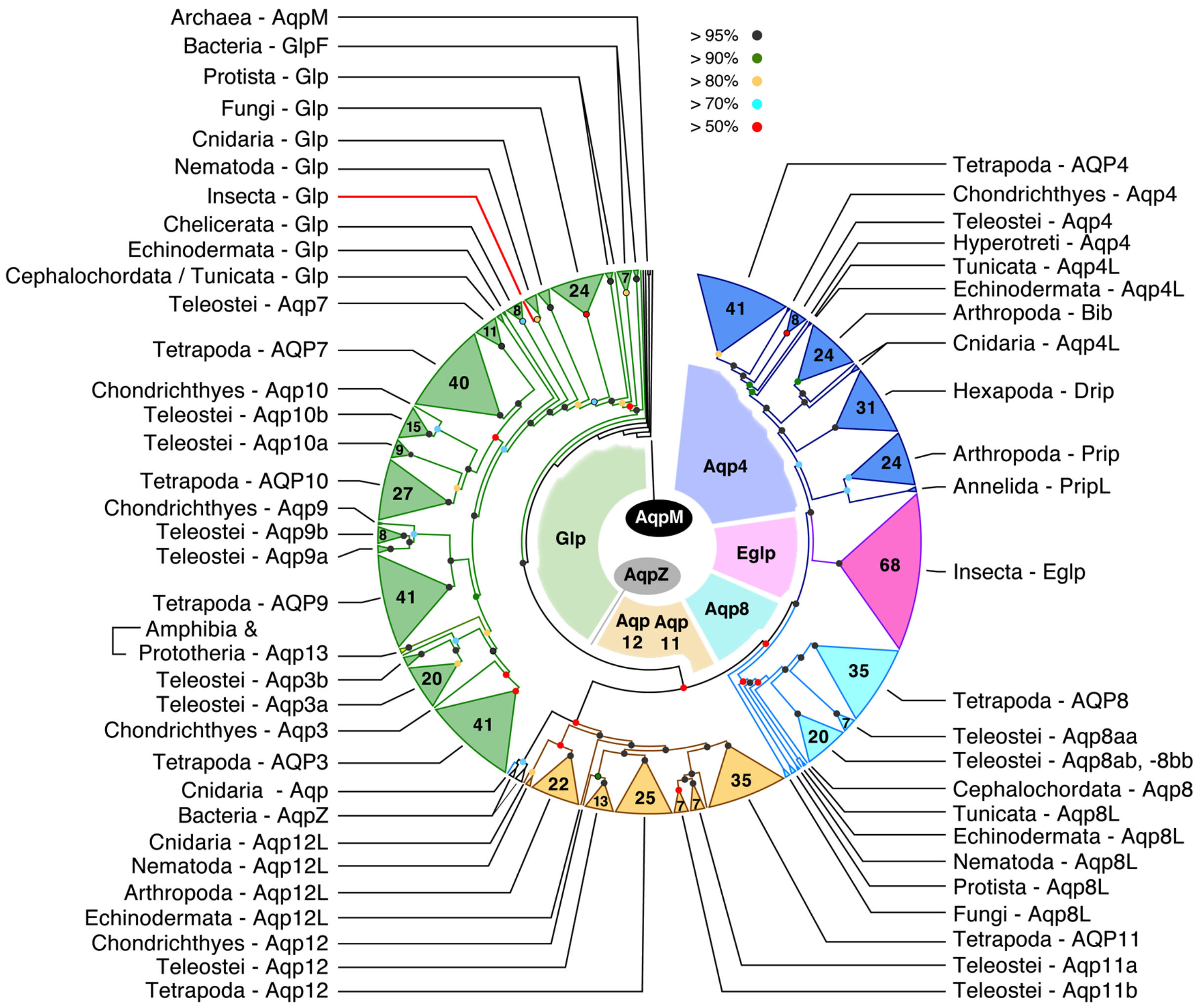

Supplementary Figure 6. Molecular phylogeny of metazoan, fungal, protist and bacterial aquaporins. The tree was inferred through Bayesian analysis (30 million Markov Chain Monte Carlo generations) of 719,229 nucleotide sites in a codon alignment of 713 non-redundant aquaporins, and was rooted with aqpM. Four major grades of aquaporin are annotated: aquaglyceroporins (Glp), unorthodox aquaporins (Aqp11, -12), aquaammoniaporins (Aqp8), and classical aquaporins (Aqp4, including Entomoglyceroporins, Eglp). The ancestral insect Glp is annotated with a red line. Colored circles at each node indicate posterior probabilites as defined by the key.

Supplementary Table 1: Accession numbers of sequences analysed in the study.

Accession #	ortholog	Animal	Species	Rank	Order	Family
**Deuterostomia Aqp4 orthologs**						
ENSP00000372654	AQP 4	Human	*Homo sapiens*	Euarchontoglires	Primates	Hominidae
ENSPTRP00000016898	AQP 4	Chimpanzee	*Pan troglodytes*	Euarchontoglires	Primates	Hominidae
ENSGGOP00000010219	AQP 4	Western lowland gorilla	*Gorilla gorilla gorilla*	Euarchontoglires	Primates	Hominidae
ENSPPYP00000010193	AQP 4	Sumatran orangutan	*Pongo abelii*	Euarchontoglires	Primates	Hominidae
ENSMMUP00000019590	AQP 4	Rhesus macaque	*Macaca mulatta*	Euarchontoglires	Primates	Cercopithecidae
ENSTSYP00000001315	AQP 4	Philippine tarsier	*Tarsius syrichta*	Euarchontoglires	Primates	Tarsiidae
ENSMICP00000002129	AQP 4	Gray mouse lemur	*Microcebus murinus*	Euarchontoglires	Primates	Cheirogaleidae
XP_003784818/ENSOGAP00000003480	AQP 4	Small-eared galago/Bushbaby	*Otolemur garnettii*	Euarchontoglires	Primates	Galagidae
ENSTBEP00000006848	AQP 4	Northern tree shrew	*Tupaia belangeri*	Euarchontoglires	Scandentia	Tupaiidae
ENSMUSP00000078088	AQP 4	Mouse	*Mus musculus*	Euarchontoglires	Rodentia	Muridae
ENSRNOP00000021962	AQP 4	Norway rat	*Rattus norvegicus*	Euarchontoglires	Rodentia	Muridae
ENSDORP00000007647	AQP 4	Ord's kangaroo rat	*Dipodomys ordii*	Euarchontoglires	Rodentia	Heteromyidae
ENSCPOP00000004012	AQP 4	Domestic guinea pig	*Cavia porcellus*	Euarchontoglires	Rodentia	Caviidae
ENSSTOP00000011934	AQP 4	Thirteen-lined ground squirrel	*Ictidomys tridecemlineatus*	Euarchontoglires	Rodentia	Sciuridae
ENSOPRP00000008537	AQP 4	American pika	*Ochotona princeps*	Euarchontoglires	Lagomorpha	Ochotonidae
ENSOCUP00000007625	AQP 4	Rabbit	*Oryctolagus cuniculus*	Euarchontoglires	Lagomorpha	Leporidae
AMDU01061559	AQP 4	Western European hedgehog	*Erinaceus europaeus*	Laurasiatheria	Insectivora	Erinaceinae
ENSSARP00000010861	AQP 4	European shrew	*Sorex araneus*	Laurasiatheria	Insectivora	Soricidae
ENSPVAP00000006319	AQP 4	Large flying fox/Megabat	*Pteropus vampyrus*	Laurasiatheria	Chiroptera	Pteropodidae
ENSMLUP00000010006	AQP 4	Little brown bat/Microbat	*Myotis lucifugus*	Laurasiatheria	Chiroptera	Vespertilionidae
NP_001009279	AQP 4	Sheep	*Ovis aries*	Laurasiatheria	Ruminantia	Bovidae
ENSBTAP00000025341	AQP 4	Cow	*Bos taurus*	Laurasiatheria	Ruminantia	Bovidae
ENSTTRP00000011193	AQP 4	Bottlenosed dolphin	*Tursiops truncatus*	Laurasiatheria	Cetacea	Delphinidae
ENSSSCP00000004028	AQP 4	Pig	*Sus scrofa*	Laurasiatheria	Suina	Suidae
ENSVPAP00000002491	AQP 4	Alpaca	*Vicugna pacos*	Laurasiatheria	Tylopoda	Camelidae
ENSECAP00000003378	AQP 4	Horse	*Equus caballus*	Laurasiatheria	Perissodactyla	Equidae
ENSAMEP00000014983	AQP 4	Giant panda	*Ailuropoda melanoleuca*	Laurasiatheria	Carnivora	Ursidae
ENSCAFP00000026782	AQP 4	Dog	*Canis lupus familiaris*	Laurasiatheria	Carnivora	Canidae
ENSLAFP00000005168	AQP 4	African savanna elephant	*Loxodonta africana*	Afrotheria	Proboscidea	Elephantidae
ENSPCAP00000003928	AQP 4	Cape rock hyrax	*Procavia capensis*	Afrotheria	Hyracoidea	Procaviidae
ENSETEP00000005713/AAIY02093242	AQP 4	Lesser hedgehog tenrec	*Echinops telfairi*	Afrotheria	Afrosoricida	Tenrecidae
ENSDNOP00000010511	AQP 4	Nine-banded armadillo	*Dasypus novemcinctus*	Xenarthra	Cingulata	Dasypodidae
ENSMODP00000026878	AQP 4	Gray short-tailed opossum	*Monodelphis domestica*	Metatheria	Didelphimorphia	Didelphidae
ENSOANP00000014435	AQP 4	Platypus	*Ornithorhynchus anatinus*	Prototheria	Monotremata	Ornithorhynchidae
ENSTGUP00000010943	AQP 4	Zebra finch	*Taeniopygia guttata*	Aves	Passeriformes	Estrildidae
ENSAPLP00000003539	AQP 4	Mallard	*Anas platyrhynchos*	Aves	Anseriformes	Anatidae
ENSMGAP00000010781	AQP 4	Turkey	*Meleagris gallopavo*	Aves	Galliformes	Phasianidae
AAL73511	AQP 4	Common quail	*Coturnix coturnix*	Aves	Galliformes	Phasianidae
ENSGALP00000024367	AQP 4	Chicken	*Gallus gallus*	Aves	Galliformes	Phasianidae
AAWZ02009571/ENSACAP00000002099	AQP 4	Green anole	*Anolis carolinensis*	Lepidosauria	Squamata	Iguanidae
AAMC02021699/ENSXETP00000061647	AQP 4	Western clawed frog	*Xenopus (Silurana) tropicalis*	Amphibia	Anura	Pipidae
AFYH01162813/AFYH01162814/AFYH01162815	Aqp 4	Coelacanth	*Latimeria chalumnae*	Actinistia	Coelacanthiformes	Coelacanthidae
ENSTRUP00000021667	Aqp 4a	Torafugu	*Takifugu rubripes*	Acanthopterygii	Tetraodontiformes	Tetraodontidae
ENSTNIP00000018275	Aqp 4a	Green-spotted pufferfish	*Tetraodon nigroviridis*	Acanthopterygii	Tetraodontiformes	Tetraodontidae
FM156410	Aqp 4a	Gilthead seabream	*Sparus aurata*	Acanthopterygii	Perciformes	Sparidae
FM027211/FM008510	Aqp 4a	European seabass	*Dicentrarchus labrax*	Acanthopterygii	Perciformes	Moronidae
ENSGACP00000017439	Aqp 4a	Three-spined stickleback	*Gasterosteus aculeatus*	Acanthopterygii	Gasterosteiformes	Gasterosteidae
ENSORLP00000021288	Aqp 4a	Japanese medaka	*Oryzias latipes*	Acanthopterygii	Beloniformes	Adrianichthyidae
BX885214/CCAF010050670	Aqp 4a1	Rainbow trout	*Oncorhynchus mykiss*	Protacanthopterygii	Salmoniformes	Salmonidae
FJ666327/ENSDARP00000021578	Aqp 4b	Zebrafish	*Danio rerio*	Ostariophysi	Cypriniformes	Cyprinidae
AEJ08190	Aqp 4	Spiny dogfish	*Squalus acanthias*	Chondrichthyes	Squaliformes	Squalidae
AAVX02013228	Aqp 4	Ghost shark	*Callorhinchus milii*	Chondrichthyes	Chimaeriformes	Callorhinchidae
ENSPMAP00000008913	Aqp 4	Sea lamprey	*Petromyzon marinus*	Hyperoartia	Petromyzontiformes	Petromyzontidae
BAE93686	Aqp 4	Inshore hagfish	*Eptatretus burgeri*	Hyperotreti	Myxiniformes	Myxinidae
ENSCINP00000007510	Aqp 4L	Vase tunicate	*Ciona intestinalis*	Tunicata	Enterogona	Cionidae
XP_002612400/ABEP02002539/FE561739/ XP_002213012/XP_002215983	Aqp 4L1	Florida lancelet	*Branchiostoma floridae*	Cephalochordata	Amphioxiformes	Branchiostomidae
XP_001185961/AAGJ04084831/AAGJ04084830/ SPU_012222-tr	Aqp 4L1	Purple sea urchin	*Strongylocentrotus purpuratus*	Echinodermata	Echinozoa	Strongylocentrotidae
XP_003727808/JT111244/AAGJ04146718/ SPU_021387-tr	Aqp 4L3	Purple sea urchin	*Strongylocentrotus purpuratus*	Echinodermata	Echinozoa	Strongylocentrotidae
**Protostomia Aqp4 orthologs**						
**Arthropoda Bib**						
FBpp0079519	Bib	Fruit fly	*Drosophila melanogaster*	Hexapoda	Diptera	Drosophilidae
FBpp0222049	Bib	Fruit fly	*Drosophila simulans*	Hexapoda	Diptera	Drosophilidae
FBpp0199328	Bib	Fruit fly	*Drosophila sechellia*	Hexapoda	Diptera	Drosophilidae
FBpp0263898	Bib	Fruit fly	*Drosophila yakuba*	Hexapoda	Diptera	Drosophilidae
FBpp0128621	Bib	Fruit fly	*Drosophila erecta*	Hexapoda	Diptera	Drosophilidae
FBpp0118926	Bib	Fruit fly	*Drosophila ananassae*	Hexapoda	Diptera	Drosophilidae
FBpp0280277	Bib	Fruit fly	*Drosophila pseudoobscura*	Hexapoda	Diptera	Drosophilidae
FBpp0183059	Bib	Fruit fly	*Drosophila persimilis*	Hexapoda	Diptera	Drosophilidae
ABL85281	Bib	Fruit fly	*Drosophila americana*	Hexapoda	Diptera	Drosophilidae
FBpp0232341	Bib	Fruit fly	*Drosophila virilis*	Hexapoda	Diptera	Drosophilidae
FBpp0166799	Bib	Fruit fly	*Drosophila mojavensis*	Hexapoda	Diptera	Drosophilidae
FBpp0144970	Bib	Fruit fly	*Drosophila grimshawi*	Hexapoda	Diptera	Drosophilidae
FBpp0244725/FBpp0241572	Bib	Fruit fly	*Drosophila willistoni*	Hexapoda	Diptera	Drosophilidae
AFP49901	Bib	Tsetse fly	*Glossina morsitans morsitans*	Hexapoda	Diptera	Glossinidae
XP_004534536	Bib	Mediterranean fruit fly	*Ceratitis capitata*	Hexapoda	Diptera	Tephritidae
AAEL004741/XP_001649747	Bib	Yellow fever mosquito	*Aedes aegypti*	Hexapoda	Diptera	Culicidae
CPIJ016447	Bib	Southern house mosquito	*Culex quinquefasciatus*	Hexapoda	Diptera	Culicidae
AGAP008766/AGAP008767	Bib	African malaria mosquito	*Anopheles gambiae*	Hexapoda	Diptera	Culicidae
EFR20654/ETN57779	Bib	American malaria mosquito	*Anopheles darlingi*	Hexapoda	Diptera	Culicidae
GAAK01005117/GAAK01001577	Bib	Antarctic flightless midge	*Belgica antarctica*	Hexapoda	Diptera	Chironomidae
KA181207	Bib	Harlequin fly	*Chironomus riparius*	Hexapoda	Diptera	Chironomidae
AADK01007500/BABH01027418/BABH01043412	Bib	Domestic silkworm	*Bombyx mori*	Hexapoda	Lepidoptera	Bombycidae
CAEZ01006773	Bib	Postman butterfly	*Heliconius melpomene melpomene*	Hexapoda	Lepidoptera	Nymphalidae
EHJ64738/EHJ63154	Bib	Monarch butterfly	*Danaus plexippus*	Hexapoda	Lepidoptera	Nymphalidae
AIXA01000727/AIXA01000726/AIXA01003168	Bib	Tobacco hornworm	*Manduca sexta*	Hexapoda	Lepidoptera	Sphingidae
TC010832	Bib	Red flour Beetle	*Tribolium castaneum*	Hexapoda	Coleoptera	Tenebrionidae
JU421678	Bib	Salt marsh beetle	*Pogonus chalceus*	Hexapoda	Coleoptera	Carabidae
JR479665	Bib	Red palm weevil	*Rhynchophorus ferrugineus*	Hexapoda	Coleoptera	Curculionidae
ENN74136	Bib	Mountain pine beetle	*Dendroctonus ponderosae*	Hexapoda	Coleoptera	Curculionidae
XP_003399074	Bib	Buff-tailed bumblebee	*Bombus terrestris*	Hexapoda	Hymenoptera	Apidae
XP_003487862	Bib	Common eastern bumble bee	*Bombus impatiens*	Hexapoda	Hymenoptera	Apidae

XP_396705	Bib	Honey bee	*Apis mellifera*	Hexapoda	Hymenoptera	Apidae
XP_003699287	Bib	Alfalfa leafcutting bee	*Megachile rotundata*	Hexapoda	Hymenoptera	Megachilidae
EFZ15059	Bib	Red fire ant	*Solenopsis invicta*	Hexapoda	Hymenoptera	Formicidae
AEAB01004397	Bib	Florida carpenter ant	*Camponotus floridanus*	Hexapoda	Hymenoptera	Formicidae
EFN86731/EFN86732	Bib	Jerdon's jumping ant	*Harpegnathos saltator*	Hexapoda	Hymenoptera	Formicidae
EZA50845	Bib	Clonal raider ant	*Cerapachys biroi*	Hexapoda	Hymenoptera	Formicidae
EGI69716/AEVX01004595	Bib	Panamanian leafcutter ant	*Acromyrmex echinatior*	Hexapoda	Hymenoptera	Formicidae
ACEP_00006876	Bib	Leafcutter ant	*Atta cephalotes*	Hexapoda	Hymenoptera	Formicidae
XP_001604170	Bib	Jewel wasp	*Nasonia vitripennis*	Hexapoda	Hymenoptera	Pteromalidae
ADAO01027634/ADAO01027617	Bib	Jewel wasp	*Nasonia giraulti*	Hexapoda	Hymenoptera	Pteromalidae
XP_002433221	Bib	Human body louse	*Pediculus humanus corporis*	Hexapoda	Phthiraptera	Pediculidae
ACPB02031565/ACPB02009321	Bib	Assassin bug	*Rhodnius prolixus*	Hexapoda	Hemiptera	Reduviidae
XP_001948407	Bib	Pea aphid	*Acyrthosiphon pisum*	Hexapoda	Hemiptera	Aphididae
GACJ01023397	Bib	Asian citrus psyllid	*Diaphorina citri*	Hexapoda	Hemiptera	Psyllidae
JPZV01159195/JPZV01159181/JPZV01159174	Bib	German cockroach	*Blattella germanica*	Hexapoda	Blattodea	Ectobiidae
GAWS01120948	Bib	American cockroach	*Periplaneta americana*	Hexapoda	Blattodea	Blattidae
GAYD01311266	Bib	Blaberus cockroach	*Blaberus atropos*	Hexapoda	Blattodea	Blaberidae
GAZN01184473	Bib	Brown hooded cockroach	*Cryptocercus wrighti*	Hexapoda	Blattodea	Cryptocercidae
GASE01250065	Bib	Cuban subterranean termite	*Prorhinotermes simplex*	Hexapoda	Isoptera	Rhinotermitidae
AUST01019796/AUST01019797	Bib	Nevada dampwood termite	*Zootermopsis nevadensis*	Hexapoda	Isoptera	Termopsidae
GASW01145900	Bib	Praying mantis	*Mantis religiosa*	Hexapoda	Mantodea	Mantidae
GAYA01368224	Bib	Zoraptid	*Zorotypus caudelli*	Hexapoda	Orthoptera	Zorotypidae
GAWU01257959	Bib	Webspinner	*Aposthonia japonica*	Hexapoda	Embioptera	Oligotomidae
GAWG01145562	Bib	Giant prickly stick insect	*Extatosoma tiaratum*	Hexapoda	Phasmatodea	Phasmatidae
GAWC01014738	Bib	Thorny stick insect	*Aretaon asperrimus*	Hexapoda	Phasmatodea	Heteropterygidae
GAUX01120890	Bib	Camel cricket	*Ceuthophilus sp.*	Hexapoda	Orthoptera	Rhaphidophoridae
GAZT01171842	Bib	False stick insect	*Prosarthria teretrirostris*	Hexapoda	Orthoptera	Proscopiidae
GBHB01007420	Bib	Oceanic field cricket	*Teleogryllus commodus*	Hexapoda	Orthoptera	Gryllidae
GASQ01013559	Bib	Slender Groundhopper	*Tetrix subulata*	Hexapoda	Orthoptera	Tetrigidae
GAUZ01380599	Bib	Stripe-winged grasshopper	*Stenobothrus lineatus*	Hexapoda	Orthoptera	Acrididae
AVCP010869913	Bib	Migratory locust	*Locusta migratoria*	Hexapoda	Orthoptera	Acrididae
GATU01061203	Bib	Blue-winged olive	*Baetis sp.*	Hexapoda	Ephemeroptera	Baetidae
GAXA01112850	Bib	Mahogany Dun	*Isonychia bicolor*	Hexapoda	Ephemeroptera	Isonychiidae
GAZG01101625	Bib	Mayfly	*Eurylophella sp.*	Hexapoda	Ephemeroptera	Ephemerellidae
AYNC01046590/AYNC01046589	Bib	Green drake	*Ephemera danica*	Hexapoda	Ephemeroptera	Ephemeridae
GAYO01122019	Bib	Golden-ringed dragonfly	*Cordulegaster boltonii*	Hexapoda	Odonata	Cordulegastridae
APVN01095476/APVN01095473	Bib	Scarce chaser	*Ladona fulva*	Hexapoda	Odonata	Libellulidae
GASN01409838	Bib	Firebrat	*Thermobia domestica*	Hexapoda	Zygentoma	Lepismatidae
GAYJ01257237	Bib	Silverfish	*Atelura formicaria*	Hexapoda	Zygentoma	Nicoletiidae
GASO01228638	Bib	Silverfish	*Tricholepidion gertschi*	Hexapoda	Zygentoma	Libellulidae
GAUM01007766	Bib	Bristletail	*Machilis hrabei*	Hexapoda	Archaeognatha	Machilidae
GAUG01018425	Bib	Bristletail	*Meinertellus cundinamarcensis*	Hexapoda	Archaeognatha	Meinertellidae
GAXJ01108916	Bib	Two-pronged bristletail	*Occasjapyx japonicus*	Hexapoda	Diplura	Japygidae
GAXE01131619	Bib	Conehead	*Acerentomon sp.*	Hexapoda	Protura	Acerentomidae
AFFK01019694/AFFK01019695/SMAR015755	Bib	Coastal European centipede	*Strigamia maritima*	Chilopoda	Geophilomorpha	Linotaeniidae
GAFS01004822/GAFY01027786	Bib	Narrow-clawed crayfish	*Pontastacus leptodactylus*	Malacostraca	Decapoda	Astacidae
GADE01005115	Bib	Australian red claw crayfish	*Cherax quadricarinatus*	Malacostraca	Decapoda	Parastacidae
FE778057	Bib	Flat porcelain crab	*Petrolisthes cinctipes*	Malacostraca	Decapoda	Porcellanidae
DV467482	Bib	Green shore Crab	*Carcinus maenas*	Malacostraca	Decapoda	Carcinidae
JW508536	Bib	Tide pool copepod	*Tigriopus californicus*	Maxillopoda	Harpacticoida	Harpacticidae
JW965321	Bib	Argulus	*Argulus siamensis*	Maxillopoda	Arguloida	Argulidae
EFX82861	Bib	Common water flea	*Daphnia pulex*	Branchiopoda	Diplostraca	Daphniidae
XP_002408745/XP_002408744	Bib	Black-legged tick	*Ixodes scapularis*	Acari	Ixodida	Ixodidae
GACK01002509	Bib	Zebra tick	*Rhipicephalus pulchellus*	Acari	Ixodida	Ixodidae
CK185295	Bib	Southern cattle tick	*Rhipicephalus microplus*	Acari	Ixodida	Ixodidae
GW003922	Bib	Two-spotted spider mite	*Tetranychus urticae*	Acari	Acariformes	Tetranychidae
AZAQ01093526/AZAQ01093525/AZAQ01093524/ GAZR01022102	Bib	African social eresid spider	*Stegodyphus mimosarum*	Arachnida	Araneae	Eresidae
GBCS01010810	Bib	Western black widow	*Latrodectus hesperus*	Arachnida	Araneae	Theridiidae
AOMJ01042817/AOMJ01089836/AOMJ01042847/ AOMJ01042849	Bib	Common house spider	*Parasteatoda tepidariorum*	Arachnida	Araneae	Theridiidae
AYEL01087177	Bib	Chinese scorpion	*Mesobuthus martensii*	Arachnida	Scorpiones	Buthidae
AXZI01186657	Bib	Baja California bark scorpion	*Centruroides exilicauda*	Arachnida	Scorpiones	Buthidae

Lepidoptera Aqp

BGIBMGA008236	Aqp	Domestic silkworm	*Bombyx mori*	Hexapoda	Lepidoptera	Bombycidae
EHJ68353	Aqp	Monarch butterfly	*Danaus plexippus*	Hexapoda	Lepidoptera	Nymphalidae

Hexapoda Drip

FBpp0087240	Drip	Fruit fly	*Drosophila melanogaster*	Hexapoda	Diptera	Drosophilidae
FBpp0201903	Drip	Fruit fly	*Drosophila sechellia*	Hexapoda	Diptera	Drosophilidae
FBpp0258530	Drip	Fruit fly	*Drosophila yakuba*	Hexapoda	Diptera	Drosophilidae
FBpp0141192	Drip	Fruit fly	*Drosophila erecta*	Hexapoda	Diptera	Drosophilidae
FBpp0115586	Drip	Fruit fly	*Drosophila ananassae*	Hexapoda	Diptera	Drosophilidae
FBpp0276838	Drip	Fruit fly	*Drosophila pseudoobscura*	Hexapoda	Diptera	Drosophilidae
FBpp0236388	Drip	Fruit fly	*Drosophila virilis*	Hexapoda	Diptera	Drosophilidae
FBpp0170266	Drip	Fruit fly	*Drosophila mojavensis*	Hexapoda	Diptera	Drosophilidae
FBpp0154409	Drip	Fruit fly	*Drosophila grimshawi*	Hexapoda	Diptera	Drosophilidae
FBpp0251027	Drip	Fruit fly	*Drosophila willistoni*	Hexapoda	Diptera	Drosophilidae
BAM26200	Drip	Black blowfly	*Phormia regina*	Hexapoda	Diptera	Calliphoridae
AEG47703	Drip	Oriental latrine fly	*Chrysomya megacephala*	Hexapoda	Diptera	Calliphoridae
Q25074	Drip	Buffalo fly	*Haematobia irritans exigua*	Hexapoda	Diptera	Muscidae
ADD19102	Drip 1	Tsetse fly	*Glossina morsitans morsitans*	Hexapoda	Diptera	Glossinidae
ADD20051	Drip 2	Tsetse fly	*Glossina morsitans morsitans*	Hexapoda	Diptera	Glossinidae
FG293007/FG295048	Drip	Primary screw-worm	*Cochliomyia hominivorax*	Hexapoda	Diptera	Calliphoridae
AAEL003512/XP_001656931	Drip	Yellow fever mosquito	*Aedes aegypti*	Hexapoda	Diptera	Culicidae
CPIJ015704/CPIJ015704	Drip	Southern house mosquito	*Culex quinquefasciatus*	Hexapoda	Diptera	Culicidae
JAA93938	Drip	Psorophora mosquito	*Psorophora albipes*	Hexapoda	Diptera	Culicidae
AGAP008842	Drip	African malaria mosquito	*Anopheles gambiae*	Hexapoda	Diptera	Culicidae
EZ976133	Drip	African malaria mosquito	*Anopheles funestus*	Hexapoda	Diptera	Culicidae
GAAK01006851/GAAK01006849	Drip	Antarctic flightless midge	*Belgica antarctica*	Hexapoda	Diptera	Chironomidae
ABV60346	Drip	Sand fly	*Lutzomyia longipalpis*	Hexapoda	Diptera	Psychodidae
GAKJ01008613	Drip	Orange wheat blossom midge	*Sitodiplosis mosellana*	Hexapoda	Diptera	Cecidomyiidae
AB178640	Drip	Domestic silkworm	*Bombyx mori*	Hexapoda	Lepidoptera	Bombycidae
CAEZ01007264/CAEZ01007265	Drip	Postman butterfly	*Heliconius melpomene melpomene*	Hexapoda	Lepidoptera	Nymphalidae
EHJ75085	Drip	Monarch butterfly	*Danaus plexippus*	Hexapoda	Lepidoptera	Nymphalidae
FQ019249	Drip	African cotton leafworm	*Spodoptera littoralis*	Hexapoda	Lepidoptera	Noctuidae
GAFU01003217	Drip	Beet armyworm	*Spodoptera exigua*	Hexapoda	Lepidoptera	Noctuidae
HO053923	Drip	Tobacco budworm	*Heliothis virescens*	Hexapoda	Lepidoptera	Noctuidae
EZ583816	Drip	western bean cutworm	*Striacosta albicosta*	Hexapoda	Lepidoptera	Noctuidae
BAM19007	Drip	Common mormon	*Papilio polytes*	Hexapoda	Lepidoptera	Papilionidae

BAM17858	Drip	Asian Swallowtail	*Papilio xuthus*	Hexapoda	Lepidoptera	Papilionidae
AFC34081	Drip	striped riceborer	*Chilo suppressalis*	Hexapoda	Lepidoptera	Crambidae
JP612973	Drip	Propertius duskywing	*Erynnis propertius*	Hexapoda	Lepidoptera	Hesperiidae
JO817751/GR920919	Drip	Tobacco hornworm	*Manduca sexta*	Hexapoda	Lepidoptera	Sphingidae
BAH47554	Drip	Oriental fruit moth	*Grapholita molesta*	Hexapoda	Lepidoptera	Tortricidae
TC011257	Drip	Red flour Beetle	*Tribolium castaneum*	Hexapoda	Coleoptera	Tenebrionidae
BAM83568	Drip	Beetle	*Anomala cuprea*	Hexapoda	Coleoptera	Scarabaeidae
JR487579	Drip	Red palm weevil	*Rhynchophorus ferrugineus*	Hexapoda	Coleoptera	Curculionidae
AEE63193	Drip	Mountain pine weevil	*Dendroctonus ponderosae*	Hexapoda	Coleoptera	Curculionidae
JU414336	Drip	Salt marsh beetle	*Pogonus chalceus*	Hexapoda	Coleoptera	Carabidae
GAPE01027420	Drip	Pollen beetle	*Brassicogethes aeneus*	Hexapoda	Coleoptera	Nitidulidae
GAAB01000751	Drip	Emerald ash borer	*Agrilus planipennis*	Hexapoda	Coleoptera	Buprestidae
GAXW01087449	Drip	Antlion	*Euroleon nostras*	Hexapoda	Neoptera	Myrmeleontidae
GAVV01176955	Drip	Green lacewing	*Pseudomallada prasinus*	Hexapoda	Neoptera	Chrysopidae
XP_003394164	Drip	Buff-tailed bumblebee	*Bombus terrestris*	Hexapoda	Hymenoptera	Apidae
XP_003487533	Drip	Common eastern bumble bee	*Bombus impatiens*	Hexapoda	Hymenoptera	Apidae
XP_624531	Drip	Honey bee	*Apis mellifera*	Hexapoda	Hymenoptera	Apidae
XP_003701708	Drip	Alfalfa leafcutting bee	*Megachile rotundata*	Hexapoda	Hymenoptera	Megachilidae
AEAQ01012360/EFZ22501	Drip	Red fire ant	*Solenopsis invicta*	Hexapoda	Hymenoptera	Formicidae
AEAB01004718/EFN72836	Drip	Florida carpenter ant	*Camponotus floridanus*	Hexapoda	Hymenoptera	Formicidae
JP783671/JP783671	Drip	Caribbean crazy ant	*Nylanderia pubens*	Hexapoda	Hymenoptera	Formicidae
ADOQ01012968	Drip	Argentine ant	*Linepithema humile*	Hexapoda	Hymenoptera	Formicidae
XP_001607940	Drip	Jewel wasp	*Nasonia vitripennis*	Hexapoda	Hymenoptera	Pteromalidae
GAKG01000818	Drip	Diamondback moth parasitoid	*Cotesia vestalis*	Hexapoda	Hymenoptera	Braconidae
EEB12655	Drip	Human body louse	*Pediculus humanus corporis*	Hexapoda	Phthiraptera	Pediculidae
GAWR01000696	Drip	Poultry shaft louse	*Menopon gallinae*	Hexapoda	Phthiraptera	Menoponidae
GAYV01109739	Drip	Booklice	*Liposcelis bostrychophila*	Hexapoda	Psocoptera	Liposcelidae
GAPT01006904	Drip	Booklice	*Ectopsocus briggsi*	Hexapoda	Psocoptera	Ectopsocidae
GAXD01024860	Drip	Western flower thrips	*Frankliniella occidentalis*	Hexapoda	Thysanoptera	Thripidae
JAA75980	Drip	Assassin bug	*Rhodnius prolixus*	Hexapoda	Hemiptera	Reduviidae
KF048092	Drip	Lygus bug	*Lygus hesperus*	Hexapoda	Hemiptera	Miridae
GAJX01005354	Drip	Pod sucking bug	*Clavigralla tomentosicollis*	Hexapoda	Hemiptera	Coreidae
GAJW01004051	Drip	Cowpea aphid	*Aphis craccivora*	Hexapoda	Hemiptera	Aphididae
ACL01373	Drip	Pea aphid	*Acyrthosiphon pisum*	Hexapoda	Hemiptera	Aphididae
GAAF01000345	Drip	Potato aphid	*Macrosiphum euphorbiae*	Hexapoda	Hemiptera	Aphididae
EE571220	Drip	Green peach aphid	*Myzus persicae*	Hexapoda	Hemiptera	Aphididae
FO035865	Drip	Shallot aphid	*Myzus ascalonicus*	Hexapoda	Hemiptera	Aphididae
ABW96354	Drip	Sweet potato whitefly	*Bemisia tabaci*	Hexapoda	Hemiptera	Aleyrodidae
Q23808	Drip	Green leafhopper	*Cicadella viridis*	Hexapoda	Hemiptera	Cicadellidae
GAGF01044362	Drip	Green lacewing	*Chrysopa pallens*	Hexapoda	Neoptera	Chrysopidae
JPZV01194828	Drip	German cockroach	*Blattella germanica*	Hexapoda	Blattodea	Ectobiidae
GAWS01023831	Drip	American cockroach	*Periplaneta americana*	Hexapoda	Blattodea	Blattidae
GAYD01018072	Drip	Blaberus cockroach	*Blaberus atropos*	Hexapoda	Blattodea	Blaberidae
GAZN01021910	Drip	Brown hooded cockroach	*Cryptocercus wrighti*	Hexapoda	Blattodea	Cryptocercidae
AUST01002328/AUST01002331/AUST01002332	Drip	Nevada dampwood termite	*Zootermopsis nevadensis*	Hexapoda	Isoptera	Termopsidae
BAG72254	Drip	Formosan subterranean termite	*Coptotermes formosanus*	Hexapoda	Isoptera	Rhinotermitidae
GASE01015407	Drip 1	Cuban subterranean termite	*Prorhinotermes simplex*	Hexapoda	Isoptera	Rhinotermitidae
GASE01015406	Drip 2	Cuban subterranean termite	*Prorhinotermes simplex*	Hexapoda	Isoptera	Rhinotermitidae
FL637621/GO899611	Drip	Eastern subterranean termite	*Reticulitermes flavipes*	Hexapoda	Isoptera	Rhinotermitidae
GATB01242877	Drip	Metallyticid mantis	*Metallyticus splendidus*	Hexapoda	Mantodea	Metallyticidae
GASW01221493	Drip	Praying mantis	*Mantis religiosa*	Hexapoda	Mantodea	Mantidae
GAYA01397859	Drip	Zoraptid	*Zorotypus caudelli*	Hexapoda	Orthoptera	Zorotypidae
GAYQ01184451	Drip	European earwig	*Forficula auricularia*	Hexapoda	Dermaptera	Forficulidae
GAUF01006901	Drip	Leuctra	*Leuctra sp.*	Hexapoda	Plecoptera	Leuctridae
GATV01089684	Drip	Stonefly	*Perla marginata*	Hexapoda	Plecoptera	Perlidae
GAWU01021632	Drip	Webspinner	*Aposthonia japonica*	Hexapoda	Embioptera	Oligotomidae
GAWG01027137	Drip	Giant prickly stick insect	*Extatosoma tiaratum*	Hexapoda	Phasmatodea	Phasmatidae
GAWE01111259	Drip	Vietnamese walking stick insect	*Ramulus artemis*	Hexapoda	Phasmatodea	Phasmatidae
GAWD01031379	Drip	Vietnamese walking stick	*Medauroidea extradentata*	Hexapoda	Phasmatodea	Phasmatidae
GAWC01082932	Drip	Thorny stick insect	*Aretaon asperrimus*	Hexapoda	Phasmatodea	Heteropterygidae
GAXB01016476	Drip	Heelwalker	*Tanzaniophasma sp.*	Hexapoda	Mantophasmatodea	Tanzaniophasmatidae
GAUX01016809	Drip	Camel cricket	*Ceuthophilus sp.*	Hexapoda	Orthoptera	Rhaphidophoridae
GAZT01011867	Drip	False stick insect	*Prosarthria teretrirostris*	Hexapoda	Orthoptera	Proscopiidae
GAIZ01003265/GAIZ01000499	Drip	Sand field cricket	*Gryllus firmus*	Hexapoda	Orthoptera	Gryllidae
GBHB01065040	Drip	Oceanic field cricket	*Teleogryllus commodus*	Hexapoda	Orthoptera	Gryllidae
GASQ01128475	Drip	Slender Groundhopper	*Tetrix subulata*	Hexapoda	Orthoptera	Tetrigidae
JG680985	Drip	Desert locust	*Schistocerca gregaria*	Hexapoda	Orthoptera	Acrididae
CO850494/AVCP010961306	Drip	Migratory locust	*Locusta migratoria*	Hexapoda	Orthoptera	Acrididae
GAUZ01033299	Drip	Stripe-winged grasshopper	*Stenobothrus lineatus*	Hexapoda	Orthoptera	Acrididae
GAXA01100045	Drip	Mahogany Dun	*Isonychia bicolor*	Hexapoda	Ephemeroptera	Isonychiidae
GAZG01016414	Drip	Mayfly	*Eurylophella sp.*	Hexapoda	Ephemeroptera	Ephemerellidae
AYNC01036599/AYNC01036594/AYNC01036593/ AYNC01036592	Drip	Green drake	*Ephemera danica*	Hexapoda	Ephemeroptera	Ephemeridae
GAYO01010968	Drip	Golden-ringed dragonfly	*Cordulegaster boltonii*	Hexapoda	Odonata	Cordulegastridae
GAEQ01000128	Drip 1	Hagen´s bluet	*Enallagma hageni*	Hexapoda	Odonata	Coenagrionidae
GAEQ01002752	Drip 2	Hagen´s bluet	*Enallagma hageni*	Hexapoda	Odonata	Coenagrionidae
APVN01111337/APVN01111338/APVN01111340	Drip	Scarce chaser	*Ladona fulva*	Hexapoda	Odonata	Libellulidae
GASN01407843	Drip	Firebrat	*Thermobia domestica*	Hexapoda	Zygentoma	Lepismatidae
GAYJ01029112	Drip	Silverfish	*Atelura formicaria*	Hexapoda	Zygentoma	Nicoletiidae
GASO01258000	Drip	Silverfish	*Tricholepidion gertschi*	Hexapoda	Zygentoma	Libellulidae
GAUM01185038	Drip	Bristletail	*Machilis hrabei*	Hexapoda	Archaeognatha	Machilidae
GAUG01019014	Drip	Bristletail	*Meinertellus cundinamarcensis*	Hexapoda	Archaeognatha	Meinertellidae
GAYN01143457	Drip	Campodea	*Campodea augens*	Hexapoda	Diplura	Campodeidae
GAMM01004264/GAMM01007190	Drip	Springtail	*Orchesella cincta*	Hexapoda	Collembola	Entomobryidae
EV475341/GASX01090561/GAMN01000162	Drip	Springtail	*Folsomia candida*	Hexapoda	Collembola	Isotomidae
sb_006_05H07	Drip -like	Springtail	*Megaphorura arctica*	Hexapoda	Collembola	Onychiuridae
GATZ01010361	Drip -like	Clover springtail	*Sminthurus viridis*	Hexapoda	Collembola	Sminthuridae
GAUE01009798	Drip -like	Cosmopolitan springtail	*Anurida maritima*	Hexapoda	Collembola	Neanuridae
GAXI01003650	Drip -like	Giant springtail	*Tetrodontophora bielanensis*	Hexapoda	Collembola	Onychiuridae
GATD01013001	Drip -like	Springtail	*Pogonognathellus sp.*	Hexapoda	Collembola	Tomoceridae
**Arthropoda Prip**						
FBpp0087236	Prip	Fruit fly	*Drosophila melanogaster*	Hexapoda	Diptera	Drosophilidae
FBpp0202768	Prip	Fruit fly	*Drosophila sechellia*	Hexapoda	Diptera	Drosophilidae
FBpp0209200	Prip	Fruit fly	*Drosophila simulans*	Hexapoda	Diptera	Drosophilidae
FBpp0257373	Prip	Fruit fly	*Drosophila yakuba*	Hexapoda	Diptera	Drosophilidae
FBpp0138749	Prip	Fruit fly	*Drosophila erecta*	Hexapoda	Diptera	Drosophilidae
FBpp0115747	Prip	Fruit fly	*Drosophila ananassae*	Hexapoda	Diptera	Drosophilidae
FBpp0277602	Prip	Fruit fly	*Drosophila pseudoobscura*	Hexapoda	Diptera	Drosophilidae
FBpp0234775	Prip	Fruit fly	*Drosophila virilis*	Hexapoda	Diptera	Drosophilidae

FBpp0167781	Prip	Fruit fly	*Drosophila mojavensis*	Hexapoda	Diptera	Drosophilidae
FBpp0155293	Prip	Fruit fly	*Drosophila grimshawi*	Hexapoda	Diptera	Drosophilidae
FBpp0250560	Prip	Fruit fly	*Drosophila willistoni*	Hexapoda	Diptera	Drosophilidae
ACT34032	Prip	Goldenrod gall fly	*Eurosta solidaginis*	Hexapoda	Diptera	Tephritidae
ADD19396	Prip 1	Tsetse fly	*Glossina morsitans morsitans*	Hexapoda	Diptera	Glossinidae
AFP49895	Prip 2	Tsetse fly	*Glossina morsitans morsitans*	Hexapoda	Diptera	Glossinidae
AAEL003550/XP_001656932	Prip	Yellow fever mosquito	*Aedes aegypti*	Hexapoda	Diptera	Culicidae
CPIJ015700	Prip	Southern house mosquito	*Culex quinquefasciatus*	Hexapoda	Diptera	Culicidae
AGAP008843	Prip	African malaria mosquito	*Anopheles gambiae*	Hexapoda	Diptera	Culicidae
BAF62090	Prip	Sleeping chironomid	*Polypedilum vanderplanki*	Hexapoda	Diptera	Chironomidae
BAK32937/BAK32936/BAK32935/GAAK01006838	Prip 1	Antarctic flightless midge	*Belgica antarctica*	Hexapoda	Diptera	Chironomidae
GAAK01006838	Prip 2	Antarctic flightless midge	*Belgica antarctica*	Hexapoda	Diptera	Chironomidae
NP_001153661	Prip	Domestic silkworm	*Bombyx mori*	Hexapoda	Lepidoptera	Bombycidae
CAEZ01007983	Prip	Postman butterfly	*Heliconius melpomene melpomene*	Hexapoda	Lepidoptera	Nymphalidae
EL599805/EL596094	Prip	Red postman	*Heliconius erato*	Hexapoda	Lepidoptera	Nymphalidae
EHJ66754	Prip	Monarch butterfly	*Danaus plexippus*	Hexapoda	Lepidoptera	Nymphalidae
EZ981212	Prip	African cotton leafworm	*Spodoptera littoralis*	Hexapoda	Lepidoptera	Noctuidae
GAFU01006086	Prip	Beet armyworm	*Spodoptera exigua*	Hexapoda	Lepidoptera	Noctuidae
JP717913	Prip	Asian Swallowtail	*Papilio xuthus*	Hexapoda	Lepidoptera	Papilionidae
GAJS01000346	Prip	striped riceborer	*Chilo suppressalis*	Hexapoda	Lepidoptera	Crambidae
JP612966	Prip	Propertius duskywing	*Erynnis propertius*	Hexapoda	Lepidoptera	Hesperiidae
JO818733	Prip	Tobacco hornworm	*Manduca sexta*	Hexapoda	Lepidoptera	Sphingidae
TC001374	Prip	Red flour Beetle	*Tribolium castaneum*	Hexapoda	Coleoptera	Tenebrionidae
AEE61850	Prip	Mountain pine weevil	*Dendroctonus ponderosae*	Hexapoda	Coleoptera	Curculionidae
JU408944	Prip	Salt marsh beetle	*Pogonus chalceus*	Hexapoda	Coleoptera	Carabidae
AAL09065	Prip	Firefly	*Pyrocoelia rufa*	Hexapoda	Coleoptera	Lampyridae
GAXW01003825	Prip	Antlion	*Euroleon nostras*	Hexapoda	Neoptera	Myrmeleontidae
GAVV01005429	Prip	Green lacewing	*Pseudomallada prasinus*	Hexapoda	Neoptera	Chrysopidae
XP_003394168/XP_003394169	Prip	Buff-tailed bumblebee	*Bombus terrestris*	Hexapoda	Hymenoptera	Apidae
XP_003487537/XP_003487538	Prip	Common eastern bumble bee	*Bombus impatiens*	Hexapoda	Hymenoptera	Apidae
XP_394391	Prip	Honey bee	*Apis mellifera*	Hexapoda	Hymenoptera	Apidae
XP_003701667	Prip	Alfalfa leafcutting bee	*Megachile rotundata*	Hexapoda	Hymenoptera	Megachilidae
GAFR01021571	Prip	Neotropical paper wasp	*Polistes canadensis*	Hexapoda	Hymenoptera	Vespidae
EFN88447	Prip	Jerdon's jumping ant	*Harpegnathos saltator*	Hexapoda	Hymenoptera	Formicidae
EFN72835	Prip	Florida carpenter ant	*Camponotus floridanus*	Hexapoda	Hymenoptera	Formicidae
ACEP_00007751	Prip	Leafcutter ant	*Atta cephalotes*	Hexapoda	Hymenoptera	Formicidae
XP_001607929	Prip	Jewel wasp	*Nasonia vitripennis*	Hexapoda	Hymenoptera	Pteromalidae
ADAO01292418/ADAO01292417/ADAO01292415	Prip	Jewel wasp	*Nasonia giraulti*	Hexapoda	Hymenoptera	Pteromalidae
EEB16742	Prip	Human body louse	*Pediculus humanus corporis*	Hexapoda	Phthiraptera	Pediculidae
GAWR01000697	Eglp	Poultry shaft louse	*Menopon gallinae*	Hexapoda	Phthiraptera	Menoponidae
GAYV01110104	Eglp	Booklice	*Liposcelis bostrychophila*	Hexapoda	Psocoptera	Liposcelidae
GAPT01004386	Eglp	Booklice	*Ectopsocus briggsi*	Hexapoda	Psocoptera	Ectopsocidae
GAXD01020785	Prip	Western flower thrips	*Frankliniella occidentalis*	Hexapoda	Thysanoptera	Thripidae
GAHY01001529	Prip	Assassin bug	*Rhodnius prolixus*	Hexapoda	Hemiptera	Reduviidae
KF048099	Prip	Lygus bug	*Lygus hesperus*	Hexapoda	Hemiptera	Miridae
KF048100	Prip	Lygus bug	*Lygus hesperus*	Hexapoda	Hemiptera	Miridae
HP661479	Prip	Sweet potato whitefly	*Bemisia tabaci*	Hexapoda	Hemiptera	Aleyrodidae
AHB86600	Prip	potato/tomato psyllid	*Bactericera cockerelli*	Hexapoda	Hemiptera	Triozidae
DN195967	Prip	Glassy-winged sharpshooter	*Homalodisca vitripennis*	Hexapoda	Hemiptera	Cicadellidae
GAGF01006829	Prip	Green lacewing	*Chrysopa pallens*	Hexapoda	Neoptera	Chrysopidae
CBY77924/GBID01001247	Prip	German cockroach	*Blattella germanica*	Hexapoda	Blattodea	Ectobiidae
GAWS01258646	Prip	American cockroach	*Periplaneta americana*	Hexapoda	Blattodea	Blattidae
GAYD01029081	Prip	Blaberus cockroach	*Blaberus atropos*	Hexapoda	Blattodea	Blaberidae
GAZN01019011	Prip	Brown hooded cockroach	*Cryptocercus wrighti*	Hexapoda	Blattodea	Cryptocercidae
AUST01002325/AUST01002324	Prip	Nevada dampwood termite	*Zootermopsis nevadensis*	Hexapoda	Isoptera	Termopsidae
JK445261/JK445262	Prip	Formosan subterranean termite	*Coptotermes formosanus*	Hexapoda	Isoptera	Rhinotermitidae
GASE01253679	Prip	Cuban subterranean termite	*Prorhinotermes simplex*	Hexapoda	Isoptera	Rhinotermitidae
FL638365/GO907955	Prip	Eastern subterranean termite	*Reticulitermes flavipes*	Hexapoda	Isoptera	Rhinotermitidae
GATB01001403	Prip	Metallyticid mantis	*Metallyticus splendidus*	Hexapoda	Mantodea	Metallyticidae
GASW01019624	Prip	Praying mantis	*Mantis religiosa*	Hexapoda	Mantodea	Mantidae
GAYQ01017598	Prip	European earwig	*Forficula auricularia*	Hexapoda	Dermaptera	Forficulidae
GAYL01013696	Prip	Stonefly	*Cosmioperla kuna*	Hexapoda	Plecoptera	Eustheniidae
GAUF01007405	Prip	Leuctra	*Leuctra sp.*	Hexapoda	Plecoptera	Leuctridae
GAYL01013696	Prip	Stonefly	*Perla marginata*	Hexapoda	Plecoptera	Perlidae
GAWU01256075	Prip	Webspinner	*Aposthonia japonica*	Hexapoda	Embioptera	Oligotomidae
GAWG01042575	Prip	Giant prickly stick insect	*Extatosoma tiaratum*	Hexapoda	Phasmatodea	Phasmatidae
GAWE01078541	Prip	Vietnamese walking stick insect	*Ramulus artemis*	Hexapoda	Phasmatodea	Phasmatidae
GAWD01074919	Prip	Vietnamese walking stick	*Medauroidea extradentata*	Hexapoda	Phasmatodea	Phasmatidae
GAWF01050038/GAWF01050037	Prip	Pink winged stick insect	*Sipyloidea sipylus*	Hexapoda	Phasmatodea	Diapheromeridae
GAWC01082955	Prip	Thorny stick insect	*Aretaon asperrimus*	Hexapoda	Phasmatodea	Heteropterygidae
GAXB01016385	Prip	Heelwalker	*Tanzaniophasma sp.*	Hexapoda	Mantophasmatodea	Tanzaniophasmatidae
GAUX01030554	Prip	Camel cricket	*Ceuthophilus sp.*	Hexapoda	Orthoptera	Rhaphidophoridae
GAZT01006221	Prip	False stick insect	*Prosarthria teretrirostris*	Hexapoda	Orthoptera	Proscopiidae
GAIZ01018549	Prip	Sand field cricket	*Gryllus firmus*	Hexapoda	Orthoptera	Gryllidae
DC443641	Prip	Two-spotted cricket	*Gryllus bimaculatus*	Hexapoda	Orthoptera	Gryllidae
EH632028/EH639574	Prip	Hawaiian swordtail cricket	*Laupala kohalensis*	Hexapoda	Orthoptera	Gryllidae
GBHB01027253	Prip	Oceanic field cricket	*Teleogryllus commodus*	Hexapoda	Orthoptera	Gryllidae
GASQ01011894	Prip	Slender Groundhopper	*Tetrix subulata*	Hexapoda	Orthoptera	Tetrigidae
CO821055/CO821056	Prip	Migratory locust	*Locusta migratoria*	Hexapoda	Orthoptera	Acrididae
GAUZ01034179	Prip	Stripe-winged grasshopper	*Stenobothrus lineatus*	Hexapoda	Orthoptera	Acrididae
GATU01010497	Prip	Blue-winged olive	*Baetis sp.*	Hexapoda	Ephemeroptera	Baetidae
GAXA01011140	Prip	Mahogany Dun	*Isonychia bicolor*	Hexapoda	Ephemeroptera	Isonychiidae
GAZG01016497	Prip	Mayfly	*Eurylophella sp.*	Hexapoda	Ephemeroptera	Ephemerellidae
AYNC01078222	Prip	Green drake	*Ephemera danica*	Hexapoda	Ephemeroptera	Ephemeridae
GAYM01100186	Prip	Banded damoiselle	*Calopteryx splendens*	Hexapoda	Odonata	Calopterygidae
GAVW01127800	Prip	Dragonfly	*Epiophlebia superstes*	Hexapoda	Odonata	Epiophlebiidae
GAYO01130920	Prip 1	Golden-ringed dragonfly	*Cordulegaster boltonii*	Hexapoda	Odonata	Cordulegastridae
GAYO01011149	Prip 2	Golden-ringed dragonfly	*Cordulegaster boltonii*	Hexapoda	Odonata	Cordulegastridae
APVN01148320	Prip 1	Scarce chaser	*Ladona fulva*	Hexapoda	Odonata	Libellulidae
APVN01148314/APVN01148315	Prip 2	Scarce chaser	*Ladona fulva*	Hexapoda	Odonata	Libellulidae
APVN01148320/APVN01148322	Prip 3	Scarce chaser	*Ladona fulva*	Hexapoda	Odonata	Libellulidae
APVN01148322	Prip 4	Scarce chaser	*Ladona fulva*	Hexapoda	Odonata	Libellulidae
GASN01409111	Prip	Firebrat	*Thermobia domestica*	Hexapoda	Zygentoma	Lepismatidae
GAYJ01029739	Prip	Silverfish	*Atelura formicaria*	Hexapoda	Zygentoma	Nicoletiidae
GASO01021146	Prip	Silverfish	*Tricholepidion gertschi*	Hexapoda	Zygentoma	Libellulidae
FN222978	Prip	Bristletail	*Lepismachilis y-signata*	Hexapoda	Archaeognatha	Machilidae
GAUM01020136	Prip	Bristletail	*Machilis hrabei*	Hexapoda	Archaeognatha	Machilidae
GAUG01033311	Prip	Bristletail	*Meinertellus cundinamarcensis*	Hexapoda	Archaeognatha	Meinertellidae
GAYN01007080	Prip 1	Campodea	*Campodea augens*	Hexapoda	Diplura	Campodeidae

GAYN01144154	Prip 2	Campodea	*Campodea augens*	Hexapoda	Diplura	Campodeidae
GAXJ01007613	Prip 1	Two-pronged bristletail	*Occasjapyx japonicus*	Hexapoda	Diplura	Japygidae
GAXJ01010312	Prip 2	Two-pronged bristletail	*Occasjapyx japonicus*	Hexapoda	Diplura	Japygidae
GAXJ01010770	Prip 3	Two-pronged bristletail	*Occasjapyx japonicus*	Hexapoda	Diplura	Japygidae
GAXJ01101258	Prip 4	Two-pronged bristletail	*Occasjapyx japonicus*	Hexapoda	Diplura	Japygidae
GAMM01012413/GAMM01012414	Prip -like1	Springtail	*Orchesella cincta*	Hexapoda	Collembola	Entomobryidae
GAMM01010822	Prip -like2	Springtail	*Orchesella cincta*	Hexapoda	Collembola	Entomobryidae
GAMM01009287	Prip -like3	Springtail	*Orchesella cincta*	Hexapoda	Collembola	Entomobryidae
GAMN01008708	Prip -like1	Springtail	*Folsomia candida*	Hexapoda	Collembola	Isotomidae
GAMN01012115/GAMN01012753	Prip -like2	Springtail	*Folsomia candida*	Hexapoda	Collembola	Isotomidae
sb_005_09I19	Prip -like1	Springtail	*Megaphorura arctica*	Hexapoda	Collembola	Onychiuridae
EW748629	Prip -like2	Springtail	*Megaphorura arctica*	Hexapoda	Collembola	Onychiuridae
GATZ01003502	Prip -like1	Clover springtail	*Sminthurus viridis*	Hexapoda	Collembola	Sminthuridae
GATZ01016023	Prip -like2	Clover springtail	*Sminthurus viridis*	Hexapoda	Collembola	Sminthuridae
GATZ01103627	Prip -like3	Clover springtail	*Sminthurus viridis*	Hexapoda	Collembola	Sminthuridae
GAUE01008782	Prip -like1	Cosmopolitan springtail	*Anurida maritima*	Hexapoda	Collembola	Neanuridae
GAUE01009121	Prip -like2	Cosmopolitan springtail	*Anurida maritima*	Hexapoda	Collembola	Neanuridae
GAUE01052561	Prip -like3	Cosmopolitan springtail	*Anurida maritima*	Hexapoda	Collembola	Neanuridae
GAXI01003818	Prip -like1	Giant springtail	*Tetrodontophora bielanensis*	Hexapoda	Collembola	Onychiuridae
GAXI01003820	Prip -like2	Giant springtail	*Tetrodontophora bielanensis*	Hexapoda	Collembola	Onychiuridae
GAXI01020146	Prip -like3	Giant springtail	*Tetrodontophora bielanensis*	Hexapoda	Collembola	Onychiuridae
GATD01009723	Prip -like1	Springtail	*Pogonognathellus sp.*	Hexapoda	Collembola	Tomoceridae
GATD01010900	Prip -like2	Springtail	*Pogonognathellus sp.*	Hexapoda	Collembola	Tomoceridae
EV474850/EV478034	Prip -like V-paralog	Springtail	*Folsomia candida*	Hexapoda	Collembola	Isotomidae
GATZ01102432	Prip -like I-paralog	Clover springtail	*Sminthurus viridis*	Hexapoda	Collembola	Sminthuridae
GAUE01011057	Prip -like I-paralog	Cosmopolitan springtail	*Anurida maritima*	Hexapoda	Collembola	Neanuridae
GAXI01015403	Prip -like V-paralog	Giant springtail	*Tetrodontophora bielanensis*	Hexapoda	Collembola	Onychiuridae
GAXI01018099	Prip -like I-paralog	Giant springtail	*Tetrodontophora bielanensis*	Hexapoda	Collembola	Onychiuridae
GATD01099700	Prip -like I-paralog	Springtail	*Pogonognathellus sp.*	Hexapoda	Collembola	Tomoceridae
GAXE01021065	Prip -like1	Conehead	*Acerentomon sp.*	Hexapoda	Protura	Acerentomidae
GAXE01011432	Prip -like2	Conehead	*Acerentomon sp.*	Hexapoda	Protura	Acerentomidae
AFFK01018906	Prip -like	Coastal European centipede	*Strigamia maritima*	Chilopoda	Geophilomorpha	Linotaeniidae
AFFK01016279	Prip -like	Coastal European centipede	*Strigamia maritima*	Chilopoda	Geophilomorpha	Linotaeniidae
AFFK01018906	Prip -like	Coastal European centipede	*Strigamia maritima*	Chilopoda	Geophilomorpha	Linotaeniidae
AFFK01022706	Prip -like	Coastal European centipede	*Strigamia maritima*	Chilopoda	Geophilomorpha	Linotaeniidae
AFFK01021957	Prip -like	Coastal European centipede	*Strigamia maritima*	Chilopoda	Geophilomorpha	Linotaeniidae
AFFK01015980	Prip -like	Coastal European centipede	*Strigamia maritima*	Chilopoda	Geophilomorpha	Linotaeniidae
AFFK01023535/AFFK01023534	Prip -like	Coastal European centipede	*Strigamia maritima*	Chilopoda	Geophilomorpha	Linotaeniidae
AFFK01013572	Prip -like	Coastal European centipede	*Strigamia maritima*	Chilopoda	Geophilomorpha	Linotaeniidae
AFFK01013572	Prip -like	Coastal European centipede	*Strigamia maritima*	Chilopoda	Geophilomorpha	Linotaeniidae
AFFK01013573	Prip -like	Coastal European centipede	*Strigamia maritima*	Chilopoda	Geophilomorpha	Linotaeniidae
AFFK01013573	Prip -like	Coastal European centipede	*Strigamia maritima*	Chilopoda	Geophilomorpha	Linotaeniidae
GAFS01003350	Prip -like	Narrow-clawed crayfish	*Pontastacus leptodactylus*	Malacostraca	Decapoda	Astacidae
AET34919	Prip -like	Giant freshwater prawn	*Macrobrachium rosenbergii*	Malacostraca	Decapoda	Palaemonidae
AEI25531	Prip -like	Black tiger shrimp	*Penaeus monodon*	Malacostraca	Decapoda	Penaeidae
FE136953/JP420962/FE081160	Prip -like	Pacific white shrimp	*Litopenaeus vannamei*	Malacostraca	Decapoda	Penaeidae
FD699313	Prip -like	American lobster	*Homarus americanus*	Malacostraca	Decapoda	Nephropidae
JQ970426	Prip -like	Blue crab	*Callinectes sapidus*	Malacostraca	Decapoda	Portunidae
GT562677	Prip -like	Swimming crab	*Portunus trituberculatus*	Malacostraca	Decapoda	Portunidae
GAKD01017822	Prip -like	Amphipod	*Melita plumulosa*	Malacostraca	Amphipoda	Melitidae
JL195955	Prip -like	Remipede	*Speleonectes cf. tulumensis*	Remipedia	Nectiopoda	Speleonectidae
ACO10737	Prip -like	Caligid copepod	*Caligus rogercresseyi*	Maxillopoda	Siphonostomatoida	Caligidae
GO416492	Prip -like	Cod worm	*Lernaeocera branchialis*	Maxillopoda	Siphonostomatoida	Pennellidae
JV192099	Prip -like	Tide pool copepod	*Tigriopus californicus*	Maxillopoda	Harpacticoida	Harpacticidae
EFX74648	Prip -like	Common water flea	*Daphnia pulex*	Branchiopoda	Diplostraca	Daphniidae
GACK01000433	Prip -like	Zebra tick	*Rhipicephalus pulchellus*	Acari	Ixodida	Ixodidae
ADDG01009295/ADDG01028715	Prip -like	Honeybee mite	*Varroa destructor*	Acari	Mesostigmata	Varroidae
XP_003740496	Prip -like	Western predatory mite	*Metaseiulus occidentalis*	Acari	Mesostigmata	Phytoseiidae
JR695181	Prip -like	Two-spotted spider mite	*Tetranychus urticae*	Acari	Acariformes	Tetranychidae
JT045087	Prip -like1	African social eresid spider	*Stegodyphus tentoriicola*	Arachnida	Araneae	Eresidae
GAZR01012136/JT037476	Prip -like1	African social eresid spider	*Stegodyphus mimosarum*	Arachnida	Araneae	Eresidae
AOMJ01121993	Prip -like1	Common house spider	*Parasteatoda tepidariorum*	Arachnida	Araneae	Theridiidae
GAZS01059885	Prip -like1	Brazilian giant whiteknee tarantula	*Acanthoscurria geniculata*	Arachnida	Araneae	Theraphosidae
JAA92966	Prip -like2	American wandering spider	*Cupiennius salei*	Arachnida	Araneae	Theridiidae
GANL01009509	Prip -like2	Black widow	*Latrodectus tredecimguttatus*	Arachnida	Araneae	Theridiidae
GBCS01013355	Prip -like2	Western black widow	*Latrodectus hesperus*	Arachnida	Araneae	Theridiidae
AOMJ01225662/AOMJ01225654	Prip -like2	Common house spider	*Parasteatoda tepidariorum*	Arachnida	Araneae	Theridiidae
DR443206	Prip -like2	São paulo black tarantula	*Acanthoscurria gomesiana*	Arachnida	Araneae	Theraphosidae
GAZS01036184	Prip -like2	Brazilian giant whiteknee tarantula	*Acanthoscurria geniculata*	Arachnida	Araneae	Theraphosidae
AYEL01066910/AYEL01075601	Prip -like1	Chinese scorpion	*Mesobuthus martensii*	Arachnida	Scorpiones	Buthidae
AXZI01136975/AYEL01075601	Prip -like1	Baja California bark scorpion	*Centruroides exilicauda*	Arachnida	Scorpiones	Buthidae
AYEL01052592	Prip -like2	Chinese scorpion	*Mesobuthus martensii*	Arachnida	Scorpiones	Buthidae
AXZI01008908	Prip -like2	Baja California bark scorpion	*Centruroides exilicauda*	Arachnida	Scorpiones	Buthidae
AYEL01088164	Prip -like3	Chinese scorpion	*Mesobuthus martensii*	Arachnida	Scorpiones	Buthidae
AXZI01127083	Prip -like3	Baja California bark scorpion	*Centruroides exilicauda*	Arachnida	Scorpiones	Buthidae
**Hexapoda Entomoglyceroporins**						
FBpp0072014	Eglp 4	Fruit fly	*Drosophila melanogaster*	Hexapoda	Diptera	Drosophilidae
FBpp0196999	Eglp 4	Fruit fly	*Drosophila sechellia*	Hexapoda	Diptera	Drosophilidae
FBpp0223428	Eglp 4	Fruit fly	*Drosophila simulans*	Hexapoda	Diptera	Drosophilidae
FBpp0256556	Eglp 4	Fruit fly	*Drosophila yakuba*	Hexapoda	Diptera	Drosophilidae
FBpp0138556	Eglp 4	Fruit fly	*Drosophila erecta*	Hexapoda	Diptera	Drosophilidae
FBpp0115022	Eglp 4	Fruit fly	*Drosophila ananassae*	Hexapoda	Diptera	Drosophilidae
FBpp0278011	Eglp 4	Fruit fly	*Drosophila pseudoobscura*	Hexapoda	Diptera	Drosophilidae
FBpp0181703	Eglp 4	Fruit fly	*Drosophila persimilis*	Hexapoda	Diptera	Drosophilidae
FBpp0236772	Eglp 4	Fruit fly	*Drosophila virilis*	Hexapoda	Diptera	Drosophilidae
FBpp0169721	Eglp 4	Fruit fly	*Drosophila mojavensis*	Hexapoda	Diptera	Drosophilidae
FBpp0155125	Eglp 4	Fruit fly	*Drosophila grimshawi*	Hexapoda	Diptera	Drosophilidae
FBpp0252317	Eglp 4	Fruit fly	*Drosophila willistoni*	Hexapoda	Diptera	Drosophilidae
EZ127051	Eglp 4	Apple maggot	*Rhagoletis pomonella*	Hexapoda	Diptera	Tephritidae
GAKB01002522	Eglp 4a	Olive fruit fly	*Bactrocera oleae*	Hexapoda	Diptera	Tephritidae
GAKB01004245	Eglp 4b	Olive fruit fly	*Bactrocera oleae*	Hexapoda	Diptera	Tephritidae
GAKB01003899	Eglp 4c	Olive fruit fly	*Bactrocera oleae*	Hexapoda	Diptera	Tephritidae
ACT34033	Eglp 4	Goldenrod gall fly	*Eurosta solidaginis*	Hexapoda	Diptera	Tephritidae
XM_004522562	Eglp 4	Mediterranean fruit fly	*Ceratitis capitata*	Hexapoda	Diptera	Tephritidae
JG419371	Eglp 4	Australian sheep blowfly	*Lucilia cuprina*	Hexapoda	Diptera	Calliphoridae
FG295968/FG298919	Eglp 4	Primary screw-worm	*Cochliomyia hominivorax*	Hexapoda	Diptera	Calliphoridae
EZ597305	Eglp 4	Flesh fly	*Sarcophaga crassipalpis*	Hexapoda	Diptera	Sarcophagidae
FD460504	Eglp 4	Horn fly	*Haematobia irritans irritans*	Hexapoda	Diptera	Muscidae

AQPM01070455/XP_005183005	Eglp 4a	House fly	*Musca domestica*	Hexapoda	Diptera	Muscidae
AQPM01070459	Eglp 4b	House fly	*Musca domestica*	Hexapoda	Diptera	Muscidae
FBpp0072015	Eglp 3	Fruit fly	*Drosophila melanogaster*	Hexapoda	Diptera	Drosophilidae
FBpp0197000	Eglp 3	Fruit fly	*Drosophila sechellia*	Hexapoda	Diptera	Drosophilidae
FBpp0223429	Eglp 3	Fruit fly	*Drosophila simulans*	Hexapoda	Diptera	Drosophilidae
FBpp0256558	Eglp 3	Fruit fly	*Drosophila yakuba*	Hexapoda	Diptera	Drosophilidae
FBpp0138557	Eglp 3	Fruit fly	*Drosophila erecta*	Hexapoda	Diptera	Drosophilidae
FBpp0115023	Eglp 3	Fruit fly	*Drosophila ananassae*	Hexapoda	Diptera	Drosophilidae
FBpp0278012	Eglp 3	Fruit fly	*Drosophila pseudoobscura*	Hexapoda	Diptera	Drosophilidae
FBpp0181704	Eglp 3	Fruit fly	*Drosophila persimilis*	Hexapoda	Diptera	Drosophilidae
FBpp0169722	Eglp 3	Fruit fly	*Drosophila mojavensis*	Hexapoda	Diptera	Drosophilidae
FBpp0155126	Eglp 3	Fruit fly	*Drosophila grimshawi*	Hexapoda	Diptera	Drosophilidae
FBpp0252320	Eglp 3	Fruit fly	*Drosophila willistoni*	Hexapoda	Diptera	Drosophilidae
FBpp0072016	Eglp 2	Fruit fly	*Drosophila melanogaster*	Hexapoda	Diptera	Drosophilidae
FBpp0197001	Eglp 2	Fruit fly	*Drosophila sechellia*	Hexapoda	Diptera	Drosophilidae
FBpp0223430	Eglp 2	Fruit fly	*Drosophila simulans*	Hexapoda	Diptera	Drosophilidae
FBpp0256559	Eglp 2	Fruit fly	*Drosophila yakuba*	Hexapoda	Diptera	Drosophilidae
FBpp0138558	Eglp 2	Fruit fly	*Drosophila erecta*	Hexapoda	Diptera	Drosophilidae
FBpp0115024	Eglp 2	Fruit fly	*Drosophila ananassae*	Hexapoda	Diptera	Drosophilidae
FBpp0278013	Eglp 2	Fruit fly	*Drosophila pseudoobscura*	Hexapoda	Diptera	Drosophilidae
FBpp0181705	Eglp 2	Fruit fly	*Drosophila persimilis*	Hexapoda	Diptera	Drosophilidae
FBpp0236774	Eglp 2	Fruit fly	*Drosophila virilis*	Hexapoda	Diptera	Drosophilidae
FBpp0155127	Eglp 2	Fruit fly	*Drosophila grimshawi*	Hexapoda	Diptera	Drosophilidae
FBpp0252321	Eglp 2	Fruit fly	*Drosophila willistoni*	Hexapoda	Diptera	Drosophilidae
AFP49896	Eglp 2a	Tsetse fly	*Glossina morsitans morsitans*	Hexapoda	Diptera	Glossinidae
ADD19418	Eglp 2b	Tsetse fly	*Glossina morsitans morsitans*	Hexapoda	Diptera	Glossinidae
AFP49898	Eglp 2c	Tsetse fly	*Glossina morsitans morsitans*	Hexapoda	Diptera	Glossinidae
ADD18960	Eglp 2d	Tsetse fly	*Glossina morsitans morsitans*	Hexapoda	Diptera	Glossinidae
AAEL005008/XP_001650169	Eglp 2	Yellow fever mosquito	*Aedes aegypti*	Hexapoda	Diptera	Culicidae
JO854615	Eglp 2	Asian tiger mosquito	*Aedes albopictus*	Hexapoda	Diptera	Culicidae
CPIJ009225	Eglp 2	Southern house mosquito	*Culex quinquefasciatus*	Hexapoda	Diptera	Culicidae
AGAP010325	Eglp 2	African malaria mosquito	*Anopheles gambiae*	Hexapoda	Diptera	Culicidae
EZ975669	Eglp 2	African malaria mosquito	*Anopheles funestus*	Hexapoda	Diptera	Culicidae
GAFE01000152	Eglp 2	Indonesian malaria mosquito	*Anopheles sinensis*	Hexapoda	Diptera	Culicidae
GAMD01001135	Eglp 2	South American malaria mosquito	*Anopheles aquasalis*	Hexapoda	Diptera	Culicidae
BAF62091	Eglp 2	Sleeping chironomid	*Polypedilum vanderplanki*	Hexapoda	Diptera	Chironomidae
GAAK01006050	Eglp 2	Antarctic flightless midge	*Belgica antarctica*	Hexapoda	Diptera	Chironomidae
KA181130	Eglp 2	Harlequin fly	*Chironomus riparius*	Hexapoda	Diptera	Chironomidae
AEGA01025736	Eglp 2	Hessian fly	*Mayetiola destructor*	Hexapoda	Diptera	Cecidomyiidae
EZ406461	Eglp 2	Hessian fly	*Mayetiola destructor*	Hexapoda	Diptera	Cecidomyiidae
JP552203	Eglp 2	Sand fly	*Phlebotomus papatasi*	Hexapoda	Diptera	Psychodidae
FBpp0071980	Eglp 1	Fruit fly	*Drosophila melanogaster*	Hexapoda	Diptera	Drosophilidae
FBpp0259319	Eglp 1	Fruit fly	*Drosophila yakuba*	Hexapoda	Diptera	Drosophilidae
FBpp0141417	Eglp 1	Fruit fly	*Drosophila erecta*	Hexapoda	Diptera	Drosophilidae
FBpp0116260	Eglp 1	Fruit fly	*Drosophila ananassae*	Hexapoda	Diptera	Drosophilidae
FBpp0281368	Eglp 1	Fruit fly	*Drosophila pseudoobscura*	Hexapoda	Diptera	Drosophilidae
FBpp0185590	Eglp 1	Fruit fly	*Drosophila persimilis*	Hexapoda	Diptera	Drosophilidae
FBpp0234409	Eglp 1a	Fruit fly	*Drosophila virilis*	Hexapoda	Diptera	Drosophilidae
FBpp0168243	Eglp 1a	Fruit fly	*Drosophila mojavensis*	Hexapoda	Diptera	Drosophilidae
FBpp0154569	Eglp 1a	Fruit fly	*Drosophila grimshawi*	Hexapoda	Diptera	Drosophilidae
FBpp0252206	Eglp 1	Fruit fly	*Drosophila willistoni*	Hexapoda	Diptera	Drosophilidae
AAEL005001/XP_001650168	Eglp 1	Yellow fever mosquito	*Aedes aegypti*	Hexapoda	Diptera	Culicidae
JO889430	Eglp 1	Asian tiger mosquito	*Aedes albopictus*	Hexapoda	Diptera	Culicidae
CPIJ009224	Eglp 1	Southern house mosquito	*Culex quinquefasciatus*	Hexapoda	Diptera	Culicidae
AGAP010326	Eglp 1	African malaria mosquito	*Anopheles gambiae*	Hexapoda	Diptera	Culicidae
JP541300	Eglp 1	Sand fly	*Phlebotomus papatasi*	Hexapoda	Diptera	Psychodidae
GAAK01006051	Eglp 1a	Antarctic flightless midge	*Belgica antarctica*	Hexapoda	Diptera	Chironomidae
GAAK01006051	Eglp 1b	Antarctic flightless midge	*Belgica antarctica*	Hexapoda	Diptera	Chironomidae
AK383767	Eglp 1	Domestic silkworm	*Bombyx mori*	Hexapoda	Lepidoptera	Bombycidae
AB245966	Eglp 2	Domestic silkworm	*Bombyx mori*	Hexapoda	Lepidoptera	Bombycidae
CAEZ01006548	Eglp 1	Postman butterfly	*Heliconius melpomene melpomene*	Hexapoda	Lepidoptera	Nymphalidae
CAEZ01001062	Eglp 2	Postman butterfly	*Heliconius melpomene melpomene*	Hexapoda	Lepidoptera	Nymphalidae
CAEZ01001062	Eglp 3	Postman butterfly	*Heliconius melpomene melpomene*	Hexapoda	Lepidoptera	Nymphalidae
CAEZ01001062	Eglp 4	Postman butterfly	*Heliconius melpomene melpomene*	Hexapoda	Lepidoptera	Nymphalidae
EHJ72288	Eglp 1	Monarch butterfly	*Danaus plexippus*	Hexapoda	Lepidoptera	Nymphalidae
EHJ65755	Eglp 2	Monarch butterfly	*Danaus plexippus*	Hexapoda	Lepidoptera	Nymphalidae
EHJ78266	Eglp 3	Monarch butterfly	*Danaus plexippus*	Hexapoda	Lepidoptera	Nymphalidae
EHJ72289	Eglp 4	Monarch butterfly	*Danaus plexippus*	Hexapoda	Lepidoptera	Nymphalidae
EB823882	Eglp 2	Indianmeal moth	*Plodia interpunctella*	Hexapoda	Lepidoptera	Pyralidae
JP717914	Eglp 2	Asian Swallowtail	*Papilio xuthus*	Hexapoda	Lepidoptera	Papilionidae
GAJS01025088	Eglp 1	striped riceborer	*Chilo suppressalis*	Hexapoda	Lepidoptera	Crambidae
GAJS01070620	Eglp 2	striped riceborer	*Chilo suppressalis*	Hexapoda	Lepidoptera	Crambidae
JP612965	Eglp 2	Propertius duskywing	*Erynnis propertius*	Hexapoda	Lepidoptera	Hesperiidae
JO814913	Eglp 1	Tobacco hornworm	*Manduca sexta*	Hexapoda	Lepidoptera	Sphingidae
BAH47555	Eglp 1	Oriental fruit moth	*Grapholita molesta*	Hexapoda	Lepidoptera	Tortricidae
TC014278	Eglp 4	Red flour Beetle	*Tribolium castaneum*	Hexapoda	Coleoptera	Tenebrionidae
TC014279	Eglp 3	Red flour Beetle	*Tribolium castaneum*	Hexapoda	Coleoptera	Tenebrionidae
TC014280	Eglp 2	Red flour Beetle	*Tribolium castaneum*	Hexapoda	Coleoptera	Tenebrionidae
TC014281	Eglp 1	Red flour Beetle	*Tribolium castaneum*	Hexapoda	Coleoptera	Tenebrionidae
GAFI01017518	Eglp 1	Southern pine beetle	*Dendroctonus frontalis*	Hexapoda	Coleoptera	Curculionidae
GACR01006542	Eglp 1	European spruce bark beetle	*Ips typographus*	Hexapoda	Coleoptera	Curculionidae
AEE61512	Eglp 1	Mountain pine weevil	*Dendroctonus ponderosae*	Hexapoda	Coleoptera	Curculionidae
JU408487	Eglp 1	Salt marsh beetle	*Pogonus chalceus*	Hexapoda	Coleoptera	Carabidae
GAXW01009218	Eglp	Antlion	*Euroleon nostras*	Hexapoda	Neoptera	Myrmeleontidae
GAVV01181849	Eglp 1	Green lacewing	*Pseudomallada prasinus*	Hexapoda	Neoptera	Chrysopidae
GAVV01166415	Eglp 2	Green lacewing	*Pseudomallada prasinus*	Hexapoda	Neoptera	Chrysopidae
XP_003403182	Eglp 3	Buff-tailed bumblebee	*Bombus terrestris*	Hexapoda	Hymenoptera	Apidae
XP_003486426	Eglp 3	Common eastern bumble bee	*Bombus impatiens*	Hexapoda	Hymenoptera	Apidae
XP_624194	Eglp 3	Honey bee	*Apis mellifera*	Hexapoda	Hymenoptera	Apidae
GAGH01067111	Eglp 3	Mason bee	*Osmia cornuta*	Hexapoda	Hymenoptera	Megachilidae
XP_003702537	Eglp 3	Alfalfa leafcutting bee	*Megachile rotundata*	Hexapoda	Hymenoptera	Megachilidae
EGI59562	Eglp 3	Panamanian leafcutter ant	*Acromyrmex echinatior*	Hexapoda	Hymenoptera	Formicidae
EFN68166	Eglp 3	Florida carpenter ant	*Camponotus floridanus*	Hexapoda	Hymenoptera	Formicidae
EFN76752	Eglp 3	Jerdon's jumping ant	*Harpegnathos saltator*	Hexapoda	Hymenoptera	Formicidae
ACEP_00012182	Eglp 3	Leafcutter ant	*Atta cephalotes*	Hexapoda	Hymenoptera	Formicidae
XP_001601253	Eglp 3	Jewel wasp	*Nasonia vitripennis*	Hexapoda	Hymenoptera	Pteromalidae
ADAO01239456/ADAO01239457/ADAO01239458	Eglp 3	Jewel wasp	*Nasonia giraulti*	Hexapoda	Hymenoptera	Pteromalidae
XP_003403164	Eglp 2	Buff-tailed bumblebee	*Bombus terrestris*	Hexapoda	Hymenoptera	Apidae
XP_003486425	Eglp 2	Common eastern bumble bee	*Bombus impatiens*	Hexapoda	Hymenoptera	Apidae

XP_001121899	Eglp 2	Honey bee	*Apis mellifera*	Hexapoda	Hymenoptera	Apidae
XP_003702536	Eglp 2	Alfalfa leafcutting bee	*Megachile rotundata*	Hexapoda	Hymenoptera	Megachilidae
EGI59563	Eglp 2	Panamanian leafcutter ant	*Acromyrmex echinatior*	Hexapoda	Hymenoptera	Formicidae
EFZ21244	Eglp 2	Red fire ant	*Solenopsis invicta*	Hexapoda	Hymenoptera	Formicidae
ACEP_00012181	Eglp 2	Leafcutter ant	*Atta cephalotes*	Hexapoda	Hymenoptera	Formicidae
XP_001601231	Eglp 2	Jewel wasp	*Nasonia vitripennis*	Hexapoda	Hymenoptera	Pteromalidae
ADAO01239444	Eglp 2	Jewel wasp	*Nasonia giraulti*	Hexapoda	Hymenoptera	Pteromalidae
XP_003393900	Eglp 1	Buff-tailed bumblebee	*Bombus terrestris*	Hexapoda	Hymenoptera	Apidae
XP_003484555	Eglp 1	Common eastern bumble bee	*Bombus impatiens*	Hexapoda	Hymenoptera	Apidae
XP_001121043	Eglp 1	Honey bee	*Apis mellifera*	Hexapoda	Hymenoptera	Apidae
XP_003700908	Eglp 1	Alfalfa leafcutting bee	*Megachile rotundata*	Hexapoda	Hymenoptera	Megachilidae
EGI63170	Eglp 1	Panamanian leafcutter ant	*Acromyrmex echinatior*	Hexapoda	Hymenoptera	Formicidae
EFN67363	Eglp 1	Florida carpenter ant	*Camponotus floridanus*	Hexapoda	Hymenoptera	Formicidae
XP_001603421	Eglp 1	Jewel wasp	*Nasonia vitripennis*	Hexapoda	Hymenoptera	Pteromalidae
ADAO01181999	Eglp 1	Jewel wasp	*Nasonia giraulti*	Hexapoda	Hymenoptera	Pteromalidae
PHUM474700	Eglp 2	Human body louse	*Pediculus humanus corporis*	Hexapoda	Phthiraptera	Pediculidae
PHUM369010	Eglp 1	Human body louse	*Pediculus humanus corporis*	Hexapoda	Phthiraptera	Pediculidae
GAWR01092467	Eglp 1	Poultry shaft louse	*Menopon gallinae*	Hexapoda	Phthiraptera	Menoponidae
GAWR01093370	Eglp 2	Poultry shaft louse	*Menopon gallinae*	Hexapoda	Phthiraptera	Menoponidae
GAWR01009252	Eglp 3	Poultry shaft louse	*Menopon gallinae*	Hexapoda	Phthiraptera	Menoponidae
GAWR01011369	Eglp 4	Poultry shaft louse	*Menopon gallinae*	Hexapoda	Phthiraptera	Menoponidae
GAYV01019679	Eglp 1	Booklice	*Liposcelis bostrychophila*	Hexapoda	Psocoptera	Liposcelidae
GAYV01019146	Eglp 2	Booklice	*Liposcelis bostrychophila*	Hexapoda	Psocoptera	Liposcelidae
GAPT01001679	Eglp 1	Booklice	*Ectopsocus briggsi*	Hexapoda	Psocoptera	Ectopsocidae
GAPT01090202	Eglp 2	Booklice	*Ectopsocus briggsi*	Hexapoda	Psocoptera	Ectopsocidae
GAXD01000016	Eglp	Western flower thrips	*Frankliniella occidentalis*	Hexapoda	Thysanoptera	Thripidae
GAJY01002108	Eglp	Cowpea flower thrips	*Megalurothrips sjostedti*	Hexapoda	Thysanoptera	Thripidae
CAC13959/AEV57515/AEV57516	Eglp	Assassin bug	*Rhodnius prolixus*	Hexapoda	Hemiptera	Reduviidae
KF048098	Eglp 1	Lygus bug	*Lygus hesperus*	Hexapoda	Hemiptera	Miridae
KF048093	Eglp 2A	Lygus bug	*Lygus hesperus*	Hexapoda	Hemiptera	Miridae
KF048094	Eglp 2B	Lygus bug	*Lygus hesperus*	Hexapoda	Hemiptera	Miridae
KF048095	Eglp 2C	Lygus bug	*Lygus hesperus*	Hexapoda	Hemiptera	Miridae
KF048096	Eglp 2D	Lygus bug	*Lygus hesperus*	Hexapoda	Hemiptera	Miridae
KF048097	Eglp 2E	Lygus bug	*Lygus hesperus*	Hexapoda	Hemiptera	Miridae
XP_001952198	Eglp	Pea aphid	*Acyrthosiphon pisum*	Hexapoda	Hemiptera	Aphididae
FO032683	Eglp	Shallot aphid	*Myzus ascalonicus*	Hexapoda	Hemiptera	Aphididae
GAOM01002305	Eglp	Potato aphid	*Macrosiphum euphorbiae*	Hexapoda	Hemiptera	Aphididae
GW522658	Eglp	Cotton aphid	*Aphis gossypii*	Hexapoda	Hemiptera	Aphididae
AHB86603	Eglp	Potato psyllid	*Bactericera cockerelli*	Hexapoda	Hemiptera	Triozidae
AHB86602	Eglp	Potato psyllid	*Bactericera cockerelli*	Hexapoda	Hemiptera	Triozidae
AHB86601	Eglp	Potato psyllid	*Bactericera cockerelli*	Hexapoda	Hemiptera	Triozidae
XP_008487274	Eglp	Asian citrus psyllid	*Diaphorina citri*	Hexapoda	Hemiptera	Psyllidae
GACJ01000325	Eglp	Asian citrus psyllid	*Diaphorina citri*	Hexapoda	Hemiptera	Psyllidae
HP650016	Eglp 1	Sweet potato whitefly	*Bemisia tabaci*	Hexapoda	Hemiptera	Aleyrodidae
HP660290	Eglp 2	Sweet potato whitefly	*Bemisia tabaci*	Hexapoda	Hemiptera	Aleyrodidae
GAJV01000737	Eglp	Cowpea pod-sucking bug	*Anoplocnemis curvipes*	Hexapoda	Hemiptera	Coreidae
GAJX01000223	Eglp	African pod bug	*Clavigralla tomentosicollis*	Hexapoda	Hemiptera	Coreidae
BAN21211	Eglp	Bean bug	*Riptortus pedestris*	Hexapoda	Hemiptera	Coreidae
GBID01002499	Eglp	German cockroach	*Blattella germanica*	Hexapoda	Blattodea	Ectobiidae
FG131060/GAWS01002503	Eglp	American cockroach	*Periplaneta americana*	Hexapoda	Blattodea	Blattidae
GAYD01331798	Eglp	Blaberus cockroach	*Blaberus atropos*	Hexapoda	Blattodea	Blaberidae
GAZN01202855	Eglp 1	Brown hooded cockroach	*Cryptocercus wrighti*	Hexapoda	Blattodea	Cryptocercidae
GAZN01196580	Eglp 2	Brown hooded cockroach	*Cryptocercus wrighti*	Hexapoda	Blattodea	Cryptocercidae
FX376451	Eglp	Wood-eating higher termite	*Nasutitermes takasagoensis*	Hexapoda	Isoptera	Termitidae
AUST01030857	Eglp	Nevada dampwood termite	*Zootermopsis nevadensis*	Hexapoda	Isoptera	Termopsidae
GASE01009002	Eglp	Cuban subterranean termite	*Prorhinotermes simplex*	Hexapoda	Isoptera	Rhinotermitidae
FL640174/FL639044	Eglp	Eastern subterranean termite	*Reticulitermes flavipes*	Hexapoda	Isoptera	Rhinotermitidae
GATB01335234	Eglp	Metallyticid mantis	*Metallyticus splendidus*	Hexapoda	Mantodea	Metallyticidae
GASW01223743	Eglp	Praying mantis	*Mantis religiosa*	Hexapoda	Mantodea	Mantidae
GAYA01013920	Eglp	Zoraptid	*Zorotypus caudelli*	Hexapoda	Orthoptera	Zorotypidae
GAAX01017048	Eglp	European earwig	*Forficula auricularia*	Hexapoda	Dermaptera	Forficulidae
GAYL01128297	Eglp	Stonefly	*Cosmioperla kuna*	Hexapoda	Plecoptera	Eustheniidae
GAUF01000114	Eglp	Leuctra	*Leuctra sp.*	Hexapoda	Plecoptera	Leuctridae
GATV01154381	Eglp	Stonefly	*Perla marginata*	Hexapoda	Plecoptera	Perlidae
GAWU01009413	Eglp	Webspinner	*Aposthonia japonica*	Hexapoda	Embioptera	Oligotomidae
GAWG01091169	Eglp	Giant prickly stick insect	*Extatosoma tiaratum*	Hexapoda	Phasmatodea	Phasmatidae
GAWE01075831	Eglp	Vietnamese walking stick insect	*Ramulus artemis*	Hexapoda	Phasmatodea	Phasmatidae
GAWD01039255	Eglp	Vietnamese walking stick	*Medauroidea extradentata*	Hexapoda	Phasmatodea	Phasmatidae
GAWF01050690	Eglp	Pink winged stick insect	*Sipyloidea sipylus*	Hexapoda	Phasmatodea	Diapheromeridae
GAWC01056573	Eglp	Thorny stick insect	*Aretaon asperrimus*	Hexapoda	Phasmatodea	Heteropterygidae
GAXB01152638	Eglp 1	Heelwalker	*Tanzaniophasma sp.*	Hexapoda	Mantophasmatodea	Tanzaniophasmatidae
GAXB01151664	Eglp 2	Heelwalker	*Tanzaniophasma sp.*	Hexapoda	Mantophasmatodea	Tanzaniophasmatidae
AVCP010359984	Eglp	Migratory locust	*Locusta migratoria*	Hexapoda	Orthoptera	Acrididae
GAZT01001309	Eglp	False stick insect	*Prosarthria teretrirostris*	Hexapoda	Orthoptera	Proscopiidae
GBHB01072046	Eglp	Oceanic field cricket	*Teleogryllus commodus*	Hexapoda	Orthoptera	Gryllidae
GAIZ01015418	Eglp	Sand field cricket	*Gryllus firmus*	Hexapoda	Orthoptera	Gryllidae
GASQ01128632	Eglp	Slender Groundhopper	*Tetrix subulata*	Hexapoda	Orthoptera	Tetrigidae
GAUZ01005949	Eglp	Stripe-winged grasshopper	*Stenobothrus lineatus*	Hexapoda	Orthoptera	Acrididae
GATU01013392	Eglp 1	Blue-winged olive	*Baetis sp.*	Hexapoda	Ephemeroptera	Baetidae
GATU01003749	Eglp 2	Blue-winged olive	*Baetis sp.*	Hexapoda	Ephemeroptera	Baetidae
GAXA01012395	Eglp	Mahogany Dun	*Isonychia bicolor*	Hexapoda	Ephemeroptera	Isonychiidae
GAZG01100517	Eglp	Mayfly	*Eurylophella sp.*	Hexapoda	Ephemeroptera	Ephemerellidae
AYNC01028369	Eglp	Green drake	*Ephemera danica*	Hexapoda	Ephemeroptera	Ephemeridae
GAYM01007100	Eglp	Banded damoiselle	*Calopteryx splendens*	Hexapoda	Odonata	Calopterygidae
GAVW01007834	Eglp	Dragonfly	*Epiophlebia superstes*	Hexapoda	Odonata	Epiophlebiidae
GAYO01001919	Eglp	Golden-ringed dragonfly	*Cordulegaster boltonii*	Hexapoda	Odonata	Cordulegastridae
APVN01045462	Eglp	Scarce chaser	*Ladona fulva*	Hexapoda	Odonata	Libellulidae
GASN01409637	Eglp	Firebrat	*Thermobia domestica*	Hexapoda	Zygentoma	Lepismatidae
GAYJ01034252	Eglp	Silverfish	*Atelura formicaria*	Hexapoda	Zygentoma	Nicoletiidae
GAUM01182693	Eglp	Bristletail	*Machilis hrabei*	Hexapoda	Archaeognatha	Machilidae
GAUG01034808	Eglp	Bristletail	*Meinertellus cundinamarcensis*	Hexapoda	Archaeognatha	Meinertellidae
GAUM01024706	Eglp -like	Bristletail	*Machilis hrabei*	Hexapoda	Archaeognatha	Machilidae
GAUG01247769	Eglp -like	Bristletail	*Meinertellus cundinamarcensis*	Hexapoda	Archaeognatha	Meinertellidae
GAXE01013530	Eglp -like I-paralog	Conehead	*Acerentomon sp.*	Hexapoda	Protura	Acerentomidae
GAXE01015084	Eglp -like S-paralog	Conehead	*Acerentomon sp.*	Hexapoda	Protura	Acerentomidae

Other Protostomia Aqp4 orthologs

AEP14559	Aqp 4L(5)	Water bear	*Milnesium tardigradum*	Tardigrada	Apochela	Milnesiidae
AM916089	Aqp 4L	Rotifer	*Brachionus plicatilis*	Rotifera	Ploimida	Brachionidae

GACQ01006974	Aqp 4L	Rotifer	*Brachionus calyciflorus*	Rotifera	Ploimida	Brachionidae
GACL01020446	Aqp 4L	Rotifer	*Brachionus calyciflorus*	Rotifera	Ploimida	Brachionidae
CAWI020040728	Aqp 4L	Rotifer	*Adineta vaga*	Rotifera	Adinetida	Adinetidae
CAWI020041112	Aqp 4L	Rotifer	*Adineta vaga*	Rotifera	Adinetida	Adinetidae
EKC24616	Aqp 4L	Pacific oyster	*Crassostrea gigas*	Mollusca	Ostreoida	Ostreidae
EKC31493	Aqp 4L	Pacific oyster	*Crassostrea gigas*	Mollusca	Ostreoida	Ostreidae
EKC32885	Aqp 4L	Pacific oyster	*Crassostrea gigas*	Mollusca	Ostreoida	Ostreidae
EKC18308	Aqp 4L	Pacific oyster	*Crassostrea gigas*	Mollusca	Ostreoida	Ostreidae
LotgiP170145	Aqp 4L	Owl limpet	*Lottia gigantea*	Mollusca		Lottiidae
LotgiP145722	Aqp 4L	Owl limpet	*Lottia gigantea*	Mollusca		Lottiidae
LotgiP162143	Aqp 4L	Owl limpet	*Lottia gigantea*	Mollusca		Lottiidae
LotgiP185787	Aqp 4L	Owl limpet	*Lottia gigantea*	Mollusca		Lottiidae
AMQO01001063/ESO82755	Aqp 4L	Owl limpet	*Lottia gigantea*	Mollusca		Lottiidae
LotgiP82572	Aqp 4L	Owl limpet	*Lottia gigantea*	Mollusca		Lottiidae
JI270267	Aqp 4L	Asiatic hard clam	*Meretrix meretrix*	Mollusca	Veneroida	Veneridae
GAEH01002204	Aqp 4L	Sea water clam	*Ruditapes philippinarum*	Mollusca	Veneroida	Veneridae
GALB01005414	Aqp 4L	Chinese razor clam	*Sinonovacula constricta*	Mollusca	Veneroida	Solecurtidae
XP_005091351	Aqp 4L	California sea hare	*Aplysia californica*	Mollusca	Euopisthobranchia	Aplysiidae
JR447896	Aqp 4L	Common octopus	*Octopus vulgaris*	Mollusca	Octopoda	Octopodidae
Smp_128110/CCD77873	Aqp 4L	Flatworm	*Schistosoma mansoni*	Platyhelminthes	Strigeidida	Schistosomatidae
CAX48992	Aqp 4L	Humus earthworm	*Lumbricus rubellus*	Annelida	Haplotaxida	Lumbricidae
CAX48991	Aqp 4L	Humus earthworm	*Lumbricus rubellus*	Annelida	Haplotaxida	Lumbricidae
CAX48970	Aqp 4L	Tiger-like worm	*Eisenia andrei*	Annelida	Haplotaxida	Lumbricidae
CapteP176137	Aqp 4L	Segmented worm	*Capitella teleta*	Annelida	Capitellida	Capitellidae
CapteP167790	Aqp 4L	Segmented worm	*Capitella teleta*	Annelida	Capitellida	Capitellidae
CapteP25505	Aqp 4L	Segmented worm	*Capitella teleta*	Annelida	Capitellida	Capitellidae
CapteP120819	Aqp 4L	Segmented worm	*Capitella teleta*	Annelida	Capitellida	Capitellidae
CapteP115384	Aqp 4L	Segmented worm	*Capitella teleta*	Annelida	Capitellida	Capitellidae
CapteP172599	Aqp 4L	Segmented worm	*Capitella teleta*	Annelida	Capitellida	Capitellidae
CapteP26353	Aqp 4L	Segmented worm	*Capitella teleta*	Annelida	Capitellida	Capitellidae
CapteP148029	Aqp 4L	Segmented worm	*Capitella teleta*	Annelida	Capitellida	Capitellidae
CapteP40279	Aqp 4L	Segmented worm	*Capitella teleta*	Annelida	Capitellida	Capitellidae
CapteP37043	Aqp 4L	Segmented worm	*Capitella teleta*	Annelida	Capitellida	Capitellidae
CapteP180401	Aqp 4L	Segmented worm	*Capitella teleta*	Annelida	Capitellida	Capitellidae
CapteP46197	Aqp 4L	Segmented worm	*Capitella teleta*	Annelida	Capitellida	Capitellidae
CapteP45698	Aqp 4L	Segmented worm	*Capitella teleta*	Annelida	Capitellida	Capitellidae
CapteP21078	Aqp 4L	Segmented worm	*Capitella teleta*	Annelida	Capitellida	Capitellidae
CapteP197140	Aqp 4L	Segmented worm	*Capitella teleta*	Annelida	Capitellida	Capitellidae
CapteP142373	Aqp 4L	Segmented worm	*Capitella teleta*	Annelida	Capitellida	Capitellidae
HelroP185353	Aqp 4L	Freshwater leech	*Helobdella robusta*	Annelida	Rhynchobdellida	Glossiphoniidae
HelroP185352	Aqp 4L	Freshwater leech	*Helobdella robusta*	Annelida	Rhynchobdellida	Glossiphoniidae
HelroP168509	Aqp 4L	Freshwater leech	*Helobdella robusta*	Annelida	Rhynchobdellida	Glossiphoniidae
HelroP68110	Aqp 4L	Freshwater leech	*Helobdella robusta*	Annelida	Rhynchobdellida	Glossiphoniidae
HelroP95007	Aqp 4L	Freshwater leech	*Helobdella robusta*	Annelida	Rhynchobdellida	Glossiphoniidae
HelroP193953	Aqp 4L	Freshwater leech	*Helobdella robusta*	Annelida	Rhynchobdellida	Glossiphoniidae
HelroP176474	Aqp 4L	Freshwater leech	*Helobdella robusta*	Annelida	Rhynchobdellida	Glossiphoniidae
HelroP113524	Aqp 4L	Freshwater leech	*Helobdella robusta*	Annelida	Rhynchobdellida	Glossiphoniidae
HelroP185178	Aqp 4L	Freshwater leech	*Helobdella robusta*	Annelida	Rhynchobdellida	Glossiphoniidae
HelroP165272	Aqp 4L	Freshwater leech	*Helobdella robusta*	Annelida	Rhynchobdellida	Glossiphoniidae
HelroP154942	Aqp 4L	Freshwater leech	*Helobdella robusta*	Annelida	Rhynchobdellida	Glossiphoniidae
HelroP185503	Aqp 4L	Freshwater leech	*Helobdella robusta*	Annelida	Rhynchobdellida	Glossiphoniidae

Deuterostomia Aqp8 orthologs

ENSP00000219660	AQP 8	Human	*Homo sapiens*	Euarchontoglires	Primates	Hominidae
ENSPTRP00000013506	AQP 8	Chimpanzee	*Pan troglodytes*	Euarchontoglires	Primates	Hominidae
ENSGGOP00000010766	AQP 8	Western lowland gorilla	*Gorilla gorilla gorilla*	Euarchontoglires	Primates	Hominidae
ENSPPYP00000008147	AQP 8	Sumatran orangutan	*Pongo abelii*	Euarchontoglires	Primates	Hominidae
ENSMMUP00000013727	AQP 8	Rhesus macaque	*Macaca mulatta*	Euarchontoglires	Primates	Cercopithecidae
ENSTSYP00000008941	AQP 8	Philippine tarsier	*Tarsius syrichta*	Euarchontoglires	Primates	Tarsiidae
ENSMICP00000014811	AQP 8	Gray mouse lemur	*Microcebus murinus*	Euarchontoglires	Primates	Cheirogaleidae
XP_003795792/XP_003795895/<br>ENSOGAP00000008315	AQP 8	Small-eared galago/Bushbaby	*Otolemur garnettii*	Euarchontoglires	Primates	Galagidae
ENSMUSP00000033023	AQP 8	Mouse	*Mus musculus*	Euarchontoglires	Rodentia	Muridae
ENSRNOP00000019939	AQP 8	Norway rat	*Rattus norvegicus*	Euarchontoglires	Rodentia	Muridae
ENSDORP00000001917	AQP 8	Ord's kangaroo rat	*Dipodomys ordii*	Euarchontoglires	Rodentia	Heteromyidae
AAKN02007697/XP_003478257/<br>ENSCPOP00000004692	AQP 8	Domestic guinea pig	*Cavia porcellus*	Euarchontoglires	Rodentia	Caviidae
ENSSTOP00000000373	AQP 8	Thirteen-lined ground squirrel	*Ictidomys tridecemlineatus*	Euarchontoglires	Rodentia	Sciuridae
ENSOCUP00000001602	AQP 8	Rabbit	*Oryctolagus cuniculus*	Euarchontoglires	Lagomorpha	Leporidae
ENSEEUP00000008056	AQP 8	Western European hedgehog	*Erinaceus europaeus*	Laurasiatheria	Insectivora	Erinaceinae
AALT02037545/ENSSART00000003839	AQP 8	Common shrew	*Sorex araneus*	Laurasiatheria	Insectivora	Soricidae
ENSPVAP00000007671	AQP 8	Large flying fox/Megabat	*Pteropus vampyrus*	Laurasiatheria	Chiroptera	Pteropodidae
NP_001193536/ENSBTAP00000026884	AQP 8	Cow	*Bos taurus*	Laurasiatheria	Ruminantia	Bovidae
ENSTTRP00000014466	AQP 8	Bottlenosed dolphin	*Tursiops truncatus*	Laurasiatheria	Cetacea	Delphinidae
ENSSSCP00000008353	AQP 8	Pig	*Sus scrofa*	Laurasiatheria	Suina	Suidae
AAWR02036274/ENSECAP00000007437	AQP 8	Horse	*Equus caballus*	Laurasiatheria	Perissodactyla	Equidae
ACTA01052011/ENSAMEP00000007344	AQP 8	Giant panda	*Ailuropoda melanoleuca*	Laurasiatheria	Carnivora	Ursidae
ENSCAFP00000025764	AQP 8	Dog	*Canis lupus familiaris*	Laurasiatheria	Carnivora	Canidae
ENSFCAP00000014163	AQP 8	Domestic cat	*Felis catus*	Laurasiatheria	Carnivora	Felidae
ENSLAFP00000001748	AQP 8	African savanna elephant	*Loxodonta africana*	Afrotheria	Proboscidea	Elephantidae
ENSPCAP00000013157	AQP 8	Cape rock hyrax	*Procavia capensis*	Afrotheria	Hyracoidea	Procaviidae
ENSETEP00000010987	AQP 8	Lesser hedgehog tenrec	*Echinops telfairi*	Afrotheria	Afrosoricida	Tenrecidae
AAGV03222964/AAGV03222963/<br>ENSDNOP00000012347	AQP 8	Nine-banded armadillo	*Dasypus novemcinctus*	Xenarthra	Cingulata	Dasypodidae
ENSMODP00000020379	AQP 8	Gray short-tailed opossum	*Monodelphis domestica*	Metatheria	Didelphimorphia	Didelphidae
ENSOANP00000002345	AQP 8	Platypus	*Ornithorhynchus anatinus*	Prototheria	Monotremata	Ornithorhynchidae
ENSAPLP00000003161	AQP 8	Mallard	*Anas platyrhynchos*	Aves	Anseriformes	Anatidae
ENSMGAP00000006410	AQP 8	Turkey	*Meleagris gallopavo*	Aves	Galliformes	Phasianidae
ENSGALP00000009615	AQP 8	Chicken	*Gallus gallus*	Aves	Galliformes	Phasianidae
ENSXETP00000064129/NP_001107728	AQP 8	Western clawed frog	*Xenopus (Silurana) tropicalis*	Amphibia	Anura	Pipidae
ENSONIP00000024898	Aqp 8aa	Nile tilapia	*Oreochromis niloticus*	Acanthopterygii	Perciformes	Cichlidae
AANH01011088/ENSGACP00000012041	Aqp 8aa	Three-spined stickleback	*Gasterosteus aculeatus*	Acanthopterygii	Gasterosteiformes	Gasterosteidae
CU071487/CCAF010046511/CCAF010046510	Aqp 8aa1	Rainbow trout	*Oncorhynchus mykiss*	Protacanthopterygii	Salmoniformes	Salmonidae
AGKD01093852/AGKD01051269/DW573347	Aqp 8aa2	Atlantic salmon	*Salmo salar*	Protacanthopterygii	Salmoniformes	Salmonidae
EV367413	Aqp 8aa	Lake whitefish	*Coregonus clupeaformis*	Protacanthopterygii	Salmoniformes	Salmonidae
FJ655386/ENSDARP00000066381	Aqp 8aa	Zebrafish	*Danio rerio*	Ostariophysi	Cypriniformes	Cyprinidae
CK402738	Aqp 8aa	Blue catfish	*Ictalurus furcatus*	Ostariophysi	Siluriformes	Ictaluridae
ACQ57933/AWGY01152486	Aqp 8ab	Sablefish	*Anoplopoma fimbria*	Acanthopterygii	Scorpaeniformes	Anoplopomatidae
AANH01012285/ENSGACP00000019141	Aqp 8ab	Three-spined stickleback	*Gasterosteus aculeatus*	Acanthopterygii	Gasterosteiformes	Gasterosteidae
ENSORLP00000003812	Aqp 8ab	Japanese medaka	*Oryzias latipes*	Acanthopterygii	Beloniformes	Adrianichthyidae

CAEA01026729/CAEA01201958/EX741282	Aqp 8ab	Atlantic cod	*Gadus morhua*	Paracanthopterygii	Gadiformes	Gadidae
CU071568/CCAF010046510	Aqp 8ab1	Rainbow trout	*Oncorhynchus mykiss*	Protacanthopterygii	Salmoniformes	Salmonidae
AGKD01005999/DW532465	Aqp 8ab1	Atlantic salmon	*Salmo salar*	Protacanthopterygii	Salmoniformes	Salmonidae
DT342244	Aqp 8ab	Fathead minnow	*Pimephales promelas*	Ostariophysi	Cypriniformes	Cyprinidae
EU341834/ENSDARP00000096730	Aqp 8ab	Zebrafish	*Danio rerio*	Ostariophysi	Cypriniformes	Cyprinidae
CAAB02006724/ENSTRUP00000007329	Aqp 8bb	Torafugu	*Takifugu rubripes*	Acanthopterygii	Tetraodontiformes	Tetraodontidae
CAG11438/ENSTNIP00000021161	Aqp 8bb	Green-spotted pufferfish	*Tetraodon nigroviridis*	Acanthopterygii	Tetraodontiformes	Tetraodontidae
EB038315	Aqp 8bb	Atlantic halibut	*Hippoglossus hippoglossus*	Acanthopterygii	Pleuronectiformes	Pleuronectidae
DV567193	Aqp 8bb	European flounder	*Platichthys flesus*	Acanthopterygii	Pleuronectiformes	Pleuronectidae
JU402645/FE950965	Aqp 8bb	Turbot	*Scophthalmus maximus*	Acanthopterygii	Pleuronectiformes	Scophthalmidae
HS988011/DQ889225/ABK20159	Aqp 8bb	Gilthead seabream	*Sparus aurata*	Acanthopterygii	Perciformes	Sparidae
GAJJ01001641/GO620173/AWGY01064472	Aqp 8bb	Sablefish	*Anoplopoma fimbria*	Acanthopterygii	Scorpaeniformes	Anoplopomatidae
GE808463	Aqp 8bb	Copper rockfish	*Sebastes caurinus*	Acanthopterygii	Scorpaeniformes	Sebastidae
AANH01004389/ENSGACP00000015550	Aqp 8bb	Three-spined stickleback	*Gasterosteus aculeatus*	Acanthopterygii	Gasterosteiformes	Gasterosteidae
AGKD01119048/AGKD01156665/KC626880/ ACN11279	Aqp 8bb1	Atlantic salmon	*Salmo salar*	Protacanthopterygii	Salmoniformes	Salmonidae
FJ695516/ENSDARP00000005510	Aqp 8bb1	Zebrafish	*Danio rerio*	Ostariophysi	Cypriniformes	Cyprinidae
AEFG01050375/ENSPMAP00000006709	Aqp 8	Sea lamprey	*Petromyzon marinus*	Hyperoartia	Petromyzontiformes	Petromyzontidae
XP_002131566/ENSCINP00000011466	Aqp 8	Vase tunicate	*Ciona intestinalis*	Tunicata	Enterogona	Cionidae
ENSCSAVP00000018099	Aqp 8	Pacific transparent sea squirt	*Ciona savignyi*	Tunicata	Enterogona	Cionidae
XP_002599198	Aqp 8L1	Florida lancelet	*Branchiostoma floridae*	Cephalochordata	Amphioxiformes	Branchiostomidae
AAGJ04074400/XP_792735/XP_001197182/ XP_003730891	Aqp 8L1b	Purple sea urchin	*Strongylocentrotus purpuratus*	Echinodermata	Echinozoa	Strongylocentrotidae
**Protostomia Aqp8-like**						
AEP14560	Aqp 8L(6)	Water bear	*Milnesium tardigradum*	Tardigrada	Apochela	Milnesiidae
EKC40660	Aqp 8L1	Pacific oyster	*Crassostrea gigas*	Mollusca	Ostreoida	Ostreidae
EKC40659	Aqp 8L2	Pacific oyster	*Crassostrea gigas*	Mollusca	Ostreoida	Ostreidae
EKC40657	Aqp 8L3	Pacific oyster	*Crassostrea gigas*	Mollusca	Ostreoida	Ostreidae
EKC40658	Aqp 8L4	Pacific oyster	*Crassostrea gigas*	Mollusca	Ostreoida	Ostreidae
LotgiP156476	Aqp 8L1	Owl limpet	*Lottia gigantea*	Mollusca		Lottiidae
LotgiP196256	Aqp 8L2	Owl limpet	*Lottia gigantea*	Mollusca		Lottiidae
FX193841	Aqp 8L	Great pond snail	*Lymnaea stagnalis*	Mollusca		Lymnaeidae
XP_005094214	Aqp 8L1	California sea hare	*Aplysia californica*	Mollusca		Aplysiidae
XP_005098818	Aqp 8L2	California sea hare	*Aplysia californica*	Mollusca		Aplysiidae
XP_005109261	Aqp 8L3	California sea hare	*Aplysia californica*	Mollusca		Aplysiidae
C32C4.2	Aqp 8L(6)	Free-living roundworm	*Caenorhabditis elegans*	Nematoda	Rhabditida	Rhabditidae
F40F9.9	Aqp 8L(4)	Free-living roundworm	*Caenorhabditis elegans*	Nematoda	Rhabditida	Rhabditidae
PPA19447	Aqp 8L(6)	Diplogastrid nematode	*Pristionchus pacificus*	Nematoda	Diplogasterida	Neodiplogasteridae
CapteP142373	Aqp 8L	Segmented worm	*Capitella teleta*	Annelida	Capitellida	Capitellidae
CapteP166556	Aqp 8L	Segmented worm	*Capitella teleta*	Annelida	Capitellida	Capitellidae
CapteP219373	Aqp 8L	Segmented worm	*Capitella teleta*	Annelida	Capitellida	Capitellidae
**Porifera**						
EC374967	Aqp 8L	Slime sponge	*Oscarella carmela*	Porifera	Homosclerophorida	Plakinidae
CBY89223	Aqp 8L	Siliceous sponge	*Suberites domuncula*	Porifera	Hadromerida	Suberitidae
**Protista, Fungi Aqp8-like**						
DDB0191271/XP_641629/BAA85158	AQP A	Cellular slime mold	*Dictyostelium discoideum*	Amoebozoa	Dictyosteliida	
AJWI01000352	AQP A	Cellular slime mold	*Dictyostelium intermedium*	Amoebozoa	Dictyosteliida	
XP_003283881	AQP A	Cellular slime mold	*Dictyostelium purpureum*	Amoebozoa	Dictyosteliida	
EGG21443	AQP A	Cellular slime mold	*Dictyostelium fasciculatum*	Amoebozoa	Dictyosteliida	
ADBJ01000050	AQP A	Cellular slime mold	*Polysphondylium pallidum*	Amoebozoa	Dictyosteliida	
DDB0214915	Wac A	Cellular slime mold	*Dictyostelium discoideum*	Amoebozoa	Dictyosteliida	
AJWI01007140	Wac A	Cellular slime mold	*Dictyostelium intermedium*	Amoebozoa	Dictyosteliida	
ADID01000158	Wac A	Cellular slime mold	*Dictyostelium purpureum*	Amoebozoa	Dictyosteliida	
AJWJ01000122	Wac A	Cellular slime mold	*Polysphondylium violaceum*	Amoebozoa	Dictyosteliida	
ADBJ01000047	Wac A	Cellular slime mold	*Polysphondylium pallidum*	Amoebozoa	Dictyosteliida	
DDB0205768	AQP P	Cellular slime mold	*Dictyostelium discoideum*	Amoebozoa	Dictyosteliida	
AJWI01001707	AQP P	Cellular slime mold	*Dictyostelium intermedium*	Amoebozoa	Dictyosteliida	
ADID01000883	AQP P	Cellular slime mold	*Dictyostelium purpureum*	Amoebozoa	Dictyosteliida	
ADHC01000009	AQP P	Cellular slime mold	*Dictyostelium fasciculatum*	Amoebozoa	Dictyosteliida	
EFA81707	AQP P	Cellular slime mold	*Polysphondylium pallidum*	Amoebozoa	Dictyosteliida	
BAF75061	AQP	Amoeba	*Amoeba proteus*	Amoebozoa	Tubulinida	Amoebidae
XP_004339464	AQP	Amoeba	*Acanthamoeba castellanii*	Amoebozoa	Centramoebida	Acanthamoebidae
XP_001527487	Aqy 1	Fungus	*Lodderomyces elongisporus*	Ascomycota	Saccharomycetales	Debaryomycetaceae
CCG20420	Aqy 1	Fungus	*Candida orthopsilosis*	Ascomycota	Saccharomycetales	
EGW34382	Aqy 1	Fungus	*Spathaspora passalidarum*	Ascomycota	Saccharomycetales	Debaryomycetaceae
XP_001383665	Aqy 1	Pichia stipitis	*Scheffersomyces stipitis*	Ascomycota	Saccharomycetales	Debaryomycetaceae
YPR192W	Aqy 1	Baker´s yeast	*Saccharomyces cerevisiae*	Ascomycota	Saccharomycetales	Saccharomycetaceae
**Aquaglyceroporins**						
**Deuterostomia aquaglyceroporins**						
ENSP00000297991	AQP 3	Human	*Homo sapiens*	Euarchontoglires	Primates	Hominidae
ENSPTRP00000035672	AQP 3	Chimpanzee	*Pan troglodytes*	Euarchontoglires	Primates	Hominidae
ENSGGOP00000003917	AQP 3	Western lowland gorilla	*Gorilla gorilla gorilla*	Euarchontoglires	Primates	Hominidae
ENSPPYP00000021438	AQP 3	Sumatran orangutan	*Pongo abelii*	Euarchontoglires	Primates	Hominidae
ENSNLEP00000006154	AQP 3	Northern white-cheeked gibbon	*Nomascus leucogenys*	Euarchontoglires	Primates	Hylobatidae
ENSMMUP00000004418	AQP 3	Rhesus macaque	*Macaca mulatta*	Euarchontoglires	Primates	Cercopithecidae
ENSP00000297991	AQP 3	Hamadryas baboon	*Papio hamadryas*	Euarchontoglires	Primates	Cercopithecidae
AHZZ01031032	AQP 3	Olive baboon	*Papio anubis*	Euarchontoglires	Primates	Cercopithecidae
ENSCJAP00000015149	AQP 3	White-tufted-ear marmoset	*Callithrix jacchus*	Euarchontoglires	Primates	Cebidae
XP_003939792	AQP 3	Bolivian squirrel monkey	*Saimiri boliviensis boliviensis*	Euarchontoglires	Primates	Cebidae
ENSTSYP00000010301	AQP 3	Philippine tarsier	*Tarsius syrichta*	Euarchontoglires	Primates	Tarsiidae
ENSMICP00000011053	AQP 3	Gray mouse lemur	*Microcebus murinus*	Euarchontoglires	Primates	Cheirogaleidae
XP_003800331/ENSOGAP00000012860	AQP 3	Small-eared galago/Bushbaby	*Otolemur garnettii*	Euarchontoglires	Primates	Galagidae
ENSTBEP00000002696	AQP 3	Northern tree shrew	*Tupaia belangeri*	Euarchontoglires	Scandentia	Tupaiidae
ELW70260	AQP 3	Chinese tree shrew	*Tupaia chinensis*	Euarchontoglires	Scandentia	Tupaiidae
ENSMUST00000055327	AQP 3	Mouse	*Mus musculus*	Euarchontoglires	Rodentia	Muridae
ENSRNOP00000013803	AQP 3	Norway rat	*Rattus norvegicus*	Euarchontoglires	Rodentia	Muridae
XP_003512012	AQP 3	Chinese hamster	*Cricetulus griseus*	Euarchontoglires	Rodentia	Cricetidae
ENSDORP00000005634	AQP 3	Ord's kangaroo rat	*Dipodomys ordii*	Euarchontoglires	Rodentia	Heteromyidae
ENSCPOP00000011041	AQP 3	Domestic guinea pig	*Cavia porcellus*	Euarchontoglires	Rodentia	Caviidae
EHB02657	AQP 3	Naked mole-rat	*Heterocephalus glaber*	Euarchontoglires	Rodentia	Bathyergidae
ENSSTOP00000014181	AQP 3	Thirteen-lined ground squirrel	*Ictidomys tridecemlineatus*	Euarchontoglires	Rodentia	Sciuridae
ENSOPRP00000015348	AQP 3	American pika	*Ochotona princeps*	Euarchontoglires	Lagomorpha	Ochotonidae
ENSOCUP00000015333	AQP 3	Rabbit	*Oryctolagus cuniculus*	Euarchontoglires	Lagomorpha	Leporidae
ENSEEUP00000000364	AQP 3	Western European hedgehog	*Erinaceus europaeus*	Laurasiatheria	Insectivora	Erinaceinae
ENSSARP00000001379	AQP 3	European shrew	*Sorex araneus*	Laurasiatheria	Insectivora	Soricidae
BAF49644	AQP 3	House shrew	*Suncus murinus*	Laurasiatheria	Insectivora	Soricidae
ENSPVAP00000010827	AQP 3	Large flying fox/Megabat	*Pteropus vampyrus*	Laurasiatheria	Chiroptera	Pteropodidae

ELK09077	AQP 3	Black flying fox	*Pteropus alecto*	Laurasiatheria	Chiroptera	Pteropodidae
ENSMLUP00000011127	AQP 3	Little brown bat/Microbat	*Myotis lucifugus*	Laurasiatheria	Chiroptera	Vespertilionidae
ELK34287	AQP 3	David´s myotis	*Myotis davidii*	Laurasiatheria	Chiroptera	Vespertilionidae
XP_004005296/ENSP00000297991/ACIV010833122	AQP 3	Sheep	*Ovis aries*	Laurasiatheria	Ruminantia	Bovidae
ENSBTAP00000011196	AQP 3	Cow	*Bos taurus*	Laurasiatheria	Ruminantia	Bovidae
ELR51091	AQP 3	Yak	*Bos grunniens mutus*	Laurasiatheria	Ruminantia	Bovidae
ENSTTRP00000010032	AQP 3	Bottlenosed dolphin	*Tursiops truncatus*	Laurasiatheria	Cetacea	Delphinidae
ANOL02039846/XP_004275133	AQP 3	Killer whale	*Orcinus orca*	Laurasiatheria	Cetacea	Delphinidae
ENSSSCP00000011724	AQP 3	Pig	*Sus scrofa*	Laurasiatheria	Suina	Suidae
ENSVPAP00000005209	AQP 3	Alpaca	*Vicugna pacos*	Laurasiatheria	Tylopoda	Camelidae
ENSECAP00000018712	AQP 3	Horse	*Equus caballus*	Laurasiatheria	Perissodactyla	Equidae
ENSAMEP00000007833	AQP 3	Giant panda	*Ailuropoda melanoleuca*	Laurasiatheria	Carnivora	Ursidae
ENSCAFP00000002716	AQP 3	Dog	*Canis lupus familiaris*	Laurasiatheria	Carnivora	Canidae
ENSCAFP00000002716	AQP 3	Domestic ferret	*Mustela putorius furo*	Laurasiatheria	Carnivora	Mustelidae
ENSFCAP00000002505	AQP 3	Domestic cat	*Felis catus*	Laurasiatheria	Carnivora	Felidae
ENSPCAP00000015510	AQP 3	Cape rock hyrax	*Procavia capensis*	Afrotheria	Hyracoidea	Procaviidae
ENSLAFP00000018135	AQP 3	African savanna elephant	*Loxodonta africana*	Afrotheria	Proboscidea	Elephantidae
ENSETEP00000014787	AQP 3	Lesser hedgehog tenrec	*Echinops telfairi*	Afrotheria	Afrosoricida	Tenrecidae
AHIN01023994	AQP 3	Florida manatee	*Trichechus manatus latirostris*	Afrotheria	Sirenia	Trichechidae
ALYB01234330	AQP 3	Ardvaark	*Orycteropus afer afer*	Afrotheria	Tubulidentata	Orycteropodidae
ENSDNOP00000017578	AQP 3	Nine-banded armadillo	*Dasypus novemcinctus*	Xenarthra	Cingulata	Dasypodidae
ABVD01594697	AQP 3	Hoffmann's two-fingered sloth	*Choloepus hoffmanni*	Xenarthra	Pilosa	Megalonychidae
ENSMEUP00000013288	AQP 3	Tammar wallaby	*Macropus eugenii*	Metatheria	Diprotodontia	Macropodidae
ENSSHAP00000003616	AQP 3	Tasmanian devil	*Sarcophilus harrisii*	Metatheria	Dasyuromorphia	Dasyuridae
ENSMODP00000004690	AQP 3	Gray short-tailed opossum	*Monodelphis domestica*	Metatheria	Didelphimorphia	Didelphidae
ENSOANP00000023098	AQP 3	Platypus	*Ornithorhynchus anatinus*	Prototheria	Monotremata	Ornithorhynchidae
AGAI01048038/AGAI01048039	AQP 3	Budgerigar	*Melopsittacus undulatus*	Aves	Psittaciformes	Psittacidae
AMXX01081206/AMXX01126069/AOUJ01111547/ AOUJ01246249	AQP 3	Scarlet macaw	*Ara macao*	Aves	Psittaciformes	Psittacidae
CAVT010029651/CAVT010029652	AQP 3	Common canary	*Serinus canaria*	Aves	Passeriformes	Fringillidae
AKZB01095700/AKZB01095701	AQP 3	Medium ground finch	*Geospiza fortis*	Aves	Passeriformes	Fringillidae
ENSTGUP00000001721	AQP 3	Zebra finch	*Taeniopygia guttata*	Aves	Passeriformes	Estrildidae
AGTO01001363	AQP 3	Collared flycatcher	*Ficedula albicollis*	Aves	Passeriformes	Muscicapidae
ENSAPLP00000004013	AQP 3	Mallard	*Anas platyrhynchos*	Aves	Anseriformes	Anatidae
ACF19804	AQP 3	Common quail	*Coturnix coturnix*	Aves	Galliformes	Phasianidae
ENSMGAP00000001305	AQP 3	Turkey	*Meleagris gallopavo*	Aves	Galliformes	Phasianidae
ENSGALP00000003859	AQP 3	Chicken	*Gallus gallus*	Aves	Galliformes	Phasianidae
AKHW01039336/AKHW01039339	AQP 3	American alligator	*Alligator mississippiensis*	Archosauria	Crocodylia	Alligatoridae
AVPB01026904	AQP 3	Chinese alligator	*Alligator sinensis*	Archosauria	Crocodylia	Alligatoridae
ENSPSIP00000017085	AQP 3	Chinese softshell turtle	*Pelodiscus sinensis*	Sauropsida	Testudines	Trionychidae
EMP32512	AQP 3	Green seaturtle	*Chelonia mydas*	Sauropsida	Testudines	Cheloniidae
GANJ01003270	AQP 3	Reeves's turtle	*Mauremys reevesii*	Sauropsida	Testudines	Geoemydidae
AHGY01159137/AHGY01159138	AQP 3	Western painted turtle	*Chrysemys picta bellii*	Sauropsida	Testudines	Emydidae
AEH96275	AQP 3	Salt marsh snake	*Nerodia clarkii clarkii*	Lepidosauria	Squamata	Colubridae
AEH96276	AQP 3	Banded water snake	*Nerodia fasciata*	Lepidosauria	Squamata	Colubridae
ETE57537/AZIM01031376/AZIM01008241	AQP 3	King cobra	*Ophiophagus hannah*	Lepidosauria	Squamata	Elapidae
JAB54667	AQP 3	Eastern coral snake	*Micrurus fulvius*	Lepidosauria	Squamata	Elapidae
AEH96274	AQP 3	Broad-banded blue sea krait	*Laticauda semifasciata*	Lepidosauria	Squamata	Elapidae
AFJ49443	AQP 3	Eastern diamondback rattlesnake	*Crotalus adamanteus*	Lepidosauria	Squamata	Viperidae
GAAZ01000200	AQP 3	Timber rattlesnake	*Crotalus horridus*	Lepidosauria	Squamata	Viperidae
AEQU02198269/AEQU02198273/AEQU02198274	AQP 3	Burmese python	*Python molurus bivittatus*	Lepidosauria	Squamata	Pythonidae
ENSACAP00000012483	AQP 3	Green anole	*Anolis carolinensis*	Lepidosauria	Squamata	Iguanidae
ABC98210	AQP 3	Southern gray treefrog	*Hyla chrysoscelis*	Amphibia	Anura	Hylidae
ACM18196	AQP 3	Dark-spotted frog	*Pelophylax nigromaculatus*	Amphibia	Anura	Ranidae
ACM51136	AQP 3	Asiatic toad	*Bufo gargarizans*	Amphibia	Anura	Bufonidae
NP_001081876	AQP 3	African clawed frog	*Xenopus laevis*	Amphibia	Anura	Pipidae
AAI58299/ENSXETP00000004583	AQP 3	Western clawed frog	*Xenopus (Silurana) tropicalis*	Amphibia	Anura	Pipidae
GAQK01000665	AQP 3	Chinese salamander	*Hynobius chinensis*	Amphibia	Caudata	Hynobiidae
AFYH01154020/AFYH01154029/AFYH01154028/ AFYH01154027/AFYH01154026	Aqp 3	Coelacanth	*Latimeria chalumnae*	Actinistia	Coelacanthiformes	Coelacanthidae
ENSTRUP00000012922	Aqp 3a	Torafugu	*Takifugu rubripes*	Acanthopterygii	Tetraodontiformes	Tetraodontidae
AOOT01001915/AOOT01001916/AOOT01001917	Aqp 3a	Sansaifugu	*Takifugu flavidus*	Acanthopterygii	Tetraodontiformes	Tetraodontidae
ADG86338	Aqp 3a	Mefugu	*Takifugu obscurus*	Acanthopterygii	Tetraodontiformes	Tetraodontidae
ENSTNIP00000006428	Aqp 3a	Green-spotted pufferfish	*Tetraodon nigroviridis*	Acanthopterygii	Tetraodontiformes	Tetraodontidae
GAAQ01004266	Aqp 3a	Dover sole	*Solea solea*	Acanthopterygii	Pleuronectiformes	Soleidae
AGRG01019534/AGRG01019534	Aqp 3a	Tongue sole	*Cynoglossus semilaevis*	Acanthopterygii	Pleuronectiformes	Cynoglossidae
DQ333306	Aqp 3a	Gold-lined seabream	*Rhabdosargus sarba*	Acanthopterygii	Perciformes	Sparidae
AGTA02008491/AGTA02008490	Aqp 3aa	Zebra mbuna	*Maylandia zebra*	Acanthopterygii	Perciformes	Cichlidae
AFNX01003871/AFNX01003872	Aqp 3aa	Red mwanza	*Pundamilia nyererei*	Acanthopterygii	Perciformes	Cichlidae
AFNY01011364	Aqp 3aa	Lyretail cichlid	*Neolamprologus brichardi*	Acanthopterygii	Perciformes	Cichlidae
AFNZ01010497/AFNZ01010496	Aqp 3aa	Burton's mouthbrooder	*Haplochromis burtoni*	Acanthopterygii	Perciformes	Cichlidae
ENSONIP00000020098	Aqp 3aa	Nile tilapia	*Oreochromis niloticus*	Acanthopterygii	Perciformes	Cichlidae
AGTA02008478/AGTA02008480	Aqp 3ab	Zebra mbuna	*Maylandia zebra*	Acanthopterygii	Perciformes	Cichlidae
AFNX01003878	Aqp 3ab	Red mwanza	*Pundamilia nyererei*	Acanthopterygii	Perciformes	Cichlidae
AFNY01011353/AFNY01011355	Aqp 3ab	Lyretail cichlid	*Neolamprologus brichardi*	Acanthopterygii	Perciformes	Cichlidae
DY630346/AFNZ01010486/AFNZ01010487	Aqp 3ab	Burton's mouthbrooder	*Haplochromis burtoni*	Acanthopterygii	Perciformes	Cichlidae
ENSONIP00000020110	Aqp 3ab	Nile tilapia	*Oreochromis niloticus*	Acanthopterygii	Perciformes	Cichlidae
BAD20708	Aqp 3ab	Mozamique tilapia	*Oreochromis mossambicus*	Acanthopterygii	Perciformes	Cichlidae
BJ686705	Aqp 3ab	Victoria thick-lips mouthbrooder	*Haplochromis chilotes*	Acanthopterygii	Perciformes	Cichlidae
BJ690402	Aqp 3ab	Redtail sheller	*Haplochromis sp.*	Acanthopterygii	Perciformes	Cichlidae
BADN01029189	Aqp 3a	Pacific bluefin tuna	*Thunnus orientalis*	Acanthopterygii	Perciformes	Scombridae
AWGY01142399	Aqp 3aa	Sablefish	*Anoplopoma fimbria*	Acanthopterygii	Scorpaeniformes	Anoplopomatidae
AWGY01126917	Aqp 3ab	Sablefish	*Anoplopoma fimbria*	Acanthopterygii	Scorpaeniformes	Anoplopomatidae
AUPQ01054323	Aqp 3a	Flag rockfish	*Sebastes rubrivinctus*	Acanthopterygii	Scorpaeniformes	Sebastidae
AUPR01136281/AUPR01181854	Aqp 3a	Tiger rockfish	*Sebastes nigrocinctus*	Acanthopterygii	Scorpaeniformes	Sebastidae
ENSGACP00000013694	Aqp 3a	Three-spined stickleback	*Gasterosteus aculeatus*	Acanthopterygii	Gasterosteiformes	Gasterosteidae
ENSORLP00000012759	Aqp 3a	Japanese medaka	*Oryzias latipes*	Acanthopterygii	Beloniformes	Adrianichthyidae
GAIB01128398/JZ265611	Aqp 3a	Turquoise killifish	*Nothobranchius furzeri*	Acanthopterygii	Cyprinodontiformes	Nothobranchiidae
ACI49539	Aqp 3a	Common mummichog	*Fundulus heteroclitus*	Acanthopterygii	Cyprinodontiformes	Fundulidae
ES375494	Aqp 3a	Guppy	*Poecilia reticulata*	Acanthopterygii	Cyprinodontiformes	Poeciliidae
AYCK01000894	Aqp 3a	Amazon molly	*Poecilia formosa*	Acanthopterygii	Cyprinodontiformes	Poeciliidae
ENSXMAP00000019454	Aqp 3a	Southern platyfish	*Xiphophorus maculatus*	Acanthopterygii	Cyprinodontiformes	Poeciliidae
FF416557/ENSGMOP00000000666	Aqp 3a	Atlantic cod	*Gadus morhua*	Paracanthopterygii	Gadiformes	Gadidae
CCAF010038670	Aqp 3a1	Rainbow trout	*Oncorhynchus mykiss*	Protacanthopterygii	Salmoniformes	Salmonidae
AGKD01094322/AGKD01056897	Aqp 3a1	Atlantic salmon	*Salmo salar*	Protacanthopterygii	Salmoniformes	Salmonidae
CCAF010040466	Aqp 3a2	Rainbow trout	*Oncorhynchus mykiss*	Protacanthopterygii	Salmoniformes	Salmonidae
AGKD01068849/AGKD01149541	Aqp 3a2	Atlantic salmon	*Salmo salar*	Protacanthopterygii	Salmoniformes	Salmonidae
EX885773	Aqp 3a	Common carp	*Cyprinus carpio*	Ostariophysi	Cypriniformes	Cyprinidae
AM928312	Aqp 3a	Goldfish	*Carassius auratus*	Ostariophysi	Cypriniformes	Cyprinidae
BAB83082	Aqp 3a	Big-scaled redfin	*Tribolodon hakonensis*	Ostariophysi	Cypriniformes	Cyprinidae

DT246747	Aqp 3a	Fathead minnow	*Pimephales promelas*	Ostariophysi	Cypriniformes	Cyprinidae
GAHO01101838/GAHO01080011/GAHO01065997/GAHO01045963/ GAHO01051616/GAHO01060873	Aqp 3a	Small gill opening goldenline barbel	*Sinocyclocheilus angustiporus*	Ostariophysi	Cypriniformes	Cyprinidae
GAHL01086926/GAHL01011211	Aqp 3a	Blind goldenline barbel	*Sinocyclocheilus anophthalmus*	Ostariophysi	Cypriniformes	Cyprinidae
EU341833/ENSDARP00000018463	Aqp 3a	Zebrafish	*Danio rerio*	Ostariophysi	Cypriniformes	Cyprinidae
GAAD01002505	Aqp 3a	Oriental weatherfish	*Misgurnus anguillicaudatus*	Ostariophysi	Cypriniformes	Cobitidae
APWO01112913	Aqp 3a	Mexican tetra	*Astyanax mexicanus*	Ostariophysi	Characiformes	Characidae
CV987951	Aqp 3a	Channel catfish	*Ictalurus punctatus*	Ostariophysi	Siluriformes	Ictaluridae
GAGX01002847	Aqp 3a	Brown bullhead	*Ameiurus nebulosus*	Ostariophysi	Siluriformes	Ictaluridae
AVPY01014543/AVPY01014545/AVPY01014546	Aqp 3a	Japanese eel	*Anguilla japonica*	Elopomorpha	Anguilliformes	Anguillidae
BADN01004527/BADN01004528	Aqp 3b	Pacific bluefin tuna	*Thunnus orientalis*	Acanthopterygii	Perciformes	Scombridae
AWGY01142399	Aqp 3b	Sablefish	*Anoplopoma fimbria*	Acanthopterygii	Scorpaeniformes	Anoplopomatidae
AUPQ01059403/AUPQ01059404/AUPQ01102314/AUPQ01104729	Aqp 3b	Flag rockfish	*Sebastes rubrivinctus*	Acanthopterygii	Scorpaeniformes	Sebastidae
AUPR01165934/AUPR01010211/AUPR01176565/AUPR01194884	Aqp 3b	Tiger rockfish	*Sebastes nigrocinctus*	Acanthopterygii	Scorpaeniformes	Sebastidae
FF411765/CAEA01538197/ENSGMOP00000008635	Aqp 3b	Atlantic cod	*Gadus morhua*	Paracanthopterygii	Gadiformes	Gadidae
CAEA01538157/ENSGMOP00000017976	Aqp 3b	Atlantic cod	*Gadus morhua*	Paracanthopterygii	Gadiformes	Gadidae
CU071888/CCAF010016865	Aqp 3b1	Rainbow trout	*Oncorhynchus mykiss*	Protacanthopterygii	Salmoniformes	Salmonidae
AGKD01002624	Aqp 3b1	Atlantic salmon	*Salmo salar*	Protacanthopterygii	Salmoniformes	Salmonidae
CCAF010164985	Aqp 3b2	Rainbow trout	*Oncorhynchus mykiss*	Protacanthopterygii	Salmoniformes	Salmonidae
AGKD01215587	Aqp 3b2	Atlantic salmon	*Salmo salar*	Protacanthopterygii	Salmoniformes	Salmonidae
DT136147	Aqp 3b	Fathead minnow	*Pimephales promelas*	Ostariophysi	Cypriniformes	Cyprinidae
GAHO01051838/GAHO01061212	Aqp 3b	Small gill opening goldenline barbel	*Sinocyclocheilus angustiporus*	Ostariophysi	Cypriniformes	Cyprinidae
GAHL01027245/GAHL01037208	Aqp 3b	Blind goldenline barbel	*Sinocyclocheilus anophthalmus*	Ostariophysi	Cypriniformes	Cyprinidae
EU341832/ENSDARP00000092015	Aqp 3b	Zebrafish	*Danio rerio*	Ostariophysi	Cypriniformes	Cyprinidae
APWO01038715	Aqp 3b	Mexican tetra	*Astyanax mexicanus*	Ostariophysi	Characiformes	Characidae
CAC85286	Aqp 3b	European eel	*Anguilla anguilla*	Elopomorpha	Anguilliformes	Anguillidae
BAH89253/GAGT01000112/AVPY01155731	Aqp 3b	Japanese eel	*Anguilla japonica*	Elopomorpha	Anguilliformes	Anguillidae
AHAT01012409/ENSLOCP00000006040	Aqp 3	Spotted gar	*Lepisosteus oculatus*	Holostei	Semionotiformes	Lepisosteidae
AESE012529808/AESE010180002/AESE010640639/AESE010645223/AESE010899949	Aqp 3	Little skate	*Leucoraja erinacea*	Chondrichthyes	Rajiformes	Rajidae
AESE010521158/AESE010679026/AESE010221296/AESE011697121	Aqp 3L	Little skate	*Leucoraja erinacea*	Chondrichthyes	Rajiformes	Rajidae
AAVX02016279	Aqp 3	Ghost shark	*Callorhinchus milii*	Chondrichthyes	Chimaeriformes	Callorhinchidae
AAVX02016279	Aqp 3L	Ghost shark	*Callorhinchus milii*	Chondrichthyes	Chimaeriformes	Callorhinchidae
KJ784518/AEFG01033607/ENSPMAP00000008130	Aqp 3L1	Sea lamprey	*Petromyzon marinus*	Hyperoartia	Petromyzontiformes	Petromyzontidae
APJL01047447/APJL01047446/APJL01047445	Aqp 3L1	Arctic lamprey	*Lethenteron camtschaticum*	Hyperoartia	Petromyzontiformes	Petromyzontidae
KJ784520/AEFG01042179	Aqp 3L2	Sea lamprey	*Petromyzon marinus*	Hyperoartia	Petromyzontiformes	Petromyzontidae
APJL01034901/APJL01034902	Aqp 3L2	Arctic lamprey	*Lethenteron camtschaticum*	Hyperoartia	Petromyzontiformes	Petromyzontidae
ENSP00000316510/ENST00000322309	AQP 7p4	Human	*Homo sapiens*	Euarchontoglires	Primates	Hominidae
ENSP00000329634/ENST00000428759	AQP 7p3	Human	*Homo sapiens*	Euarchontoglires	Primates	Hominidae
ENST00000453967	AQP 7p2	Human	*Homo sapiens*	Euarchontoglires	Primates	Hominidae
ENSP00000335588	AQP 7p1	Human	*Homo sapiens*	Euarchontoglires	Primates	Hominidae
ENSP00000456868	AQP 7	Human	*Homo sapiens*	Euarchontoglires	Primates	Hominidae
ENSP00000368821	AQP 7	Human	*Homo sapiens*	Euarchontoglires	Primates	Hominidae
ENSPTRP00000035670	AQP 7	Chimpanzee	*Pan troglodytes*	Euarchontoglires	Primates	Hominidae
ENSGGOP00000004884	AQP 7	Western lowland gorilla	*Gorilla gorilla gorilla*	Euarchontoglires	Primates	Hominidae
ENSPPYP00000021439	AQP 7	Sumatran orangutan	*Pongo abelii*	Euarchontoglires	Primates	Hominidae
ENSNLEP00000006164	AQP 7	Northern white-cheeked gibbon	*Nomascus leucogenys*	Euarchontoglires	Primates	Hylobatidae
ENSMMUP00000039362	AQP 7	Rhesus macaque	*Macaca mulatta*	Euarchontoglires	Primates	Cercopithecidae
ENSMMUP00000039362	AQP 7	Crab-eating macaque	*Macaca fascicularis*	Euarchontoglires	Primates	Cercopithecidae
GENSCAN00000041650	AQP 7	Hamadryas baboon	*Papio hamadryas*	Euarchontoglires	Primates	Cercopithecidae
AHZZ01080412	AQP 7	Olive baboon	*Papio anubis*	Euarchontoglires	Primates	Cercopithecidae
ENSCJAP00000015117	AQP 7	White-tufted-ear marmoset	*Callithrix jacchus*	Euarchontoglires	Primates	Cebidae
XP_003939791	AQP 7	Bolivian squirrel monkey	*Saimiri boliviensis boliviensis*	Euarchontoglires	Primates	Cebidae
ENSTSYP00000000772	AQP 7	Philippine tarsier	*Tarsius syrichta*	Euarchontoglires	Primates	Tarsiidae
ENSMICP00000011819	AQP 7	Gray mouse lemur	*Microcebus murinus*	Euarchontoglires	Primates	Cheirogaleidae
ENSOGAP00000000692	AQP 7	Small-eared galago/Bushbaby	*Otolemur garnettii*	Euarchontoglires	Primates	Galagidae
ENSTBEP00000005344	AQP 7	Northern tree shrew	*Tupaia belangeri*	Euarchontoglires	Scandentia	Tupaiidae
ELW70259	AQP 7	Chinese tree shrew	*Tupaia chinensis*	Euarchontoglires	Scandentia	Tupaiidae
ENSMUSP00000030136	AQP 7	Mouse	*Mus musculus*	Euarchontoglires	Rodentia	Muridae
ENSRNOP00000012974	AQP 7	Norway rat	*Rattus norvegicus*	Euarchontoglires	Rodentia	Muridae
XP_003512015	AQP 7	Chinese hamster	*Cricetulus griseus*	Euarchontoglires	Rodentia	Cricetidae
ABRO01459270	AQP 7	Ord's kangaroo rat	*Dipodomys ordii*	Euarchontoglires	Rodentia	Heteromyidae
ENSCPOP00000011038	AQP 7	Domestic guinea pig	*Cavia porcellus*	Euarchontoglires	Rodentia	Caviidae
EHB02656	AQP 7	Naked mole-rat	*Heterocephalus glaber*	Euarchontoglires	Rodentia	Bathyergidae
ENSSTOP00000006294	AQP 7	Thirteen-lined ground squirrel	*Ictidomys tridecemlineatus*	Euarchontoglires	Rodentia	Sciuridae
ENSOPRP00000015343	AQP 7	American pika	*Ochotona princeps*	Euarchontoglires	Lagomorpha	Ochotonidae
ENSOCUP00000015329	AQP 7	Rabbit	*Oryctolagus cuniculus*	Euarchontoglires	Lagomorpha	Leporidae
ENSEEUP00000002340	AQP 7	Western European hedgehog	*Erinaceus europaeus*	Laurasiatheria	Insectivora	Erinaceinae
ENSSARP00000008852	AQP 7	Common shrew	*Sorex araneus*	Laurasiatheria	Insectivora	Soricidae
ENSPVAP00000010825	AQP 7	Large flying fox/Megabat	*Pteropus vampyrus*	Laurasiatheria	Chiroptera	Pteropodidae
ELK09078	AQP 7	Black flying fox	*Pteropus alecto*	Laurasiatheria	Chiroptera	Pteropodidae
ELK34288	AQP 7	David´s myotis	*Myotis davidii*	Laurasiatheria	Chiroptera	Vespertilionidae
ENSECAP00000011814	AQP 7	Sheep	*Ovis aries*	Laurasiatheria	Ruminantia	Bovidae
ENSBTAP00000026782	AQP 7	Cow	*Bos taurus*	Laurasiatheria	Ruminantia	Bovidae
ELR51090	AQP 7	Yak	*Bos grunniens mutus*	Laurasiatheria	Ruminantia	Bovidae
ENSTTRP00000010030	AQP 7	Bottlenosed dolphin	*Tursiops truncatus*	Laurasiatheria	Cetacea	Delphinidae
ANOL02039845/XP_004275132	AQP 7	Killer whale	*Orcinus orca*	Laurasiatheria	Cetacea	Delphinidae
ENSSSCP00000011725	AQP 7	Pig	*Sus scrofa*	Laurasiatheria	Suina	Suidae
ENSVPAP00000001206	AQP 7	Alpaca	*Vicugna pacos*	Laurasiatheria	Tylopoda	Camelidae
ENSECAP00000011814	AQP 7	Horse	*Equus caballus*	Laurasiatheria	Perissodactyla	Equidae
ENSAMEP00000007857	AQP 7	Giant panda	*Ailuropoda melanoleuca*	Laurasiatheria	Carnivora	Ursidae
ENSCAFP00000002708	AQP 7	Dog	*Canis lupus familiaris*	Laurasiatheria	Carnivora	Canidae
ENSCAFP00000002706	AQP 7	Domestic ferret	*Mustela putorius furo*	Laurasiatheria	Carnivora	Mustelidae
ENSFCAP00000002506	AQP 7	Domestic cat	*Felis catus*	Laurasiatheria	Carnivora	Felidae
ENSPCAP00000006546	AQP 7	Cape rock hyrax	*Procavia capensis*	Afrotheria	Hyracoidea	Procaviidae
ENSLAFP00000023877	AQP 7	African savanna elephant	*Loxodonta africana*	Afrotheria	Proboscidea	Elephantidae
AAIY02212605	AQP 7	Lesser hedgehog tenrec	*Echinops telfairi*	Afrotheria	Afrosoricida	Tenrecidae
AHIN01023999	AQP 7	Florida manatee	*Trichechus manatus latirostris*	Afrotheria	Sirenia	Trichechidae
ALYB01234333	AQP 7	Ardvaark	*Orycteropus afer afer*	Afrotheria	Tubulidentata	Orycteropodidae
ENSDNOP00000010742	AQP 7	Nine-banded armadillo	*Dasypus novemcinctus*	Xenarthra	Cingulata	Dasypodidae
ABVD01677639	AQP 7	Hoffmann's two-fingered sloth	*Choloepus hoffmanni*	Xenarthra	Pilosa	Megalonychidae
ENSMEUP00000001313	AQP 7	Tammar wallaby	*Macropus eugenii*	Metatheria	Diprotodontia	Macropodidae
ENSSHAP00000013186	AQP 7	Tasmanian devil	*Sarcophilus harrisii*	Metatheria	Dasyuromorphia	Dasyuridae
ENSMODP00000001460	AQP 7	Gray short-tailed opossum	*Monodelphis domestica*	Metatheria	Didelphimorphia	Didelphidae
AAPN01426604	AQP 7	Platypus	*Ornithorhynchus anatinus*	Prototheria	Monotremata	Ornithorhynchidae
TGUHOMP00000001711	AQP 7	Budgerigar	*Melopsittacus undulatus*	Aves	Psittaciformes	Psittacidae
AMXX01077757/AMXX01105423/AOUJ01157177/AOUJ01157173/AOCU01033654/AOUJ01039524	AQP 7	Scarlet macaw	*Ara macao*	Aves	Psittaciformes	Psittacidae
CAVT010029656	AQP 7	Common canary	*Serinus canaria*	Aves	Passeriformes	Fringillidae

AKZB01095704	AQP 7	Medium ground finch	*Geospiza fortis*	Aves	Passeriformes	Fringillidae
ENSTGUP00000001711	AQP 7	Zebra finch	*Taeniopygia guttata*	Aves	Passeriformes	Estrildidae
AGTO01001363	AQP 7	Collared flycatcher	*Ficedula albicollis*	Aves	Passeriformes	Muscicapidae
ENSAPLP00000004144	AQP 7	Mallard	*Anas platyrhynchos*	Aves	Anseriformes	Anatidae
ENSMGAP00000001223	AQP 7	Turkey	*Meleagris gallopavo*	Aves	Galliformes	Phasianidae
ENSGALP00000003857	AQP 7	Chicken	*Gallus gallus*	Aves	Galliformes	Phasianidae
AKHW01054970	AQP 7	American alligator	*Alligator mississippiensis*	Archosauria	Crocodylia	Alligatoridae
AVPB01053037	AQP 7	Chinese alligator	*Alligator sinensis*	Archosauria	Crocodylia	Alligatoridae
ENSPSIP00000017416	AQP 7	Chinese softshell turtle	*Pelodiscus sinensis*	Sauropsida	Testudines	Trionychidae
EMP32514	AQP 7	Green seaturtle	*Chelonia mydas*	Sauropsida	Testudines	Cheloniidae
AHGY01159091/AHGY01159090/AHGY01159089	AQP 7	Western painted turtle	*Chrysemys picta bellii*	Sauropsida	Testudines	Emydidae
AZIM01008092/AZIM01023195/AZIM01004676	AQP 7	King cobra	*Ophiophagus hannah*	Lepidosauria	Squamata	Elapidae
AEQU02200793/AEQU02200796/AEQU02069794/AEQU02069792/AEQU02069791	AQP 7	Burmese python	*Python molurus bivittatus*	Lepidosauria	Squamata	Pythonidae
ENSACAP00000012359	AQP 7-1	Green anole	*Anolis carolinensis*	Lepidosauria	Squamata	Iguanidae
ENSACAP00000009259	AQP 7-2	Green anole	*Anolis carolinensis*	Lepidosauria	Squamata	Iguanidae
NP_001015726/ENSXETP00000063272/AAMC02000002/AAMC02000174/AAMC02000175	AQP 7	Western clawed frog	*Xenopus (Silurana) tropicalis*	Amphibia	Anura	Pipidae
GAQK01140589/GAQK01041595	AQP 7	Chinese salamander	*Hynobius chinensis*	Amphibia	Caudata	Hynobiidae
AFYH01154017/AFYH01154016/AFYH01154015/AFYH01154013/AFYH01154012	Aqp 7	Coelacanth	*Latimeria chalumnae*	Actinistia	Coelacanthiformes	Coelacanthidae
GAPS01007864	Aqp 7	Menado coelacanth	*Latimeria menadoensis*	Actinistia	Coelacanthiformes	Coelacanthidae
ENSTRUP00000039522	Aqp 7	Torafugu	*Takifugu rubripes*	Acanthopterygii	Tetraodontiformes	Tetraodontidae
AOOT01048843	Aqp 7	Sansaifugu	*Takifugu flavidus*	Acanthopterygii	Tetraodontiformes	Tetraodontidae
BAL44696	Aqp 7	Mefugu	*Takifugu obscurus*	Acanthopterygii	Tetraodontiformes	Tetraodontidae
ENSTNIP00000013592	Aqp 7	Green-spotted pufferfish	*Tetraodon nigroviridis*	Acanthopterygii	Tetraodontiformes	Tetraodontidae
GAAQ01015072/GAAQ01003666	Aqp 7	Dover sole	*Solea solea*	Acanthopterygii	Pleuronectiformes	Soleidae
AGRG01023824	Aqp 7	Tongue sole	*Cynoglossus semilaevis*	Acanthopterygii	Pleuronectiformes	Cynoglossidae
CBN81126	Aqp 7	European seabass	*Dicentrarchus labrax*	Acanthopterygii	Perciformes	Moronidae
AGTA02009238	Aqp 7	Zebra mbuna	*Maylandia zebra*	Acanthopterygii	Perciformes	Cichlidae
AFNX01015815	Aqp 7	Red mwanza	*Pundamilia nyererei*	Acanthopterygii	Perciformes	Cichlidae
AFNY01011849	Aqp 7	Lyretail cichlid	*Neolamprologus brichardi*	Acanthopterygii	Perciformes	Cichlidae
AFNZ01006222	Aqp 7	Burton's mouthbrooder	*Haplochromis burtoni*	Acanthopterygii	Perciformes	Cichlidae
ENSONIP00000024506	Aqp 7	Nile tilapia	*Oreochromis niloticus*	Acanthopterygii	Perciformes	Cichlidae
BADN01039026	Aqp 7	Pacific bluefin tuna	*Thunnus orientalis*	Acanthopterygii	Perciformes	Scombridae
GAAQ01003666	Aqp 7	Dover sole	*Solea solea*	Acanthopterygii	Pleuronectiformes	Soleidae
GAJJ01041829/GAJJ01041828/GAJJ01041827/AWGY01175109	Aqp 7	Sablefish	*Anoplopoma fimbria*	Acanthopterygii	Scorpaeniformes	Anoplopomatidae
AUPQ01000186	Aqp 7	Flag rockfish	*Sebastes rubrivinctus*	Acanthopterygii	Scorpaeniformes	Sebastidae
AUPR01045372/AUPR01045371	Aqp 7	Tiger rockfish	*Sebastes nigrocinctus*	Acanthopterygii	Scorpaeniformes	Sebastidae
ENSGACP00000011600	Aqp 7	Three-spined stickleback	*Gasterosteus aculeatus*	Acanthopterygii	Gasterosteiformes	Gasterosteidae
ENSORLP00000011130/XP_004068924	Aqp 7	Japanese medaka	*Oryzias latipes*	Acanthopterygii	Beloniformes	Adrianichthyidae
GAIB01101513	Aqp 7	Turquoise killifish	*Nothobranchius furzeri*	Acanthopterygii	Cyprinodontiformes	Nothobranchiidae
DN951591	Aqp 7	Common mummichog	*Fundulus heteroclitus*	Acanthopterygii	Cyprinodontiformes	Fundulidae
ES380999	Aqp 7	Guppy	*Poecilia reticulata*	Acanthopterygii	Cyprinodontiformes	Poeciliidae
AYCK01005653	Aqp 7	Amazon molly	*Poecilia formosa*	Acanthopterygii	Cyprinodontiformes	Poeciliidae
ENSXMAP00000018206	Aqp 7	Southern platyfish	*Xiphophorus maculatus*	Acanthopterygii	Cyprinodontiformes	Poeciliidae
ENSGMOP00000009366	Aqp 7	Atlantic cod	*Gadus morhua*	Paracanthopterygii	Gadiformes	Gadidae
JP753060/JP744328	Aqp 7	Ayu	*Plecoglossus altivelis*	Protacanthopterygii	Osmeriformes	Osmeridae
EZ893726/CCAF010014398	Aqp 7	Rainbow trout	*Oncorhynchus mykiss*	Protacanthopterygii	Salmoniformes	Salmonidae
AGKD01021376/AGKD01033103/AGKD01065234	Aqp 7	Atlantic salmon	*Salmo salar*	Protacanthopterygii	Salmoniformes	Salmonidae
EE396397	Aqp 7	Rare gudgeon	*Gobiocypris rarus*	Ostariophysi	Cypriniformes	Cyprinidae
FJ655385/ENSDARP00000037835	Aqp 7	Zebrafish	*Danio rerio*	Ostariophysi	Cypriniformes	Cyprinidae
APWO01079311	Aqp 7	Mexican tetra	*Astyanax mexicanus*	Ostariophysi	Characiformes	Characidae
ADO28207	Aqp 7	Blue catfish	*Ictalurus furcatus*	Ostariophysi	Siluriformes	Ictaluridae
NP_001188011	Aqp 7	Channel catfish	*Ictalurus punctatus*	Ostariophysi	Siluriformes	Ictaluridae
AVPY01118075/AVPY01485360/AVPY01278193/AVPY01542626	Aqp 7	Japanese eel	*Anguilla japonica*	Elopomorpha	Anguilliformes	Anguillidae
ENSP00000219919	AQP 9	Human	*Homo sapiens*	Euarchontoglires	Primates	Hominidae
ENSPTRP00000012166	AQP 9	Chimpanzee	*Pan troglodytes*	Euarchontoglires	Primates	Hominidae
ENSGGOP00000011853	AQP 9	Western lowland gorilla	*Gorilla gorilla gorilla*	Euarchontoglires	Primates	Hominidae
ENSPPYP00000007384	AQP 9	Sumatran orangutan	*Pongo abelii*	Euarchontoglires	Primates	Hominidae
ENSNLEP00000013765	AQP 9	Northern white-cheeked gibbon	*Nomascus leucogenys*	Euarchontoglires	Primates	Hylobatidae
ENSMMUP00000014712	AQP 9	Rhesus macaque	*Macaca mulatta*	Euarchontoglires	Primates	Cercopithecidae
ENSP00000219919	AQP 9	Hamadryas baboon	*Papio hamadryas*	Euarchontoglires	Primates	Cercopithecidae
AHZZ01026334	AQP 9	Olive baboon	*Papio anubis*	Euarchontoglires	Primates	Cercopithecidae
ENSCJAP00000014666	AQP 9	White-tufted-ear marmoset	*Callithrix jacchus*	Euarchontoglires	Primates	Cebidae
XP_003929026	AQP 9	Bolivian squirrel monkey	*Saimiri boliviensis boliviensis*	Euarchontoglires	Primates	Cebidae
ENSTSYP00000001355	AQP 9	Philippine tarsier	*Tarsius syrichta*	Euarchontoglires	Primates	Tarsiidae
ENSMICP00000005588	AQP 9	Gray mouse lemur	*Microcebus murinus*	Euarchontoglires	Primates	Cheirogaleidae
ENSOGAP00000000433	AQP 9	Small-eared galago/Bushbaby	*Otolemur garnettii*	Euarchontoglires	Primates	Galagidae
ENSTBEP00000002203	AQP 9	Northern tree shrew	*Tupaia belangeri*	Euarchontoglires	Scandentia	Tupaiidae
ELW71355	AQP 9	Chinese tree shrew	*Tupaia chinensis*	Euarchontoglires	Scandentia	Tupaiidae
ENSMUSP00000109200	AQP 9	Mouse	*Mus musculus*	Euarchontoglires	Rodentia	Muridae
ENSRNOP00000021442	AQP 9	Norway rat	*Rattus norvegicus*	Euarchontoglires	Rodentia	Muridae
XP_003495086	AQP 9	Chinese hamster	*Cricetulus griseus*	Euarchontoglires	Rodentia	Cricetidae
ENSDORP00000007120	AQP 9	Ord's kangaroo rat	*Dipodomys ordii*	Euarchontoglires	Rodentia	Heteromyidae
ENSCPOP00000011915/XP_003462107	AQP 9	Domestic guinea pig	*Cavia porcellus*	Euarchontoglires	Rodentia	Caviidae
ENSSTOP00000012659	AQP 9	Thirteen-lined ground squirrel	*Ictidomys tridecemlineatus*	Euarchontoglires	Rodentia	Sciuridae
ENSOPRP00000014273	AQP 9	American pika	*Ochotona princeps*	Euarchontoglires	Lagomorpha	Ochotonidae
ENSOCUP00000008440	AQP 9	Rabbit	*Oryctolagus cuniculus*	Euarchontoglires	Lagomorpha	Leporidae
ENSEEUP00000013310	AQP 6	Western European hedgehog	*Erinaceus europaeus*	Laurasiatheria	Insectivora	Erinaceinae
ENSSARP00000010833	AQP 9	Common shrew	*Sorex araneus*	Laurasiatheria	Insectivora	Soricidae
ENSPVAP00000008897	AQP 9	Large flying fox/Megabat	*Pteropus vampyrus*	Laurasiatheria	Chiroptera	Pteropodidae
ELK05380/ALWS01090306	AQP 9	Black flying fox	*Pteropus alecto*	Laurasiatheria	Chiroptera	Pteropodidae
ALEH01043917	AQP 9-1	Big brown bat	*Eptesicus fuscus*	Laurasiatheria	Chiroptera	Vespertilionidae
ALEH01022282	AQP 9-2	Big brown bat	*Eptesicus fuscus*	Laurasiatheria	Chiroptera	Vespertilionidae
ALEH01006118	AQP 9-3	Big brown bat	*Eptesicus fuscus*	Laurasiatheria	Chiroptera	Vespertilionidae
AAPE02011488/AAPE02011487/ENSMLUP00000001007	AQP 9-1	Little brown bat/Microbat	*Myotis lucifugus*	Laurasiatheria	Chiroptera	Vespertilionidae
AAPE02022705/ENSMLUP00000017800	AQP 9-2	Little brown bat/Microbat	*Myotis lucifugus*	Laurasiatheria	Chiroptera	Vespertilionidae
AAPE02001507/ENSMLUG00000024335	AQP 9-3	Little brown bat/Microbat	*Myotis lucifugus*	Laurasiatheria	Chiroptera	Vespertilionidae
ALWT01117125	AQP 9-1	David´s myotis	*Myotis davidii*	Laurasiatheria	Chiroptera	Vespertilionidae
ALWT01078989	AQP 9-2	David´s myotis	*Myotis davidii*	Laurasiatheria	Chiroptera	Vespertilionidae
ENSVPAP00000010369	AQP 9	Alpaca	*Vicugna pacos*	Laurasiatheria	Tylopoda	Camelidae
ENSBTAP00000017894	AQP 9	Sheep	*Ovis aries*	Laurasiatheria	Ruminantia	Bovidae
ENSBTAP00000017894	AQP 9	Cow	*Bos taurus*	Laurasiatheria	Ruminantia	Bovidae
ELR45536	AQP 9	Yak	*Bos grunniens mutus*	Laurasiatheria	Ruminantia	Bovidae
ANOL02038131	AQP 9	Killer whale	*Orcinus orca*	Laurasiatheria	Cetacea	Delphinidae
ENSTTRP00000004860	AQP 9	Bottlenosed dolphin	*Tursiops truncatus*	Laurasiatheria	Cetacea	Delphinidae
ENSSSCP00000004954	AQP 9	Pig	*Sus scrofa*	Laurasiatheria	Suina	Suidae
EFB23520	AQP 9	Giant panda	*Ailuropoda melanoleuca*	Laurasiatheria	Carnivora	Ursidae

ENSCAFP00000024352	AQP 9	Dog	*Canis lupus familiaris*	Laurasiatheria	Carnivora	Canidae
ENSCAFP00000024352	AQP 9	Domestic ferret	*Mustela putorius furo*	Laurasiatheria	Carnivora	Mustelidae
ENSFCAP00000010263	AQP 9	Domestic cat	*Felis catus*	Laurasiatheria	Carnivora	Felidae
ENSECAP00000010600	AQP 9	Horse	*Equus caballus*	Laurasiatheria	Perissodactyla	Equidae
XP_004421682	AQP 9	Southern white rhinocerus	*Ceratotherium simum simum*	Laurasiatheria	Perissodactyla	Rhinocerotidae
ENSPCAP00000004107	AQP 9	Cape rock hyrax	*Procavia capensis*	Afrotheria	Hyracoidea	Procaviidae
ENSLAFP00000010161	AQP 9	African savanna elephant	*Loxodonta africana*	Afrotheria	Proboscidea	Elephantidae
AHIN01039528	AQP 9	Florida manatee	*Trichechus manatus latirostris*	Afrotheria	Sirenia	Trichechidae
ENSETEP00000000309	AQP 9	Lesser hedgehog tenrec	*Echinops telfairi*	Afrotheria	Afrosoricida	Tenrecidae
ALYB01031433	AQP 9	Ardvaark	*Orycteropus afer afer*	Afrotheria	Tubulidentata	Orycteropodidae
ENSDNOP00000000976	AQP 9	Nine-banded armadillo	*Dasypus novemcinctus*	Xenarthra	Cingulata	Dasypodidae
ENSCHOP00000005421	AQP 9	Hoffmann's two-fingered sloth	*Choloepus hoffmanni*	Xenarthra	Pilosa	Megalonychidae
ENSMEUP00000012468	AQP 9	Tammar wallaby	*Macropus eugenii*	Metatheria	Diprotodontia	Macropodidae
ENSSHAP00000013384	AQP 9	Tasmanian devil	*Sarcophilus harrisii*	Metatheria	Dasyuromorphia	Dasyuridae
ENSMODP00000010777	AQP 9	Gray short-tailed opossum	*Monodelphis domestica*	Metatheria	Didelphimorphia	Didelphidae
ENSOANP00000021925	AQP 9	Platypus	*Ornithorhynchus anatinus*	Prototheria	Monotremata	Ornithorhynchidae
AGAI01054226/AGAI01054227	AQP 9	Budgerigar	*Melopsittacus undulatus*	Aves	Psittaciformes	Psittacidae
AMXX01076578/AMXX01055805/AMXX01021731/ AMXX01068820/AMXX01160063/AOUJ01245559/ AOUJ01394219/AOUJ01082148/AOUJ01278220/ AOUJ01085378	AQP 9	Scarlet macaw	*Ara macao*	Aves	Psittaciformes	Psittacidae
CAVT010016951	AQP 9	Common canary	*Serinus canaria*	Aves	Passeriformes	Fringillidae
AKZB01010303	AQP 9	Medium ground finch	*Geospiza fortis*	Aves	Passeriformes	Fringillidae
ENSTGUP00000006336	AQP 9	Zebra finch	*Taeniopygia guttata*	Aves	Passeriformes	Estrildidae
AGTO01020818	AQP 9	Collared flycatcher	*Ficedula albicollis*	Aves	Passeriformes	Muscicapidae
EMC81258	AQP 9	Rock pigeon	*Columba livia*	Aves	Columbiformes	Columbidae
ENSAPLP00000009910	AQP 9	Mallard	*Anas platyrhynchos*	Aves	Anseriformes	Anatidae
ENSMGAP00000005421	AQP 9	Turkey	*Meleagris gallopavo*	Aves	Galliformes	Phasianidae
ENSGALP00000038464	AQP 9	Chicken	*Gallus gallus*	Aves	Galliformes	Phasianidae
AKHW01033073	AQP 9	American alligator	*Alligator mississippiensis*	Archosauria	Crocodylia	Alligatoridae
AVPB01021124/AVPB01021125	AQP 9	Chinese alligator	*Alligator sinensis*	Archosauria	Crocodylia	Alligatoridae
ENSPSIP00000018757	AQP 9	Chinese softshell turtle	*Pelodiscus sinensis*	Sauropsida	Testudines	Trionychidae
APJP01469951/APJP01460469/APJP01630858	AQP 9	Spiny softshell turtle	*Apalone spinifera*	Sauropsida	Testudines	Trionychidae
EMP38987	AQP 9	Green seaturtle	*Chelonia mydas*	Sauropsida	Testudines	Cheloniidae
AHGY01440703/AHGY01440705/AHGY01440707	AQP 9	Western painted turtle	*Chrysemys picta bellii*	Sauropsida	Testudines	Emydidae
ETE57537/AZIM01000142	AQP 9	King cobra	*Ophiophagus hannah*	Lepidosauria	Squamata	Elapidae
AEQU02169704/AEQU02169705	AQP 9	Burmese python	*Python molurus bivittatus*	Lepidosauria	Squamata	Pythonidae
ENSACAP00000014594	AQP 9	Green anole	*Anolis carolinensis*	Lepidosauria	Squamata	Iguanidae
GAEI01001730	AQP 9	Pacific treefrog	*Pseudacris regilla*	Amphibia	Anura	Hylidae
ENSXETP00000023755	AQP 9	Western clawed frog	*Xenopus (Silurana) tropicalis*	Amphibia	Anura	Pipidae
GAQK01104642	AQP 9	Chinese salamander	*Hynobius chinensis*	Amphibia	Caudata	Hynobiidae
AFYH01095932/AFYH01095933/AFYH01095935/ENS LACP00000022282	Aqp 9	Coelacanth	*Latimeria chalumnae*	Actinistia	Coelacanthiformes	Coelacanthidae
GAPS01056465	Aqp 9	Menado coelacanth	*Latimeria menadoensis*	Actinistia	Coelacanthiformes	Coelacanthidae
ENSTRUP00000021028	Aqp 9a	Torafugu	*Takifugu rubripes*	Acanthopterygii	Tetraodontiformes	Tetraodontidae
AOOT01011453/AOOT01011454/AOOT01011455/A OOT01011456/AOOT01011457	Aqp 9a	Sansaifugu	*Takifugu flavidus*	Acanthopterygii	Tetraodontiformes	Tetraodontidae
ENSTNIP00000016703	Aqp 9a	Green-spotted pufferfish	*Tetraodon nigroviridis*	Acanthopterygii	Tetraodontiformes	Tetraodontidae
AGRG01021247	Aqp 9a	Tongue sole	*Cynoglossus semilaevis*	Acanthopterygii	Pleuronectiformes	Cynoglossidae
AGTA02045424	Aqp 9a	Zebra mbuna	*Maylandia zebra*	Acanthopterygii	Perciformes	Cichlidae
AFNX01011372	Aqp 9a	Red mwanza	*Pundamilia nyererei*	Acanthopterygii	Perciformes	Cichlidae
AFNY01021954	Aqp 9a	Lyretail cichlid	*Neolamprologus brichardi*	Acanthopterygii	Perciformes	Cichlidae
AFNZ01017267	Aqp 9a	Burton's mouthbrooder	*Haplochromis burtoni*	Acanthopterygii	Perciformes	Cichlidae
ENSONIP00000010493	Aqp 9a	Nile tilapia	*Oreochromis niloticus*	Acanthopterygii	Perciformes	Cichlidae
BADN01052475	Aqp 9a	Pacific bluefin tuna	*Thunnus orientalis*	Acanthopterygii	Perciformes	Scombridae
AWGY01074468	Aqp 9a	Sablefish	*Anoplopoma fimbria*	Acanthopterygii	Scorpaeniformes	Anoplopomatidae
AUPQ01002806	Aqp 9a	Flag rockfish	*Sebastes rubrivinctus*	Acanthopterygii	Scorpaeniformes	Sebastidae
AUPR01101625	Aqp 9a	Tiger rockfish	*Sebastes nigrocinctus*	Acanthopterygii	Scorpaeniformes	Sebastidae
ENSGACP00000008112	Aqp 9a	Three-spined stickleback	*Gasterosteus aculeatus*	Acanthopterygii	Gasterosteiformes	Gasterosteidae
BAAE01185479	Aqp 9a	Japanese medaka	*Oryzias latipes*	Acanthopterygii	Beloniformes	Adrianichthyidae
GAIB01121404	Aqp 9a	Turquoise killifish	*Nothobranchius furzeri*	Acanthopterygii	Cyprinodontiformes	Nothobranchiidae
AYCK01018417	Aqp 9a	Amazon molly	*Poecilia formosa*	Acanthopterygii	Cyprinodontiformes	Poeciliidae
ENSXMAP00000006643	Aqp 9a	Southern platyfish	*Xiphophorus maculatus*	Acanthopterygii	Cyprinodontiformes	Poeciliidae
ENSGMOP00000004525	Aqp 9a	Atlantic cod	*Gadus morhua*	Paracanthopterygii	Gadiformes	Gadidae
CCAF010107422	Aqp 9a1	Rainbow trout	*Oncorhynchus mykiss*	Protacanthopterygii	Salmoniformes	Salmonidae
AGKD01252585/AGKD01126390/AGKD01010521	Aqp 9a1	Atlantic salmon	*Salmo salar*	Protacanthopterygii	Salmoniformes	Salmonidae
AGKD01109773/AGKD01265939/AGKD01211321/ AGKD01370165/AGKD01142451	Aqp 9a2	Atlantic salmon	*Salmo salar*	Protacanthopterygii	Salmoniformes	Salmonidae
GAHO01107147/GAHO01051501/GAHO01027437	Aqp 9a	Small gill opening goldenline barbel	*Sinocyclocheilus angustiporus*	Ostariophysi	Cypriniformes	Cyprinidae
GAHL01053586/GAHL01053586	Aqp 9a	Blind goldenline barbel	*Sinocyclocheilus anophthalmus*	Ostariophysi	Cypriniformes	Cyprinidae
FJ655387	Aqp 9a	Zebrafish	*Danio rerio*	Ostariophysi	Cypriniformes	Cyprinidae
APWO01074714	Aqp 9a	Mexican tetra	*Astyanax mexicanus*	Ostariophysi	Characiformes	Characidae
AVPY01019823/AVPY01019824	Aqp 9a	Japanese eel	*Anguilla japonica*	Elopomorpha	Anguilliformes	Anguillidae
ENSTRUP00000045656	Aqp 9b	Torafugu	*Takifugu rubripes*	Acanthopterygii	Tetraodontiformes	Tetraodontidae
AOOT01040226	Aqp 9a	Sansaifugu	*Takifugu flavidus*	Acanthopterygii	Tetraodontiformes	Tetraodontidae
ENSTNIP00000012198	Aqp 9b	Green-spotted pufferfish	*Tetraodon nigroviridis*	Acanthopterygii	Tetraodontiformes	Tetraodontidae
AGRG01025282	Aqp 9b	Tongue sole	*Cynoglossus semilaevis*	Acanthopterygii	Pleuronectiformes	Cynoglossidae
AGTA02006660	Aqp 9b	Zebra mbuna	*Maylandia zebra*	Acanthopterygii	Perciformes	Cichlidae
AFNX01029189	Aqp 9b	Red mwanza	*Pundamilia nyererei*	Acanthopterygii	Perciformes	Cichlidae
AFNY01001740/AFNY01001741	Aqp 9b	Lyretail cichlid	*Neolamprologus brichardi*	Acanthopterygii	Perciformes	Cichlidae
AFNZ01017868/AFNZ01017869	Aqp 9b	Burton's mouthbrooder	*Haplochromis burtoni*	Acanthopterygii	Perciformes	Cichlidae
ENSONIP00000007337	Aqp 9b	Nile tilapia	*Oreochromis niloticus*	Acanthopterygii	Perciformes	Cichlidae
BADN01003195	Aqp 9b	Pacific bluefin tuna	*Thunnus orientalis*	Acanthopterygii	Perciformes	Scombridae
AWGY01022394/AWGY01133405	Aqp 9b	Sablefish	*Anoplopoma fimbria*	Acanthopterygii	Scorpaeniformes	Anoplopomatidae
AUPQ01023380	Aqp 9b	Flag rockfish	*Sebastes rubrivinctus*	Acanthopterygii	Scorpaeniformes	Sebastidae
AUPR01174017	Aqp 9b	Tiger rockfish	*Sebastes nigrocinctus*	Acanthopterygii	Scorpaeniformes	Sebastidae
ENSGACP00000020886	Aqp 9b	Three-spined stickleback	*Gasterosteus aculeatus*	Acanthopterygii	Gasterosteiformes	Gasterosteidae
ENSORLP00000010364	Aqp 9b	Japanese medaka	*Oryzias latipes*	Acanthopterygii	Beloniformes	Adrianichthyidae
GAIB01109759	Aqp 9b	Turquoise killifish	*Nothobranchius furzeri*	Acanthopterygii	Cyprinodontiformes	Nothobranchiidae
AYCK01016323	Aqp 9b	Amazon molly	*Poecilia formosa*	Acanthopterygii	Cyprinodontiformes	Poeciliidae
ENSXMAP00000014456	Aqp 9b	Southern platyfish	*Xiphophorus maculatus*	Acanthopterygii	Cyprinodontiformes	Poeciliidae
ENSGMOP00000016698	Aqp 9b	Atlantic cod	*Gadus morhua*	Paracanthopterygii	Gadiformes	Gadidae
JP742598	Aqp 9b	Ayu	*Plecoglossus altivelis*	Protacanthopterygii	Osmeriformes	Osmeridae
ABG24574	Aqp 9b	Rainbow smelt	*Osmerus mordax*	Protacanthopterygii	Osmeriformes	Osmeridae
CCAF010175523	Aqp 9b1	Rainbow trout	*Oncorhynchus mykiss*	Protacanthopterygii	Salmoniformes	Salmonidae
AGKD01025380/AGKD01043301	Aqp 9b1	Atlantic salmon	*Salmo salar*	Protacanthopterygii	Salmoniformes	Salmonidae
CCAF010134675	Aqp 9b2	Rainbow trout	*Oncorhynchus mykiss*	Protacanthopterygii	Salmoniformes	Salmonidae
AGKD01037951/AGKD01133133/AGKD01006400	Aqp 9b2	Atlantic salmon	*Salmo salar*	Protacanthopterygii	Salmoniformes	Salmonidae
DT167910	Aqp 9b	Fathead minnow	*Pimephales promelas*	Ostariophysi	Cypriniformes	Cyprinidae
GAHO01100067	Aqp 9b	Small gill opening goldenline barbel	*Sinocyclocheilus angustiporus*	Ostariophysi	Cypriniformes	Cyprinidae
GAHL01087848/GAHL01093417	Aqp 9b	Blind goldenline barbel	*Sinocyclocheilus anophthalmus*	Ostariophysi	Cypriniformes	Cyprinidae

EU341835/ENSDARP00000069995	Aqp 9b	Zebrafish	*Danio rerio*	Ostariophysi	Cypriniformes	Cyprinidae
APWO01008619/APWO01008620/APWO01008621	Aqp 9b	Mexican tetra	*Astyanax mexicanus*	Ostariophysi	Characiformes	Characidae
AVPY01127203	Aqp 9b	Japanese eel	*Anguilla japonica*	Elopomorpha	Anguilliformes	Anguillidae
AHAT01024872	Aqp 9	Spotted gar	*Lepisosteus oculatus*	Holostei	Semionotiformes	Lepisosteidae
AESE010685088/AESE011074221/AESE010154666/ AESE011591079	Aqp 9	Little skate	*Leucoraja erinacea*	Chondrichthyes	Rajiformes	Rajidae
AAVX02002482	Aqp 9	Ghost shark	*Callorhinchus milii*	Chondrichthyes	Chimaeriformes	Callorhinchidae
ENSP00000318355	AQP 10	Human	*Homo sapiens*	Euarchontoglires	Primates	Hominidae
ENSPTRP00000044066	AQP 10	Chimpanzee	*Pan troglodytes*	Euarchontoglires	Primates	Hominidae
ENSGGOP00000011599	AQP 10	Western lowland gorilla	*Gorilla gorilla gorilla*	Euarchontoglires	Primates	Hominidae
ENSPPYP00000000910	AQP 10	Sumatran orangutan	*Pongo abelii*	Euarchontoglires	Primates	Hominidae
ENSNLEP00000013741	AQP 10	Northern white-cheeked gibbon	*Nomascus leucogenys*	Euarchontoglires	Primates	Hylobatidae
ENSMMUP00000030755	AQP 10	Rhesus macaque	*Macaca mulatta*	Euarchontoglires	Primates	Cercopithecidae
ENSP00000318355/GENSCAN00000073042	AQP 10	Hamadryas baboon	*Papio hamadryas*	Euarchontoglires	Primates	Cercopithecidae
AHZZ01032599	AQP 10	Olive baboon	*Papio anubis*	Euarchontoglires	Primates	Cercopithecidae
ENSCJAP00000017692	AQP 10	White-tufted-ear marmoset	*Callithrix jacchus*	Euarchontoglires	Primates	Cebidae
XP_003941910	AQP 10	Bolivian squirrel monkey	*Saimiri boliviensis boliviensis*	Euarchontoglires	Primates	Cebidae
ENSMICP00000003203	AQP 10	Gray mouse lemur	*Microcebus murinus*	Euarchontoglires	Primates	Cheirogaleidae
ENSOGAP00000004925	AQP 10	Small-eared galago/Bushbaby	*Otolemur garnettii*	Euarchontoglires	Primates	Galagidae
ENSTBEP00000002048	AQP 10	Northern tree shrew	*Tupaia belangeri*	Euarchontoglires	Scandentia	Tupaiidae
ALAR01116048	AQP 10	Chinese tree shrew	*Tupaia chinensis*	Euarchontoglires	Scandentia	Tupaiidae
AEKR01029399	AQP 10	Mouse	*Mus musculus*	Euarchontoglires	Rodentia	Muridae
AAHX01018619	AQP 10	Norway rat	*Rattus norvegicus*	Euarchontoglires	Rodentia	Muridae
XP_003501706	AQP 10	Chinese hamster	*Cricetulus griseus*	Euarchontoglires	Rodentia	Cricetidae
ENSDORP00000008117	AQP 10	Ord's kangaroo rat	*Dipodomys ordii*	Euarchontoglires	Rodentia	Heteromyidae
AAKN02015643	AQP 10	Domestic guinea pig	*Cavia porcellus*	Euarchontoglires	Rodentia	Caviidae
EHB07289	AQP 10	Naked mole-rat	*Heterocephalus glaber*	Euarchontoglires	Rodentia	Bathyergidae
ENSSTOP00000013093	AQP 10	Thirteen-lined ground squirrel	*Ictidomys tridecemlineatus*	Euarchontoglires	Rodentia	Sciuridae
ENSOPRP00000011430	AQP 10	American pika	*Ochotona princeps*	Euarchontoglires	Lagomorpha	Ochotonidae
ENSOCUP00000014024	AQP 10	Rabbit	*Oryctolagus cuniculus*	Euarchontoglires	Lagomorpha	Leporidae
AANN01083412/AANN01311780/AMDU01107128	AQP 10	Western European hedgehog	*Erinaceus europaeus*	Laurasiatheria	Insectivora	Erinaceinae
ENSSARP00000008216	AQP 10	Common shrew	*Sorex araneus*	Laurasiatheria	Insectivora	Soricidae
ENSPVAP00000002580	AQP 10	Large flying fox/Megabat	*Pteropus vampyrus*	Laurasiatheria	Chiroptera	Pteropodidae
ELK02825	AQP 10	Black flying fox	*Pteropus alecto*	Laurasiatheria	Chiroptera	Pteropodidae
ENSMLUP00000007752	AQP 10	Little brown bat/Microbat	*Myotis lucifugus*	Laurasiatheria	Chiroptera	Vespertilionidae
ENSP00000318355/AMGL01004792	AQP 10	Sheep	*Ovis aries*	Laurasiatheria	Ruminantia	Bovidae
XP_003581985	AQP 10	Cow	*Bos taurus*	Laurasiatheria	Ruminantia	Bovidae
AGSK01099059	AQP 10	Yak	*Bos grunniens mutus*	Laurasiatheria	Ruminantia	Bovidae
ANOL02071573	AQP 10	Killer whale	*Orcinus orca*	Laurasiatheria	Cetacea	Delphinidae
ENSTTRP00000015030	AQP 10	Bottlenosed dolphin	*Tursiops truncatus*	Laurasiatheria	Cetacea	Delphinidae
ENSSSCP00000006983	AQP 10	Pig	*Sus scrofa*	Laurasiatheria	Suina	Suidae
ENSAMEP00000012346	AQP 10	Giant panda	*Ailuropoda melanoleuca*	Laurasiatheria	Carnivora	Ursidae
ENSCAFP00000025344	AQP 10	Dog	*Canis lupus familiaris*	Laurasiatheria	Carnivora	Canidae
ENSCAFP00000025344	AQP 10	Domestic ferret	*Mustela putorius furo*	Laurasiatheria	Carnivora	Mustelidae
ENSFCAP00000006561	AQP 10	Domestic cat	*Felis catus*	Laurasiatheria	Carnivora	Felidae
JU292194	AQP 10	Spotted seal	*Phoca largha*	Laurasiatheria	Carnivora	Felidae
XP_001494035	AQP 10	Horse	*Equus caballus*	Laurasiatheria	Perissodactyla	Equidae
ABRQ01433123	AQP 10	Cape rock hyrax	*Procavia capensis*	Afrotheria	Hyracoidea	Procaviidae
ENSLAFP00000011791	AQP 10	African savanna elephant	*Loxodonta africana*	Afrotheria	Proboscidea	Elephantidae
AHIN01132419	AQP 10	Florida manatee	*Trichechus manatus latirostris*	Afrotheria	Sirenia	Trichechidae
AAIY02236968	AQP 10	Lesser hedgehog tenrec	*Echinops telfairi*	Afrotheria	Afrosoricida	Tenrecidae
ALYB01202654/ALYB01202653	AQP 10	Ardvaark	*Orycteropus afer afer*	Afrotheria	Tubulidentata	Orycteropodidae
AAGV03212996	AQP 10	Nine-banded armadillo	*Dasypus novemcinctus*	Xenarthra	Cingulata	Dasypodidae
ENSCHOP00000011475	AQP 10	Hoffmann's two-fingered sloth	*Choloepus hoffmanni*	Xenarthra	Pilosa	Megalonychidae
ENSMEUP00000001059	AQP 10	Tammar wallaby	*Macropus eugenii*	Metatheria	Diprotodontia	Macropodidae
AEFK01161835	AQP 10	Tasmanian devil	*Sarcophilus harrisii*	Metatheria	Dasyuromorphia	Dasyuridae
DY589380	AQP 10	Common brushtail	*Trichosurus vulpecula*	Metatheria	Diprotodontia	Phalangeridae
ENSMODP00000021447	AQP 10	Gray short-tailed opossum	*Monodelphis domestica*	Metatheria	Didelphimorphia	Didelphidae
AGAI01011169	AQP 10	Budgerigar	*Melopsittacus undulatus*	Aves	Psittaciformes	Psittacidae
AMXX01268820/AMXX01186663/AOUJ01266258	AQP 10	Scarlet macaw	*Ara macao*	Aves	Psittaciformes	Psittacidae
CAVT010044489/CAVT010044488	AQP 10	Common canary	*Serinus canaria*	Aves	Passeriformes	Fringillidae
AKZB01051931	AQP 10	Medium ground finch	*Geospiza fortis*	Aves	Passeriformes	Fringillidae
XP_002188060	AQP 10	Zebra finch	*Taeniopygia guttata*	Aves	Passeriformes	Estrildidae
ANZD01004762	AQP 10	Tibetan ground-tit	*Pseudopodoces humilis*	Aves	Passeriformes	Paridae
AGTO01012145	AQP 10	Collared flycatcher	*Ficedula albicollis*	Aves	Passeriformes	Muscicapidae
ENSMGAP00000013807	AQP 10	Turkey	*Meleagris gallopavo*	Aves	Galliformes	Phasianidae
AKHW01034781	AQP 10	American alligator	*Alligator mississippiensis*	Archosauria	Crocodylia	Alligatoridae
AVPB01053647/AVPB01053648	AQP 10	Chinese alligator	*Alligator sinensis*	Archosauria	Crocodylia	Alligatoridae
AZIM01117632/AZIM01026944/AZIM01010803/ AZIM01034621	AQP 10	King cobra	*Ophiophagus hannah*	Lepidosauria	Squamata	Elapidae
AEQU02076485/AEQU02076486	AQP 10	Burmese python	*Python molurus bivittatus*	Lepidosauria	Squamata	Pythonidae
ENSACAP00000011333	AQP 10	Green anole	*Anolis carolinensis*	Lepidosauria	Squamata	Iguanidae
GAEG01016981	AQP 10	Green frog	*Rana clamitans*	Amphibia	Anura	Ranidae
ENSXETP00000049658	AQP 10	Western clawed frog	*Xenopus (Silurana) tropicalis*	Amphibia	Anura	Pipidae
GAQK01144279	AQP 10	Chinese salamander	*Hynobius chinensis*	Amphibia	Caudata	Hynobiidae
AFYH01073904/AFYH01073906/AFYH01073908	Aqp 10	Coelacanth	*Latimeria chalumnae*	Actinistia	Coelacanthiformes	Coelacanthidae
GAPS01044178	Aqp 10	Menado coelacanth	*Latimeria menadoensis*	Actinistia	Coelacanthiformes	Coelacanthidae
AOOT01054180	Aqp 10a	Sansaifugu	*Takifugu flavidus*	Acanthopterygii	Tetraodontiformes	Tetraodontidae
ENSTNIP00000021508	Aqp 10aa	Green-spotted pufferfish	*Tetraodon nigroviridis*	Acanthopterygii	Tetraodontiformes	Tetraodontidae
ENSTNIP00000021507	Aqp 10ab	Green-spotted pufferfish	*Tetraodon nigroviridis*	Acanthopterygii	Tetraodontiformes	Tetraodontidae
AGRG01014417	Aqp 10a	Tongue sole	*Cynoglossus semilaevis*	Acanthopterygii	Pleuronectiformes	Cynoglossidae
AGTA02004822/AGTA02004823	Aqp 10a	Zebra mbuna	*Maylandia zebra*	Acanthopterygii	Perciformes	Cichlidae
AFNX01016620	Aqp 10a	Red mwanza	*Pundamilia nyererei*	Acanthopterygii	Perciformes	Cichlidae
AFNY01078024/AFNY01078025	Aqp 10a	Lyretail cichlid	*Neolamprologus brichardi*	Acanthopterygii	Perciformes	Cichlidae
AFNZ01026154	Aqp 10a	Burton's mouthbrooder	*Haplochromis burtoni*	Acanthopterygii	Perciformes	Cichlidae
ENSONIP00000007893	Aqp 10a	Nile tilapia	*Oreochromis niloticus*	Acanthopterygii	Perciformes	Cichlidae
BADN01024342/BADN01024341	Aqp 10a	Pacific bluefin tuna	*Thunnus orientalis*	Acanthopterygii	Perciformes	Scombridae
AWGY01171536	Aqp 10a	Sablefish	*Anoplopoma fimbria*	Acanthopterygii	Scorpaeniformes	Anoplopomatidae
AUPQ01000856/AUPQ01018903	Aqp 10a	Flag rockfish	*Sebastes rubrivinctus*	Acanthopterygii	Scorpaeniformes	Sebastidae
AUPR01022718/AUPR01022717	Aqp 10a	Tiger rockfish	*Sebastes nigrocinctus*	Acanthopterygii	Scorpaeniformes	Sebastidae
ENSGACP00000016074	Aqp 10a	Three-spined stickleback	*Gasterosteus aculeatus*	Acanthopterygii	Gasterosteiformes	Gasterosteidae
ENSORLP00000012050	Aqp 10a	Japanese medaka	*Oryzias latipes*	Acanthopterygii	Beloniformes	Adrianichthyidae
GAIB01200719	Aqp 10a	Turquoise killifish	*Nothobranchius furzeri*	Acanthopterygii	Cyprinodontiformes	Nothobranchiidae
ENSXMAP00000007174	Aqp 10aa	Southern platyfish	*Xiphophorus maculatus*	Acanthopterygii	Cyprinodontiformes	Poeciliidae
AYCK01006466	Aqp 10aa	Amazon molly	*Poecilia formosa*	Acanthopterygii	Cyprinodontiformes	Poeciliidae
ENSXMAP00000007179	Aqp 10ab	Southern platyfish	*Xiphophorus maculatus*	Acanthopterygii	Cyprinodontiformes	Poeciliidae
AYCK01006466	Aqp 10ab	Amazon molly	*Poecilia formosa*	Acanthopterygii	Cyprinodontiformes	Poeciliidae
ENSGMOP00000008513	Aqp 10a	Atlantic cod	*Gadus morhua*	Paracanthopterygii	Gadiformes	Gadidae
CCAF010191786/CCAF010155489	Aqp 10a1	Rainbow trout	*Oncorhynchus mykiss*	Protacanthopterygii	Salmoniformes	Salmonidae
AGKD01044249	Aqp 10a1	Atlantic salmon	*Salmo salar*	Protacanthopterygii	Salmoniformes	Salmonidae

AGKD01200314	Aqp 10a2	Atlantic salmon	*Salmo salar*	Protacanthopterygii	Salmoniformes	Salmonidae
EW688162	Aqp 10a	Grass carp	*Ctenopharyngodon idella*	Ostariophysi	Cypriniformes	Cyprinidae
FJ655388/ENSDARP00000012559	Aqp 10a	Zebrafish	*Danio rerio*	Ostariophysi	Cypriniformes	Cyprinidae
APWO01105564	Aqp 10a	Mexican tetra	*Astyanax mexicanus*	Ostariophysi	Characiformes	Characidae
CK402696	Aqp 10a	Blue catfish	*Ictalurus furcatus*	Ostariophysi	Siluriformes	Ictaluridae
FD105608	Aqp 10a	Channel catfish	*Ictalurus punctatus*	Ostariophysi	Siluriformes	Ictaluridae
AVPY01517596/AVPY01517595/AVPY01161649	Aqp 10a	Japanese eel	*Anguilla japonica*	Elopomorpha	Anguilliformes	Anguillidae
ENSTRUP00000024382	Aqp 10b	Torafugu	*Takifugu rubripes*	Acanthopterygii	Tetraodontiformes	Tetraodontidae
AOOT01088343/AOOT01088344/AOOT01088345/AOOT01088346	Aqp 10b	Sansaifugu	*Takifugu flavidus*	Acanthopterygii	Tetraodontiformes	Tetraodontidae
ENSTNIP00000020714	Aqp 10b	Green-spotted pufferfish	*Tetraodon nigroviridis*	Acanthopterygii	Tetraodontiformes	Tetraodontidae
AGRG01015696	Aqp 10b	Tongue sole	*Cynoglossus semilaevis*	Acanthopterygii	Pleuronectiformes	Cynoglossidae
AAR13054	Aqp 10b	Gilthead seabream	*Sparus aurata*	Acanthopterygii	Perciformes	Sparidae
AGTA02013872	Aqp 10b	Zebra mbuna	*Maylandia zebra*	Acanthopterygii	Perciformes	Cichlidae
AFNX01027601	Aqp 10b	Red mwanza	*Pundamilia nyererei*	Acanthopterygii	Perciformes	Cichlidae
AFNY01020099	Aqp 10b	Lyretail cichlid	*Neolamprologus brichardi*	Acanthopterygii	Perciformes	Cichlidae
AFNZ01015334	Aqp 10b	Burton's mouthbrooder	*Haplochromis burtoni*	Acanthopterygii	Perciformes	Cichlidae
ENSONIP00000024860	Aqp 10b	Nile tilapia	*Oreochromis niloticus*	Acanthopterygii	Perciformes	Cichlidae
DQ889224	Aqp 10b	European seabass	*Dicentrarchus labrax*	Acanthopterygii	Perciformes	Moronidae
BADN01043465/BADN01043464	Aqp 10b	Pacific bluefin tuna	*Thunnus orientalis*	Acanthopterygii	Perciformes	Scombridae
ACQ58348/AWGY01169699	Aqp 10b	Sablefish	*Anoplopoma fimbria*	Acanthopterygii	Scorpaeniformes	Anoplopomatidae
AUPQ01078292	Aqp 10b	Flag rockfish	*Sebastes rubrivinctus*	Acanthopterygii	Scorpaeniformes	Sebastidae
AUPR01011084	Aqp 10b	Tiger rockfish	*Sebastes nigrocinctus*	Acanthopterygii	Scorpaeniformes	Sebastidae
ENSGACP00000005841	Aqp 10b	Three-spined stickleback	*Gasterosteus aculeatus*	Acanthopterygii	Gasterosteiformes	Gasterosteidae
AUPQ01078292	Aqp 10b	Flag rockfish	*Sebastes rubrivinctus*	Acanthopterygii	Scorpaeniformes	Sebastidae
ENSORLP00000000413	Aqp 10b	Japanese medaka	*Oryzias latipes*	Acanthopterygii	Beloniformes	Adrianichthyidae
GAIB01023516	Aqp 10b	Turquoise killifish	*Nothobranchius furzeri*	Acanthopterygii	Cyprinodontiformes	Nothobranchiidae
AYCK01023622	Aqp 10b	Amazon molly	*Poecilia formosa*	Acanthopterygii	Cyprinodontiformes	Poeciliidae
ENSXMAP00000015292	Aqp 10b	Southern platyfish	*Xiphophorus maculatus*	Acanthopterygii	Cyprinodontiformes	Poeciliidae
ENSGMOP00000011541	Aqp 10b	Atlantic cod	*Gadus morhua*	Paracanthopterygii	Gadiformes	Gadidae
EL537770	Aqp 10b	Rainbow smelt	*Osmerus mordax*	Protacanthopterygii	Osmeriformes	Osmeridae
BX910627/CCAF010001940	Aqp 10b1	Rainbow trout	*Oncorhynchus mykiss*	Protacanthopterygii	Salmoniformes	Salmonidae
AGKD01090696	Aqp 10b1	Atlantic salmon	*Salmo salar*	Protacanthopterygii	Salmoniformes	Salmonidae
AGKD01157343	Aqp 10b2	Atlantic salmon	*Salmo salar*	Protacanthopterygii	Salmoniformes	Salmonidae
EV249895	Aqp 10b	Lake whitefish	*Coregonus clupeaformis*	Protacanthopterygii	Salmoniformes	Salmonidae
DT173352	Aqp 10b	Fathead minnow	*Pimephales promelas*	Ostariophysi	Cypriniformes	Cyprinidae
EU341836/ENSDARP00000076033	Aqp 10b	Zebrafish	*Danio rerio*	Ostariophysi	Cypriniformes	Cyprinidae
APWO01008903	Aqp 10b	Mexican tetra	*Astyanax mexicanus*	Ostariophysi	Characiformes	Characidae
FD177421	Aqp 10b	Channel catfish	*Ictalurus punctatus*	Ostariophysi	Siluriformes	Ictaluridae
CAH04573	Aqp 10b	European eel	*Anguilla anguilla*	Elopomorpha	Anguilliformes	Anguillidae
BAH89255/AVPY01266395/AVPY01294745/AVPY01294744/ AVPY01294743/AVPY01779473	Aqp 10b1	Japanese eel	*Anguilla japonica*	Elopomorpha	Anguilliformes	Anguillidae
AVPY01294751/AVPY01266384/AVPY01266386/AVPY01266387/AVPY01266388/ AVPY01753058	Aqp 10b2	Japanese eel	*Anguilla japonica*	Elopomorpha	Anguilliformes	Anguillidae
AHAT01020045/AHAT01020044/AHAT01020043/ENSLOCP00000009159	Aqp 10	Spotted gar	*Lepisosteus oculatus*	Holostei	Semionotiformes	Lepisosteidae
AHAT01020047/AHAT01020046/GENSCAN00000012520	Aqp 10L	Spotted gar	*Lepisosteus oculatus*	Holostei	Semionotiformes	Lepisosteidae
AESE011698308/AESE011634621/AESE011756722/AESE011256220/AESE010860518	Aqp 10	Little skate	*Leucoraja erinacea*	Chondrichthyes	Rajiformes	Rajidae
AAVX02008359	Aqp 10	Ghost shark	*Callorhinchus milii*	Chondrichthyes	Chimaeriformes	Callorhinchidae
ENSPMAP00000008286/AEFG01045232	Aqp 10L1	Sea lamprey	*Petromyzon marinus*	Hyperoartia	Petromyzontiformes	Petromyzontidae
APJL01010233	Aqp 10L1	Arctic lamprey	*Lethenteron camtschaticum*	Hyperoartia	Petromyzontiformes	Petromyzontidae
ENSPMAP00000003207/AEFG01052647	Aqp 10L2	Sea lamprey	*Petromyzon marinus*	Hyperoartia	Petromyzontiformes	Petromyzontidae
APJL01010233	Aqp 10L2	Arctic lamprey	*Lethenteron camtschaticum*	Hyperoartia	Petromyzontiformes	Petromyzontidae
ENSOANP00000015441/XP_001520638	AQP 13	Platypus	*Ornithorhynchus anatinus*	Prototheria	Monotremata	Ornithorhynchidae
NP_001082310/AAM81580	AQP 13	African clawed frog	*Xenopus laevis*	Amphibia	Anura	Pipidae
ENSXETP00000035583/AAMC02034374	AQP 13	Western clawed frog	*Xenopus (Silurana) tropicalis*	Amphibia	Anura	Pipidae
GAEI01021410/GAEI01008988	AQP 13	Pacific treefrog	*Pseudacris regilla*	Amphibia	Anura	Hylidae
GAEG01041111/GAEG01028508	AQP 13	Green frog	*Rana clamitans*	Amphibia	Anura	Ranidae
FS313448	AQP 13	Japanese firebelly newt	*Cynops pyrrhogaster*	Amphibia	Caudata	Salamandridae
GAQK01049134	AQP 13	Chinese salamander	*Hynobius chinensis*	Amphibia	Caudata	Hynobiidae
JV201948	AQP 13	Axolotl	*Ambystoma mexicanum*	Amphibia	Caudata	Ambystomatidae
ENSCINP00000019491	Glp	Vase tunicate	*Ciona intestinalis*	Tunicata	Enterogona	Cionidae
SINCSAVP00000010319	Glp	Pacific transparent sea squirt	*Ciona savignyi*	Tunicata	Enterogona	Cionidae
XP_002604987	Glp	Florida lancelet	*Branchiostoma floridae*	Cephalochordata	Amphioxiformes	Branchiostomidae
XP_002609960	Glp	Florida lancelet	*Branchiostoma floridae*	Cephalochordata	Amphioxiformes	Branchiostomidae
FN991824	Glp	Pterobranch	*Rhabdopleura compacta*	Hemichordata	Rhabdopleurida	Rhabdopleuridae
XP_002736840	Glp	Acorn worm	*Saccoglossus kowalevskii*	Hemichordata	Enteropneusta	Harrimaniidae
AAGJ04123740/AAGJ04123724/AAGJ04123722/XP_786125/SPU_024384_tr	Glp 3	Purple sea urchin	*Strongylocentrotus purpuratus*	Echinodermata	Echinozoa	Strongylocentrotidae
GAPB01003116	Glp 3	Kina	*Evechinus chloroticus*	Echinodermata	Echinozoa	Echinometridae
AGCV01322870/AGCV01012909/AGCV01012912	Glp 3	Green sea urchin	*Lytechinus variegatus*	Echinodermata	Echinozoa	Toxopneustidae
AKZP01133424/AKZP01133423	Glp 3	Bat star	*Patiria miniata*	Echinodermata	Asterozoa	Asterinidae
AAGJ04114907/XP_789770/SPU_004511_tr	Glp 2	Purple sea urchin	*Strongylocentrotus purpuratus*	Echinodermata	Echinozoa	Strongylocentrotidae
GAPB01012573	Glp 2	Kina	*Evechinus chloroticus*	Echinodermata	Echinozoa	Echinometridae
AGCV01043128	Glp 2	Green sea urchin	*Lytechinus variegatus*	Echinodermata	Echinozoa	Toxopneustidae
AKZP01130689/AKZP01138585	Glp 2	Bat star	*Patiria miniata*	Echinodermata	Asterozoa	Asterinidae
AAGJ04134789/AAGJ04134789/XP_796513	Glp 1b	Purple sea urchin	*Strongylocentrotus purpuratus*	Echinodermata	Echinozoa	Strongylocentrotidae
AAGJ04062807/AAGJ04062804/AAGJ04062803/AAGJ04062801/SPU_023979_tr	Glp 1	Purple sea urchin	*Strongylocentrotus purpuratus*	Echinodermata	Echinozoa	Strongylocentrotidae
GAPB01053568	Glp 1	Kina	*Evechinus chloroticus*	Echinodermata	Echinozoa	Echinometridae
AGCV01341974/AGCV01341972/AGCV01341971/AGCV01395938/AGCV01395937	Glp 1	Green sea urchin	*Lytechinus variegatus*	Echinodermata	Echinozoa	Toxopneustidae
AKZP01128013/AKZP01128014/AKZP01038911	Glp 1a	Bat star	*Patiria miniata*	Echinodermata	Asterozoa	Asterinidae
AKZP01008734	Glp 1b	Bat star	*Patiria miniata*	Echinodermata	Asterozoa	Asterinidae
AKZP01008737/AKZP01008738	Glp 1c	Bat star	*Patiria miniata*	Echinodermata	Asterozoa	Asterinidae

Protostomia aquaglyceroporins

Arthropoda aquaglyceroporins

XP_002430403	Glp	Human body louse	*Pediculus humanus corporis*	Hexapoda	Phthiraptera	Pediculidae
GAWR01093456	Glp	Poultry shaft louse	*Menopon gallinae*	Hexapoda	Phthiraptera	Menoponidae
GAYV01015247	Glp 1	Booklice	*Liposcelis bostrychophila*	Hexapoda	Psocoptera	Liposcelidae
GAYV01015248	Glp 2	Booklice	*Liposcelis bostrychophila*	Hexapoda	Psocoptera	Liposcelidae
GAPT01019852	Glp	Booklice	*Ectopsocus briggsi*	Hexapoda	Psocoptera	Ectopsocidae
GAYA01037781	Glp	Zoraptid	*Zorotypus caudelli*	Hexapoda	Orthoptera	Zorotypidae
GABA01000698	Glp	Zoraptid	*Zorotypus gurneyi*	Hexapoda	Zoraptera	Zorotypidae
GAYQ01177068	Glp	European earwig	*Forficula auricularia*	Hexapoda	Dermaptera	Forficulidae
GAYL01081166	Glp	Stonefly	*Cosmioperla kuna*	Hexapoda	Plecoptera	Eustheniidae
GAUF01080011	Glp	Leuctra	*Leuctra sp.*	Hexapoda	Plecoptera	Leuctridae
GATV01110784	Glp	Stonefly	*Perla marginata*	Hexapoda	Plecoptera	Perlidae
GAWU01218234	Glp	Webspinner	*Aposthonia japonica*	Hexapoda	Embioptera	Oligotomidae
GAWG01068929	Glp	Giant prickly stick insect	*Extatosoma tiaratum*	Hexapoda	Phasmatodea	Phasmatidae

GAWE01083837/GAWE01083816	Glp	Vietnamese walking stick insect	*Ramulus artemis*	Hexapoda	Phasmatodea	Phasmatidae
GAWD01050986	Glp	Vietnamese walking stick	*Medauroidea extradentata*	Hexapoda	Phasmatodea	Phasmatidae
GAWF01020399/GAWF01096573/GAWF01024650/ GAWF01024651	Glp	Pink winged stick insect	*Sipyloidea sipylus*	Hexapoda	Phasmatodea	Diapheromeridae
GAWC01056095	Glp	Thorny stick insect	*Aretaon asperrimus*	Hexapoda	Phasmatodea	Heteropterygidae
GAXB01138367	Glp	Heelwalker	*Tanzaniophasma sp.*	Hexapoda	Mantophasmatodea	Tanzaniophasmatidae
GAUX01277991	Glp	Camel cricket	*Ceuthophilus sp.*	Hexapoda	Orthoptera	Rhaphidophoridae
GAZT01142164	Glp	False stick insect	*Prosarthria teretrirostris*	Hexapoda	Orthoptera	Proscopiidae
GASQ01015062	Glp	Slender Groundhopper	*Tetrix subulata*	Hexapoda	Orthoptera	Tetrigidae
GAUZ01006460	Glp	Stripe-winged grasshopper	*Stenobothrus lineatus*	Hexapoda	Orthoptera	Acrididae
GATU01072356	Glp	Blue-winged olive	*Baetis sp.*	Hexapoda	Ephemeroptera	Baetidae
GAXA01113250	Glp	Mahogany Dun	*Isonychia bicolor*	Hexapoda	Ephemeroptera	Isonychiidae
GAZG01101517	Glp	Mayfly	*Eurylophella sp.*	Hexapoda	Ephemeroptera	Ephemerellidae
AYNC01028228/AYNC01028227	Glp	Green drake	*Ephemera danica*	Hexapoda	Ephemeroptera	Ephemeridae
GAYM01097963	Glp	Banded damoiselle	*Calopteryx splendens*	Hexapoda	Odonata	Calopterygidae
GAVW01126922	Glp	Dragonfly	*Epiophlebia superstes*	Hexapoda	Odonata	Epiophlebiidae
GAYO01012540	Glp	Golden-ringed dragonfly	*Cordulegaster boltonii*	Hexapoda	Odonata	Cordulegastridae
APVN01141542/APVN01141543/APVN01141545	Glp	Scarce chaser	*Ladona fulva*	Hexapoda	Odonata	Libellulidae
GASN01018603	Glp 1	Firebrat	*Thermobia domestica*	Hexapoda	Zygentoma	Lepismatidae
GASN01397590	Glp 2	Firebrat	*Thermobia domestica*	Hexapoda	Zygentoma	Lepismatidae
GAYJ01031386	Glp	Silverfish	*Atelura formicaria*	Hexapoda	Zygentoma	Nicoletiidae
GASO01018965	Glp	Silverfish	*Tricholepidion gertschi*	Hexapoda	Zygentoma	Libellulidae
GAUM01020992	Glp	Bristletail	*Machilis hrabei*	Hexapoda	Archaeognatha	Machilidae
GAUG01249683	Glp	Bristletail	*Meinertellus cundinamarcensis*	Hexapoda	Archaeognatha	Meinertellidae
GAYN01134376	Glp 1	Campodea	*Campodea augens*	Hexapoda	Diplura	Campodeidae
GAYN01140084	Glp 2	Campodea	*Campodea augens*	Hexapoda	Diplura	Campodeidae
JT051615	Glp 1	Two-pronged bristletail	*Megajapyx sp.*	Hexapoda	Diplura	Japygidae
GAXJ01112653	Glp 1	Two-pronged bristletail	*Occasjapyx japonicus*	Hexapoda	Diplura	Japygidae
GAXJ01013070	Glp 2	Two-pronged bristletail	*Occasjapyx japonicus*	Hexapoda	Diplura	Japygidae
GATZ01009105	Glp 1	Clover springtail	*Sminthurus viridis*	Hexapoda	Collembola	Sminthuridae
GATZ01103685	Glp 2	Clover springtail	*Sminthurus viridis*	Hexapoda	Collembola	Sminthuridae
GAUE01002450	Glp 1	Cosmopolitan springtail	*Anurida maritima*	Hexapoda	Collembola	Neanuridae
GAUE01007494	Glp 2	Cosmopolitan springtail	*Anurida maritima*	Hexapoda	Collembola	Neanuridae
GAXI01021746	Glp 1	Giant springtail	*Tetrodontophora bielanensis*	Hexapoda	Collembola	Onychiuridae
GAXI01021160	Glp 2	Giant springtail	*Tetrodontophora bielanensis*	Hexapoda	Collembola	Onychiuridae
GAMM01000769	Glp	Springtail	*Orchesella cincta*	Hexapoda	Collembola	Entomobryidae
GAMN01001199	Glp 1	Springtail	*Folsomia candida*	Hexapoda	Collembola	Isotomidae
GAMN01014673	Glp 2	Springtail	*Folsomia candida*	Hexapoda	Collembola	Isotomidae
GATD01099253	Glp	Springtail	*Pogonognathellus sp.*	Hexapoda	Collembola	Tomoceridae
GAXE01023694	Glp	Conehead	*Acerentomon sp.*	Hexapoda	Protura	Acerentomidae
AFFK01019422/SMAR004742	Glp	Coastal European centipede	*Strigamia maritima*	Chilopoda	Geophilomorpha	Linotaeniidae
GAFS01005819	Glp	Narrow-clawed crayfish	*Pontastacus leptodactylus*	Malacostraca	Decapoda	Astacidae
JP360962/JP360767	Glp	Pacific white shrimp	*Litopenaeus vannamei*	Malacostraca	Decapoda	Penaeidae
JR713575	Glp	Chinese mitten crab	*Callinectes sapidus*	Malacostraca	Decapoda	Varunidae
FE813068/FE813069	Glp	Flat porcelain crab	*Petrolisthes cinctipes*	Malacostraca	Decapoda	Porcellanidae
GAKD01001157	Glp	Amphipod	*Melita plumulosa*	Malacostraca	Amphipoda	Melitidae
GARO01026521	Glp	Amphipod	*Echinogammarus veneris*	Malacostraca	Amphipoda	Gammaridae
GAZX01003450	Glp	Caligid copepod	*Caligus rogercresseyi*	Maxillopoda	Siphonostomatoida	Caligidae
GAZX01018815	Glp 3	Caligid copepod	*Caligus rogercresseyi*	Maxillopoda	Siphonostomatoida	Caligidae
GAXK01070720	Glp 1a	Calanus	*Calanus finmarchicus*	Maxillopoda	Calanoida	Calanidae
GAXK01165253	Glp 1b	Calanus	*Calanus finmarchicus*	Maxillopoda	Calanoida	Calanidae
GAXK01179438	Glp 1c	Calanus	*Calanus finmarchicus*	Maxillopoda	Calanoida	Calanidae
GAXK01155600	Glp 3a	Calanus	*Calanus finmarchicus*	Maxillopoda	Calanoida	Calanidae
GAXK01155595	Glp 3b	Calanus	*Calanus finmarchicus*	Maxillopoda	Calanoida	Calanidae
JV199638	Glp 1a	Tide pool copepod	*Tigriopus californicus*	Maxillopoda	Harpacticoida	Harpacticidae
JV198720	Glp 1b	Tide pool copepod	*Tigriopus californicus*	Maxillopoda	Harpacticoida	Harpacticidae
JV199221	Glp 3a	Tide pool copepod	*Tigriopus californicus*	Maxillopoda	Harpacticoida	Harpacticidae
JV193385	Glp 3b	Tide pool copepod	*Tigriopus californicus*	Maxillopoda	Harpacticoida	Harpacticidae
GARW01015498	Glp 1	Cyclopid copepod	*Eucyclops serrulatus*	Maxillopoda	Cyclopoida	Cyclopidae
GARW01029074	Glp 3	Cyclopid copepod	*Eucyclops serrulatus*	Maxillopoda	Cyclopoida	Cyclopidae
EFX88758	Glp	Common water flea	*Daphnia pulex*	Branchiopoda	Diplostraca	Daphniidae
EFX88760	Glp	Common water flea	*Daphnia pulex*	Branchiopoda	Diplostraca	Daphniidae
EFX88757	Glp	Common water flea	*Daphnia pulex*	Branchiopoda	Diplostraca	Daphniidae
EFX66203	Glp	Common water flea	*Daphnia pulex*	Branchiopoda	Diplostraca	Daphniidae
EFX79826	Glp	Common water flea	*Daphnia pulex*	Branchiopoda	Diplostraca	Daphniidae
EFX88619	Glp	Common water flea	*Daphnia pulex*	Branchiopoda	Diplostraca	Daphniidae
BJ931504	Glp	Water flea	*Daphnia magna*	Branchiopoda	Diplostraca	Daphniidae
BJ935218/BJ935219	Glp	Water flea	*Daphnia magna*	Branchiopoda	Diplostraca	Daphniidae
GR508656	Glp	Water flea	*Daphnia carinata*	Branchiopoda	Diplostraca	Daphniidae
JW965215	Glp	Argulus	*Argulus siamensis*	Maxillopoda	Arguloida	Argulidae
JW969733	Glp	Argulus	*Argulus siamensis*	Maxillopoda	Arguloida	Argulidae
JW965635	Glp	Argulus	*Argulus siamensis*	Maxillopoda	Arguloida	Argulidae
JT035837	Glp A1	African social eresid spider	*Stegodyphus mimosarum*	Arachnida	Araneae	Eresidae
JT043093	Glp A1	African social eresid spider	*Stegodyphus tentoriicola*	Arachnida	Araneae	Eresidae
JT030770	Glp A1	African social eresid spider	*Stegodyphus lineatus*	Arachnida	Araneae	Eresidae
GBCS01013118	Glp A1	Western black widow	*Latrodectus hesperus*	Arachnida	Araneae	Theridiidae
AOMJ01012457/AOMJ01012461/AOMJ01099890	Glp A1	Common house spider	*Parasteatoda tepidariorum*	Arachnida	Araneae	Theridiidae
AYEL01063830/AYEL01063829/AYEL01055244	Glp A1	Chinese scorpion	*Mesobuthus martensii*	Arachnida	Scorpiones	Buthidae
AXZI01008059/AXZI01161692	Glp A1	Baja California bark scorpion	*Centruroides exilicauda*	Arachnida	Scorpiones	Buthidae
JT039102	Glp A2	African social eresid spider	*Stegodyphus mimosarum*	Arachnida	Araneae	Eresidae
JT042431	Glp A2	African social eresid spider	*Stegodyphus tentoriicola*	Arachnida	Araneae	Eresidae
JT030628	Glp A2	African social eresid spider	*Stegodyphus lineatus*	Arachnida	Araneae	Eresidae
GBCS01013464	Glp A2	Western black widow	*Latrodectus hesperus*	Arachnida	Araneae	Theridiidae
AOMJ01263225/AOMJ01263218	Glp A2	Common house spider	*Parasteatoda tepidariorum*	Arachnida	Araneae	Theridiidae
AYEL01089575/AYEL01071210	Glp A2	Chinese scorpion	*Mesobuthus martensii*	Arachnida	Scorpiones	Buthidae
JK732090	Glp A2	Brazilian scorpion	*Tityus serrulatus*	Arachnida	Scorpiones	Buthidae
AXZI01007911	Glp A2	Baja California bark scorpion	*Centruroides exilicauda*	Arachnida	Scorpiones	Buthidae
DR448116/DR447154	Glp A2L	São paulo black tarantula	*Acanthoscurria gomesiana*	Arachnida	Araneae	Theraphosidae
GAZS01013081	Glp A2L	Brazilian giant whiteknee tarantula	*Acanthoscurria geniculata*	Arachnida	Araneae	Theraphosidae
AEO32269	Glp A	Gulf coast tick	*Amblyomma maculatum*	Acari	Ixodida	Ixodidae
JAC33429	Glp A	Rickettsia tick	*Amblyomma triste*	Acari	Ixodida	Ixodidae
JAC25831	Glp A	Rickettsia tick	*Amblyomma parvum*	Acari	Ixodida	Ixodidae
JAC21331	Glp A	Cayenne tick	*Amblyomma cajennense*	Acari	Ixodida	Ixodidae
GACK01004617	Glp A	Zebra tick	*Rhipicephalus pulchellus*	Acari	Ixodida	Ixodidae
CD794748/CD794747	Glp A	Brown ear tick	*Rhipicephalus appendiculatus*	Acari	Ixodida	Ixodidae
CAX48963	Glp A	Brown dog tick	*Rhipicephalus sanguineus*	Acari	Ixodida	Ixodidae
JAA73021	Glp A	Castor bean tick	*Ixodes ricinus*	Acari	Ixodida	Ixodidae
ABJB010692148/ABJB010720745/ABJB010408716/ ABJB010406759	Glp A	Blacklegged tick	*Ixodes scapularis*	Acari	Ixodida	Ixodidae
XP_003745281	Glp A	Western predatory mite	*Metaseiulus occidentalis*	Acari	Mesostigmata	Phytoseiidae

JL016378	Glp A	Western predatory mite	*Metaseiulus occidentalis*	Acari	Mesostigmata	Phytoseiidae
CAEY01000889	Glp A	Two-spotted spider mite	*Tetranychus urticae*	Acari	Eleutherengona	Tetranychidae
tetur04g02680	Glp A	Two-spotted spider mite	*Tetranychus urticae*	Acari	Eleutherengona	Tetranychidae
tetur01g15060	Glp A	Two-spotted spider mite	*Tetranychus urticae*	Acari	Eleutherengona	Tetranychidae
AEO36644	Glp B1	Gulf coast tick	*Amblyomma maculatum*	Acari	Ixodida	Ixodidae
JAC35177	Glp B1	Rickettsia tick	*Amblyomma triste*	Acari	Ixodida	Ixodidae
JAC25752	Glp B1	Rickettsia tick	*Amblyomma parvum*	Acari	Ixodida	Ixodidae
JAC22662	Glp B1	Cayenne tick	*Amblyomma cajennense*	Acari	Ixodida	Ixodidae
GACK01008102	Glp B1	Zebra tick	*Rhipicephalus pulchellus*	Acari	Ixodida	Ixodidae
CAR66115	Glp B1	Brown dog tick	*Rhipicephalus sanguineus*	Acari	Ixodida	Ixodidae
CD780384	Glp B1	Brown ear tick	*Rhipicephalus appendiculatus*	Acari	Ixodida	Ixodidae
ABJB010086453/ABJB010131197/ABJB010002103	Glp B2a	Blacklegged tick	*Ixodes scapularis*	Acari	Ixodida	Ixodidae
EEC04800	Glp B2b	Blacklegged tick	*Ixodes scapularis*	Acari	Ixodida	Ixodidae
CAX48964	Glp B2a	Castor bean tick	*Ixodes ricinus*	Acari	Ixodida	Ixodidae
JAA71533	Glp B2b	Castor bean tick	*Ixodes ricinus*	Acari	Ixodida	Ixodidae
JAC32051	Glp B2	Rickettsia tick	*Amblyomma triste*	Acari	Ixodida	Ixodidae
JAC25679	Glp B2	Rickettsia tick	*Amblyomma parvum*	Acari	Ixodida	Ixodidae
JAC19979	Glp B2	Cayenne tick	*Amblyomma cajennense*	Acari	Ixodida	Ixodidae
JAC25782	Glp B3	Rickettsia tick	*Amblyomma parvum*	Acari	Ixodida	Ixodidae
JAC21377	Glp B3	Cayenne tick	*Amblyomma cajennense*	Acari	Ixodida	Ixodidae
GAGD01000711	Glp B3	Lone Star tick	*Amblyomma americanum*	Acari	Ixodida	Ixodidae
JAA56054	Glp B3	Zebra tick	*Rhipicephalus pulchellus*	Acari	Ixodida	Ixodidae
GADI01002277	Glp B3	Castor bean tick	*Ixodes ricinus*	Acari	Ixodida	Ixodidae
EEC20079	Glp B3	Blacklegged tick	*Ixodes scapularis*	Acari	Ixodida	Ixodidae
ABI53034	Glp B4	American dog tick	*Dermacentor variabilis*	Acari	Ixodida	Ixodidae
CK190314/CK190314	Glp B4	Southern cattle tick	*Rhipicephalus microplus*	Acari	Ixodida	Ixodidae
ABJB011072005/ABJB010219880	Glp B4	Blacklegged tick	*Ixodes scapularis*	Acari	Ixodida	Ixodidae
GANL01004539	Glp B	Black widow	*Latrodectus tredecimguttatus*	Arachnida	Araneae	Theridiidae
GBCS01000276	Glp B	Western black widow	*Latrodectus hesperus*	Arachnida	Araneae	Theridiidae
AOMJ01123248/AOMJ01123250/AOMJ01123251	Glp B	Common house spider	*Parasteatoda tepidariorum*	Arachnida	Araneae	Theridiidae
AOMJ01110087/AOMJ01110082/AOMJ01110081/ AOMJ01110079	Glp B	Common house spider	*Parasteatoda tepidariorum*	Arachnida	Araneae	Theridiidae
GAZS01045747	Glp B	Brazilian giant whiteknee tarantula	*Acanthoscurria geniculata*	Arachnida	Araneae	Theraphosidae
tetur01g07330	Glp B	Two-spotted spider mite	*Tetranychus urticae*	Acari	Eleutherengona	Tetranychidae
CAEY01001013/CAEY01001014	Glp B	Two-spotted spider mite	*Tetranychus urticae*	Acari	Eleutherengona	Tetranychidae
CAEY01001579	Glp B	Two-spotted spider mite	*Tetranychus urticae*	Acari	Eleutherengona	Tetranychidae
JT037994	Glp B	African social eresid spider	*Stegodyphus mimosarum*	Arachnida	Araneae	Eresidae
JT030404	Glp B	African social eresid spider	*Stegodyphus lineatus*	Arachnida	Araneae	Eresidae
GBCS01013198	Glp B	Western black widow	*Latrodectus hesperus*	Arachnida	Araneae	Theridiidae
AYEL01071210	Glp B	Chinese scorpion	*Mesobuthus martensii*	Arachnida	Scorpiones	Buthidae
AXZI01007912/AXZI01007914/AXZI01007916	Glp B	Baja California bark scorpion	*Centruroides exilicauda*	Arachnida	Scorpiones	Buthidae
**Other Protosotomia aquaglyceroporins**						
AEP14563	Glp 9	Water bear	*Milnesium tardigradum*	Tardigrada	Apochela	Milnesiidae
AEP14555	Glp (1)	Water bear	*Milnesium tardigradum*	Tardigrada	Apochela	Milnesiidae
AEP14558	Glp (4)	Water bear	*Milnesium tardigradum*	Tardigrada	Apochela	Milnesiidae
AEP14562	Glp (8)	Water bear	*Milnesium tardigradum*	Tardigrada	Apochela	Milnesiidae
AEP14556	Glp (2)	Water bear	*Milnesium tardigradum*	Tardigrada	Apochela	Milnesiidae
AEP14557	Glp (3)	Water bear	*Milnesium tardigradum*	Tardigrada	Apochela	Milnesiidae
AEP14564	Glp (10)	Water bear	*Milnesium tardigradum*	Tardigrada	Apochela	Milnesiidae
AEP14561	Glp (7)	Water bear	*Milnesium tardigradum*	Tardigrada	Apochela	Milnesiidae
FM924751	Glp	Rotifer	*Brachionus plicatilis*	Rotifera	Ploimida	Brachionidae
GACQ01008231	Glp	Rotifer	*Brachionus calyciflorus*	Rotifera	Ploimida	Brachionidae
GACL01028067	Glp	Rotifer	*Brachionus calyciflorus*	Rotifera	Ploimida	Brachionidae
GACQ01018937	Glp	Rotifer	*Brachionus calyciflorus*	Rotifera	Ploimida	Brachionidae
EKC31383	Glp	Pacific oyster	*Crassostrea gigas*	Mollusca	Ostreoida	Ostreidae
LotgiP157872	Glp	Owl limpet	*Lottia gigantea*	Mollusca		Lottiidae
LotgiP128629	Glp	Owl limpet	*Lottia gigantea*	Mollusca		Lottiidae
LotgiP74879	Glp	Owl limpet	*Lottia gigantea*	Mollusca		Lottiidae
LotgiP128639	Glp	Owl limpet	*Lottia gigantea*	Mollusca		Lottiidae
GAEN01011661	Glp	Mediterranean mussel	*Mytilus galloprovincialis*	Mollusca	Mytiloida	Mytilidae
JR510544	Glp	Freshwater mussel	*Villosa lienosa*	Mollusca	Unionoida	Unionidae
GAGS01074101	Glp	Freshwater snail	*Bithynia siamensis goniomphalos*	Mollusca	Littorinimorpha	Bithyniidae
GAGS01100777	Glp	Freshwater snail	*Bithynia siamensis goniomphalos*	Mollusca	Littorinimorpha	Bithyniidae
XP_005107446	Glp	California sea hare	*Aplysia californica*	Mollusca		Aplysiidae
Smp_005720	Glp	Flatworm	*Schistosoma mansoni*	Platyhelminthes	Strigeidida	Schistosomatidae
Smp_005740	Glp	Flatworm	*Schistosoma mansoni*	Platyhelminthes	Strigeidida	Schistosomatidae
Smp_125200	Glp	Flatworm	*Schistosoma mansoni*	Platyhelminthes	Strigeidida	Schistosomatidae
Smp_125210	Glp	Flatworm	*Schistosoma mansoni*	Platyhelminthes	Strigeidida	Schistosomatidae
Smp_125220	Glp	Flatworm	*Schistosoma mansoni*	Platyhelminthes	Strigeidida	Schistosomatidae
CABF01070090/CABF01070089	Glp	Flatworm	*Schistosoma japonicum*	Platyhelminthes	Strigeidida	Schistosomatidae
CABF01033520	Glp	Flatworm	*Schistosoma japonicum*	Platyhelminthes	Strigeidida	Schistosomatidae
JR935701	Glp	Flatworm	*Taenia multiceps*	Platyhelminthes	Cyclophyllidea	Taeniidae
JR925719	Glp	Flatworm	*Taenia multiceps*	Platyhelminthes	Cyclophyllidea	Taeniidae
CapteP125390	Glp	Segmented worm	*Capitella tellata*	Annelida	Capitellida	Capitellidae
CapteP164915	Glp	Segmented worm	*Capitella tellata*	Annelida	Capitellida	Capitellidae
HelroP95422	Glp	Freshwater leech	*Helobdella robusta*	Annelida	Rhynchobdellida	Glossiphoniidae
AAC32826	Glp	Roundworm	*Toxocara canis*	Nematoda	Ascaridida	Toxocaridae
K02G10.7	Glp	Roundworm	*Caenorhabditis elegans*	Nematoda	Rhabditida	Rhabditidae
F32A5.5	Glp	Roundworm	*Caenorhabditis elegans*	Nematoda	Rhabditida	Rhabditidae
C01G6.1a	Glp	Roundworm	*Caenorhabditis elegans*	Nematoda	Rhabditida	Rhabditidae
M02F4.8	Glp	Roundworm	*Caenorhabditis elegans*	Nematoda	Rhabditida	Rhabditidae
Y69E1A.7	Glp	Roundworm	*Caenorhabditis elegans*	Nematoda	Rhabditida	Rhabditidae
CBN25211	Glp	Roundworm	*Caenorhabditis brenneri*	Nematoda	Rhabditida	Rhabditidae
CBN22859	Glp	Roundworm	*Caenorhabditis brenneri*	Nematoda	Rhabditida	Rhabditidae
CBN16306	Glp	Roundworm	*Caenorhabditis brenneri*	Nematoda	Rhabditida	Rhabditidae
CBN20801	Glp	Roundworm	*Caenorhabditis brenneri*	Nematoda	Rhabditida	Rhabditidae
CBN20105	Glp	Roundworm	*Caenorhabditis brenneri*	Nematoda	Rhabditida	Rhabditidae
CBN04043	Glp	Roundworm	*Caenorhabditis brenneri*	Nematoda	Rhabditida	Rhabditidae
CBG13111	Glp	Roundworm	*Caenorhabditis briggsae*	Nematoda	Rhabditida	Rhabditidae
CBG00920	Glp	Roundworm	*Caenorhabditis briggsae*	Nematoda	Rhabditida	Rhabditidae
CBG05966	Glp	Roundworm	*Caenorhabditis briggsae*	Nematoda	Rhabditida	Rhabditidae
CBG14117	Glp	Roundworm	*Caenorhabditis briggsae*	Nematoda	Rhabditida	Rhabditidae
CBG14525	Glp	Roundworm	*Caenorhabditis briggsae*	Nematoda	Rhabditida	Rhabditidae
PPA25203	Glp	Roundworm	*Pristionchus pacificus*	Nematoda	Diplogasterida	Neodiplogasteridae
PPA25204	Glp	Roundworm	*Pristionchus pacificus*	Nematoda	Diplogasterida	Neodiplogasteridae
PPA00131	Glp	Roundworm	*Pristionchus pacificus*	Nematoda	Diplogasterida	Neodiplogasteridae
PPA23301	Glp	Roundworm	*Pristionchus pacificus*	Nematoda	Diplogasterida	Neodiplogasteridae
PPA28936	Glp	Roundworm	*Pristionchus pacificus*	Nematoda	Diplogasterida	Neodiplogasteridae
PPA03108	Glp	Roundworm	*Pristionchus pacificus*	Nematoda	Diplogasterida	Neodiplogasteridae

PPA03114	Glp	Roundworm	*Pristionchus pacificus*	Nematoda	Diplogasterida	Neodiplogasteridae
PPA28655	Glp	Roundworm	*Pristionchus pacificus*	Nematoda	Diplogasterida	Neodiplogasteridae
XP_001892113	Glp	Roundworm	*Brugia malayi*	Nematoda	Spirurida	Onchocercidae
XP_003137207	Glp	Eye worm	*Loa loa*	Nematoda	Spirurida	Onchocercidae
XP_003147351	Glp	Eye worm	*Loa loa*	Nematoda	Spirurida	Onchocercidae
**Parazoa/Radiata aquaglyceroporins**						
BACK01014751/BACK01014752	Glp 1	Stony coral	*Acropora digitifera*	Cnidaria	Scleractinia	Acroporidae
JR999939	Glp 1	Stony coral	*Acropora millepora*	Cnidaria	Scleractinia	Acroporidae
DC999942	Glp 1	Stony coral	*Acropora tenuis*	Cnidaria	Scleractinia	Acroporidae
GASU01015432	Glp 1	Stony coral	*Acropora cervicornis*	Cnidaria	Scleractinia	Acroporidae
GW195723	Glp 1	Stony coral	*Acropora palmata*	Cnidaria	Scleractinia	Acroporidae
GARY01000511	Glp 1	Stony coral	*Stylophora pistillata*	Cnidaria	Scleractinia	Pocilloporidae
BACK01014753/BACK01014752	Glp 1b	Stony coral	*Acropora digitifera*	Cnidaria	Scleractinia	Acroporidae
JR980480	Glp 1b	Stony coral	*Acropora millepora*	Cnidaria	Scleractinia	Acroporidae
GASU01059074	Glp 1b	Stony coral	*Acropora cervicornis*	Cnidaria	Scleractinia	Acroporidae
FX438739	Glp 1b	Stony coral	*Porites australiensis*	Cnidaria	Scleractinia	Poritidae
GE909075	Glp 1b	Stony coral	*Porites astreoides*	Cnidaria	Scleractinia	Poritidae
GARY01000511	Glp 1b	Stony coral	*Stylophora pistillata*	Cnidaria	Scleractinia	Pocilloporidae
BACK01014751	Glp 2	Stony coral	*Acropora digitifera*	Cnidaria	Scleractinia	Acroporidae
JR978591	Glp 2	Stony coral	*Acropora millepora*	Cnidaria	Scleractinia	Acroporidae
GASU01092011	Glp 2	Stony coral	*Acropora cervicornis*	Cnidaria	Scleractinia	Acroporidae
GW270188	Glp 2	Stony coral	*Montastraea faveolata*	Cnidaria	Scleractinia	Montastraeidae
FX459378	Glp 2	Stony coral	*Porites australiensis*	Cnidaria	Scleractinia	Poritidae
GARY01025768	Glp 2	Stony coral	*Stylophora pistillata*	Cnidaria	Scleractinia	Pocilloporidae
v1g162785/XM_001637091	Glp 2a	Starlet sea anemone	*Nematostella vectensis*	Cnidaria	Actiniaria	Edwardsiidae
v1g93479/ XP_001637090	Glp 2b	Starlet sea anemone	*Nematostella vectensis*	Cnidaria	Actiniaria	Edwardsiidae
v1g4182/XP_001620982	Glp 2c	Starlet sea anemone	*Nematostella vectensis*	Cnidaria	Actiniaria	Edwardsiidae
v1g243020/XP_001633058	Glp 2d	Starlet sea anemone	*Nematostella vectensis*	Cnidaria	Actiniaria	Edwardsiidae
JV099621	Glp 2a	Symbiotic anemone	*Aiptasia pallida*	Cnidaria	Actiniaria	Aiptasiidae
JV119766	Glp 2b	Symbiotic anemone	*Aiptasia pallida*	Cnidaria	Actiniaria	Aiptasiidae
JV113575	Glp 2c	Symbiotic anemone	*Aiptasia pallida*	Cnidaria	Actiniaria	Aiptasiidae
XP_002162049	Glp 1a	Freshwater anemone	*Hydra vulgaris*	Cnidaria	Hydroida	Hydridae
XP_002165391	Glp 1b	Freshwater anemone	*Hydra vulgaris*	Cnidaria	Hydroida	Hydridae
XP_002165440	Glp 1c	Freshwater anemone	*Hydra vulgaris*	Cnidaria	Hydroida	Hydridae
FP974057	Glp 1	Thecate hydroid	*Clytia hemisphaerica*	Cnidaria	Hydroida	Campanulariidae
XP_002156497	Glp 2	Freshwater anemone	*Hydra vulgaris*	Cnidaria	Hydroida	Hydridae
CU427936	Glp 2	Thecate hydroid	*Clytia hemisphaerica*	Cnidaria	Hydroida	Campanulariidae
XP_002108654	Glp	Placozoan	*Trichoplax adhaerens*	Placozoa		
Aqu1_227820/XP_003383528/PAC_15726348/ ACUQ01000663	Glp 1	Sea sponge	*Amphimedon queenslandica*	Porifera	Haplosclerida	Niphatidae
AM760644	Glp 1	Mueller's freshwater sponge	*Ephydatia muelleri*	Porifera	Haplosclerida	Spongillidae
CAK22281	Glp 1	Lake Baikal sponge	*Lubomirskia baicalensis*	Porifera	Haplosclerida	Lubomirskiidae
GO086050	Glp 1a	Sponge	*Heterochone calyx*	Porifera	Hexactinosida	Aphrocallistidae
GO087389	Glp 1b	Sponge	*Heterochone calyx*	Porifera	Hexactinosida	Aphrocallistidae
Aqu1_227913/PAC_15726441/XP_003383384/ ACUQ01000619	Glp 2a	Sea sponge	*Amphimedon queenslandica*	Porifera	Haplosclerida	Niphatidae
PAC_1571972/Aqu1_221200/8/XP_003386535/ ACUQ01003032	Glp 2b	Sea sponge	*Amphimedon queenslandica*	Porifera	Haplosclerida	Niphatidae
PAC_15719729/ACUQ01003032	Glp 2c	Sea sponge	*Amphimedon queenslandica*	Porifera	Haplosclerida	Niphatidae
ETX05898	Glp F	Sea sponge symbiont	*Candidatus entotheonella sp.*	Proteobacteria		
ETW98335	Glp F	Sea sponge symbiont	*Candidatus entotheonella sp.*	Proteobacteria		
**Protista, Fungi aquaglyceroporins**						
DDB0167571	Glp 1	Cellular slime mold	*Dictyostelium discoideum*	Amoebozoa	Dictyosteliida	
AJWI01003455	Glp 1	Cellular slime mold	*Dictyostelium intermedium*	Amoebozoa	Dictyosteliida	
XP_004350034	Glp 1	Cellular slime mold	*Dictyostelium fasciculatum*	Amoebozoa	Dictyosteliida	
DDB0189011	Glp 2	Cellular slime mold	*Dictyostelium discoideum*	Amoebozoa	Dictyosteliida	
AJWI01002093	Glp 2	Cellular slime mold	*Dictyostelium intermedium*	Amoebozoa	Dictyosteliida	
XP_003294465	Glp 2	Cellular slime mold	*Dictyostelium purpureum*	Amoebozoa	Dictyosteliida	
XP_001615442/PVX_092245	Glp	Plasmodium parasite	*Plasmodium vivax*	Apicomplexa	Haemosporida	
PKH_093060	Glp	Plasmodium parasite	*Plasmodium knowlesi*	Apicomplexa	Haemosporida	
PF11_0338-1	Glp	Plasmodium parasite	*Plasmodium falciparum*	Apicomplexa	Haemosporida	
EAN78784	Glp 1	Trypanosome	*Trypanosoma brucei brucei*	Euglenozoa	Kinetoplastida	Trypanosomatidae
EAN78785	Glp 2	Trypanosome	*Trypanosoma brucei brucei*	Euglenozoa	Kinetoplastida	Trypanosomatidae
AAZ11715	Glp 3	Trypanosome	*Trypanosoma brucei brucei*	Euglenozoa	Kinetoplastida	Trypanosomatidae
Phatr44871	Glp	Diatom	*Phaeodactylum tricornutum*	Bacillariophyta	Naviculales	Phaeodactylaceae
Phatrdraft1692	Glp	Diatom	*Phaeodactylum tricornutum*	Bacillariophyta	Naviculales	Phaeodactylaceae
Thaps924	Glp	Diatom	*Thalassiosira pseudonana*	Bacillariophyta	Thalassiosirales	Thalassiosiraceae
CCA23301	Glp 1	Water mold	*Albugo laibachii*	Oomycetes	Albuginales	Albuginaceae
CCA21136	Glp 2	Water mold	*Albugo laibachii*	Oomycetes	Albuginales	Albuginaceae
PYU1_T004484	Glp	Water mold	*Pythium ultimum*	Oomycetes	Pythiales	Pythiaceae
EDP47128	Glp 1	Fungus	*Aspergillus fumigatus*	Ascomycota	Eurotiales	Aspergillaceae
XP_001267577	Glp 1	Fungus	*Neosartorya fischeri*	Ascomycota	Eurotiales	Aspergillaceae
XP_001270514	Glp 1	Fungus	*Aspergillus clavatus*	Ascomycota	Eurotiales	Aspergillaceae
CAP99447	Glp 1	Fungus	*Penicillium chrysogenum Wisconsin*	Ascomycota	Eurotiales	Aspergillaceae
EED51102	Glp 1	Fungus	*Aspergillus flavus*	Ascomycota	Eurotiales	Aspergillaceae
XP_001258974	Glp 2	Fungus	*Neosartorya fischeri*	Ascomycota	Eurotiales	Aspergillaceae
XP_001273629	Glp 2	Fungus	*Aspergillus clavatus*	Ascomycota	Eurotiales	Aspergillaceae
EED24013	Glp 2	Fungus	*Talaromyces stipitatus*	Ascomycota	Eurotiales	Trichocomaceae
XP_001941733	Glp 2	Fungus	*Pyrenophora tritici-repentis*	Ascomycota	Pleosporales	Pleosporaceae
YFL054C	Glp	Baker´s yeast	*Saccharomyces cerevisiae*	Ascomycota	Saccharomycetales	Saccharomycetaceae
NP_592788	Glp	Fission yeast	*Schizosaccharomyces pombe*	Ascomycota	Schizosaccharomycet	Schizosaccharomycetace
XP_750737	Glp 3	Fungus	*Aspergillus fumigatus*	Ascomycota	Eurotiales	Aspergillaceae
XP_001257964	Glp 3	Fungus	*Neosartorya fischeri*	Ascomycota	Eurotiales	Aspergillaceae
XP_001267954	Glp 3	Fungus	*Aspergillus clavatus*	Ascomycota	Eurotiales	Aspergillaceae
CAP98622	Glp 3	Fungus	*Penicillium chrysogenum Wisconsin*	Ascomycota	Eurotiales	Aspergillaceae
EED48588	Glp 3	Fungus	*Aspergillus flavus*	Ascomycota	Eurotiales	Aspergillaceae
EED22000	Glp 3	Fungus	*Talaromyces stipitatus*	Ascomycota	Eurotiales	Trichocomaceae
XP_503595	Glp 3a	Fungus	*Yarrowia lipolytica*	Ascomycota	Saccharomycetales	Dipodascaceae
XP_504820	Glp 3b	Fungus	*Yarrowia lipolytica*	Ascomycota	Saccharomycetales	Dipodascaceae
XP_001878805	Glp 3a	Fungus	*Laccaria bicolor*	Ascomycota	Agaricales	Tricholomataceae
XP_001878806	Glp 3b	Fungus	*Laccaria bicolor*	Ascomycota	Agaricales	Tricholomataceae
**Hexapod fungal (endosymbiont) glycerol facilitators**						
GBIT01045282	Glp	Oriental citrus fly	*Bactrocera minax*	Hexapoda	Diptera	Tephritidae
GADH01000376	Glp	Dobsonfly	*Corydalinae sp.*	Hexapoda	Megaloptera	Corydalidae
GBDP01047288	Glp	Common lac scale	*Kerria lacca*	Hexapoda	Hemiptera	Kerriidae
GATB01334705	Glp	Metallyticid mantis	*Metallyticus splendidus*	Hexapoda	Mantodea	Metallyticidae
**Hexapod bacteroid (endosymbiont) glycerol facilitators**						

XM_004532995	Glp F	Mediterranean fruit fly	*Ceratitis capitata*	Hexapoda	Diptera	Tephritidae
JHUJ01073854	Glp F	Australian sheep blowfly	*Lucilia cuprina*	Hexapoda	Diptera	Calliphoridae
JDSM01348676	Glp F	Caddisfly	*Limnephilus lunatus*	Hexapoda	Trichoptera	Limnephilidae
ACPB02009318	Glp F	Assassin bug	*Rhodnius prolixus*	Hexapoda	Hemiptera	Reduviidae
APGL01000222	Glp F	Mountain pine beetle	*Dendroctonus ponderosae*	Hexapoda	Coleoptera	Curculionidae
JMDY01040223	Glp F	Western flower thrips	*Frankliniella occidentalis*	Hexapoda	Thysanoptera	Thripidae
WP_012840982	Glp F	German cockroach	*Blattella germanica*	Hexapoda	Blattodea	Ectobiidae
JPZV01291450	Glp F	German cockroach	*Blattella germanica*	Hexapoda	Blattodea	Ectobiidae
GAWS01261431	Glp F	American cockroach	*Periplaneta americana*	Hexapoda	Blattodea	Blattidae
WP_014726498	Glp F	Blaberus cockroach	*Blaberus atropos*	Hexapoda	Blattodea	Blaberidae
**Prokaryotic glycerol facilitators**						
NP_290556/EBESCP00000004421	Glp F	Enterobacter	*Escherichia coli*	Proteobacteria	Enterobacteriales	Enterobacteriaceae
YP_405253/EBESCP00000079777	Glp F	Enterobacter	*Shigella dysenteriae*	Proteobacteria	Enterobacteriales	Enterobacteriaceae
YP_006479893	Glp F	Enterobacter	*Enterobacter cloacae subsp*	Proteobacteria	Enterobacteriales	Enterobacteriaceae
WP_008109681	Glp F	Pantoea	*Pantoea sp.*	Proteobacteria	Enterobacteriales	Enterobacteriaceae
WP_009637726	Glp F	Serratia	*Serratia sp.*	Proteobacteria	Enterobacteriales	Enterobacteriaceae
WP_010299311	Glp F	Pectobacterium	*Pectobacterium carotovorum*	Proteobacteria	Enterobacteriales	Enterobacteriaceae
**Unorthodox aquaporins**						
**Deuterostomia unorthodox aquaporins**						
ENSP00000318770	AQP 11	Human	*Homo sapiens*	Euarchontoglires	Primates	Hominidae
ENSPTRP00000007062	AQP 11	Chimpanzee	*Pan troglodytes*	Euarchontoglires	Primates	Hominidae
ENSGGOP00000011686	AQP 11	Western lowland gorilla	*Gorilla gorilla gorilla*	Euarchontoglires	Primates	Hominidae
ENSPPYP00000004239	AQP 11	Sumatran orangutan	*Pongo abelii*	Euarchontoglires	Primates	Hominidae
ENSNLEP00000020273	AQP 11	Northern white-cheeked gibbon	*Nomascus leucogenys*	Euarchontoglires	Primates	Hylobatidae
ENSMMUP00000024926	AQP 11	Rhesus macaque	*Macaca mulatta*	Euarchontoglires	Primates	Cercopithecidae
ENSP00000318770	AQP 11	Hamadryas baboon	*Papio hamadryas*	Euarchontoglires	Primates	Cercopithecidae
XM_003910458	AQP 11	Olive baboon	*Papio anubis*	Euarchontoglires	Primates	Cercopithecidae
ENSCJAP00000027512	AQP 11	White-tufted-ear marmoset	*Callithrix jacchus*	Euarchontoglires	Primates	Cebidae
XP_003935086	AQP 11	Bolivian squirrel monkey	*Saimiri boliviensis boliviensis*	Euarchontoglires	Primates	Cebidae
ABRT010057814	AQP 11	Philippine tarsier	*Tarsius syrichta*	Euarchontoglires	Primates	Tarsiidae
ABDC01304253	AQP 11	Gray mouse lemur	*Microcebus murinus*	Euarchontoglires	Primates	Cheirogaleidae
ENSOGAP00000008223	AQP 11	Small-eared galago/Bushbaby	*Otolemur garnettii*	Euarchontoglires	Primates	Galagidae
AAPY01308260	AQP 11	Northern tree shrew	*Tupaia belangeri*	Euarchontoglires	Scandentia	Tupaiidae
ALAR01061175	AQP 11	Chinese tree shrew	*Tupaia chinensis*	Euarchontoglires	Scandentia	Tupaiidae
ENSMUSP00000082054	AQP 11	Mouse	*Mus musculus*	Euarchontoglires	Rodentia	Muridae
ENSRNOP00000018091	AQP 11	Norway rat	*Rattus norvegicus*	Euarchontoglires	Rodentia	Muridae
XP_003510445	AQP 11	Chinese hamster	*Cricetulus griseus*	Euarchontoglires	Rodentia	Cricetidae
ENSDORP00000015180	AQP 11	Ord's kangaroo rat	*Dipodomys ordii*	Euarchontoglires	Rodentia	Heteromyidae
ENSCPOP00000004727	AQP 11	Domestic guinea pig	*Cavia porcellus*	Euarchontoglires	Rodentia	Caviidae
EHB01284	AQP 11	Naked mole-rat	*Heterocephalus glaber*	Euarchontoglires	Rodentia	Bathyergidae
ENSSTOP00000003585	AQP 11	Thirteen-lined ground squirrel	*Ictidomys tridecemlineatus*	Euarchontoglires	Rodentia	Sciuridae
ENSOPRP00000009607	AQP 11	American pika	*Ochotona princeps*	Laurasiatheria	Lagomorpha	Ochotonidae
ENSOCUP00000002810	AQP 11	Rabbit	*Oryctolagus cuniculus*	Laurasiatheria	Lagomorpha	Leporidae
ENSSARP00000008545	AQP 11	Common shrew	*Sorex araneus*	Laurasiatheria	Insectivora	Soricidae
XP_004683290	AQP 11	Star-nosed mole	*Condylura cristata*	Laurasiatheria	Insectivora	Talpidae
ENSPVAP00000012492	AQP 11	Large flying fox/Megabat	*Pteropus vampyrus*	Laurasiatheria	Chiroptera	Pteropodidae
ENSMLUP00000008870	AQP 11	Little brown bat/Microbat	*Myotis lucifugus*	Laurasiatheria	Chiroptera	Vespertilionidae
ENSVPAP00000009668/XP_006213705	AQP 11	Alpaca	*Vicugna pacos*	Laurasiatheria	Tylopoda	Camelidae
XP_006191706	AQP 11	Wild Bactrian camel	*Camelus ferus*	Laurasiatheria	Tylopoda	Camelidae
XP_004019484/AMGL01060868	AQP 11	Sheep	*Ovis aries*	Laurasiatheria	Ruminantia	Bovidae
AJPT01229372	AQP 11	Goat	*Capra hircus*	Laurasiatheria	Ruminantia	Bovidae
ENSBTAP00000026547	AQP 11	Cow	*Bos taurus*	Laurasiatheria	Ruminantia	Bovidae
ELR47367	AQP 11	Yak	*Bos grunniens mutus*	Laurasiatheria	Ruminantia	Bovidae
AGFL01204710	AQP 11	Zebu	*Bos indicus*	Laurasiatheria	Ruminantia	Bovidae
ENSTTRP00000006655	AQP 11	Bottlenosed dolphin	*Tursiops truncatus*	Laurasiatheria	Cetacea	Delphinidae
ANOL02054196/XP_004279877	AQP 11	Killer whale	*Orcinus orca*	Laurasiatheria	Cetacea	Delphinidae
ENSSSCP00000015800	AQP 11	Pig	*Sus scrofa*	Laurasiatheria	Suina	Suidae
ENSAMEP00000005959	AQP 11	Giant panda	*Ailuropoda melanoleuca*	Laurasiatheria	Carnivora	Ursidae
ENSCAFP00000007308	AQP 11	Dog	*Canis lupus familiaris*	Laurasiatheria	Carnivora	Canidae
ENSCAFP00000007308	AQP 11	Domestic ferret	*Mustela putorius furo*	Laurasiatheria	Carnivora	Mustelidae
ENSFCAP00000006902/XP_003992750	AQP 11	Domestic cat	*Felis catus*	Laurasiatheria	Carnivora	Felidae
ENSECAP00000015158/XP_001494555	AQP 11	Horse	*Equus caballus*	Laurasiatheria	Perissodactyla	Equidae
XP_004434089	AQP 11	Southern white rhinocerus	*Ceratotherium simum simum*	Laurasiatheria	Perissodactyla	Rhinocerotidae
ENSLAFP00000023596	AQP 11	African savanna elephant	*Loxodonta africana*	Afrotheria	Proboscidea	Elephantidae
AHIN01084737/XP_004382050	AQP 11	Florida manatee	*Trichechus manatus latirostris*	Afrotheria	Sirenia	Trichechidae
ABRQ01192979	AQP 11	Cape rock hyrax	*Procavia capensis*	Afrotheria	Hyracoidea	Procaviidae
ENSETEP00000004273	AQP 11	Lesser hedgehog tenrec	*Echinops telfairi*	Afrotheria	Afrosoricida	Tenrecidae
ALYB01103069	AQP 11	Ardvaark	*Orycteropus afer afer*	Afrotheria	Tubulidentata	Orycteropodidae
ENSDNOP00000007102	AQP 11	Nine-banded armadillo	*Dasypus novemcinctus*	Xenarthra	Cingulata	Dasypodidae
ENSCHOP00000011681	AQP 11	Hoffmann's two-fingered sloth	*Choloepus hoffmanni*	Xenarthra	Pilosa	Megalonychidae
ENSMEUP00000004424	AQP 11	Tammar wallaby	*Macropus eugenii*	Metatheria	Diprotodontia	Macropodidae
ENSSHAP00000012436	AQP 11	Tasmanian devil	*Sarcophilus harrisii*	Metatheria	Dasyuromorphia	Dasyuridae
ENSMODP00000006192	AQP 11	Gray short-tailed opossum	*Monodelphis domestica*	Metatheria	Didelphimorphia	Didelphidae
ENSOANP00000015652	AQP 11	Platypus	*Ornithorhynchus anatinus*	Prototheria	Monotremata	Ornithorhynchidae
AGAI01051433	AQP 11	Budgerigar	*Melopsittacus undulatus*	Aves	Psittaciformes	Psittacidae
AMXX01102566/AMXX01186982/AOUJ01032445/ AOUJ01032441	AQP 11	Scarlet macaw	*Ara macao*	Aves	Psittaciformes	Psittacidae
CAVT010007100	AQP 11	Common canary	*Serinus canaria*	Aves	Passeriformes	Fringillidae
AKZB01048616	AQP 11	Medium ground finch	*Geospiza fortis*	Aves	Passeriformes	Fringillidae
XP_005495534	AQP 11	White-throated sparrow	*Zonotrichia albicollis*	Aves	Passeriformes	Fringillidae
ENSTGUP00000013419/ABQF01027693/ ABQF01027694	AQP 11	Zebra finch	*Taeniopygia guttata*	Aves	Passeriformes	Estrildidae
XP_005531056	AQP 11	Tibetan ground-tit	*Pseudopodoces humilis*	Aves	Passeriformes	Paridae
JR864707	AQP 11	Vinous-throated parrotbill	*Paradoxornis webbianus*	Aves	Passeriformes	Muscicapidae
AGTO01000778/XP_005038357	AQP 11	Collared flycatcher	*Ficedula albicollis*	Aves	Passeriformes	Muscicapidae
XP_005231201	AQP 11	Peregrin falcon	*Falco peregrinus*	Aves	Falconiformes	Falconidae
XP_005441017	AQP 11	Saker falcon	*Falco cherrug*	Aves	Falconiformes	Falconidae
XP_005500642	AQP 11	Rock pigeon	*Columba livia*	Aves	Columbiformes	Columbidae
ENSAPLP00000006367	AQP 11	Mallard	*Anas platyrhynchos*	Aves	Anseriformes	Anatidae
ENSMGAP00000016194	AQP 11	Turkey	*Meleagris gallopavo*	Aves	Galliformes	Phasianidae
ENSGALP00000002455	AQP 11	Chicken	*Gallus gallus*	Aves	Galliformes	Phasianidae
AKHW01075947	AQP 11	American alligator	*Alligator mississippiensis*	Archosauria	Crocodylia	Alligatoridae
AVPB01133327/AVPB01133328	AQP 11	Chinese alligator	*Alligator sinensis*	Archosauria	Crocodylia	Alligatoridae
ENSPSIP00000005055	AQP 11	Chinese softshell turtle	*Pelodiscus sinensis*	Sauropsida	Testudines	Trionychidae
EMP28605	AQP 11	Green seaturtle	*Chelonia mydas*	Sauropsida	Testudines	Cheloniidae
JW302207	AQP 11	Red-eared slider turtle	*Trachemys scripta elegans*	Sauropsida	Testudines	Emydidae
AHGY01077190	AQP 11	Western painted turtle	*Chrysemys picta bellii*	Sauropsida	Testudines	Emydidae
AZIM01006374	AQP 11	King cobra	*Ophiophagus hannah*	Lepidosauria	Squamata	Elapidae

AEQU02153142/AEQU02153141	AQP 11	Burmese python	*Python molurus bivittatus*	Lepidosauria	Squamata	Pythonidae
AAWZ02025389/ENSACAP00000001854	AQP 11	Green anole	*Anolis carolinensis*	Lepidosauria	Squamata	Iguanidae
AAI33223	AQP 11	African clawed frog	*Xenopus laevis*	Amphibia	Anura	Pipidae
ENSXETP00000058403	AQP 11	Western clawed frog	*Xenopus (Silurana) tropicalis*	Amphibia	Anura	Pipidae
FS311168	AQP 11	Japanese firebelly newt	*Cynops pyrrhogaster*	Amphibia	Caudata	Salamandridae
GAQK01082401	AQP 11	Chinese salamander	*Hynobius chinensis*	Amphibia	Caudata	Hynobiidae
AFYH01170435/AFYH01170433/ENSLACP00000007328	Aqp 11	Coelacanth	*Latimeria chalumnae*	Actinistia	Coelacanthiformes	Coelacanthidae
GAPS01010023	Aqp 11	Menado coelacanth	*Latimeria menadoensis*	Actinistia	Coelacanthiformes	Coelacanthidae
ENSTRUP00000025165	Aqp 11a	Torafugu	*Takifugu rubripes*	Acanthopterygii	Tetraodontiformes	Tetraodontidae
GSTENT10023417001	Aqp 11a	Green-spotted pufferfish	*Tetraodon nigroviridis*	Acanthopterygii	Tetraodontiformes	Tetraodontidae
AGRG01015803	Aqp 11a	Tongue sole	*Cynoglossus semilaevis*	Acanthopterygii	Pleuronectiformes	Cynoglossidae
AGTA02049030	Aqp 11a	Zebra mbuna	*Maylandia zebra*	Acanthopterygii	Perciformes	Cichlidae
AFNX01034372	Aqp 11a	Red mwanza	*Pundamilia nyererei*	Acanthopterygii	Perciformes	Cichlidae
AFNY01079048/AFNY01079049	Aqp 11a	Lyretail cichlid	*Neolamprologus brichardi*	Acanthopterygii	Perciformes	Cichlidae
AFNZ01043460	Aqp 11a	Burton's mouthbrooder	*Haplochromis burtoni*	Acanthopterygii	Perciformes	Cichlidae
ENSONIP00000012033/AERX01031972	Aqp 11a	Nile tilapia	*Oreochromis niloticus*	Acanthopterygii	Perciformes	Cichlidae
DB864935	Aqp 11a	Matumbi hunter	*Lipochromis sp.*	Acanthopterygii	Perciformes	Cichlidae
BADN01081994/BADN01081995	Aqp 11a	Pacific bluefin tuna	*Thunnus orientalis*	Acanthopterygii	Perciformes	Scombridae
GAJJ01007087/GAJJ01013977/AWGY01082912/AWGY01127580	Aqp 11a	Sablefish	*Anoplopoma fimbria*	Acanthopterygii	Scorpaeniformes	Anoplopomatidae
AUPQ01080838	Aqp 11a	Flag rockfish	*Sebastes rubrivinctus*	Acanthopterygii	Scorpaeniformes	Sebastidae
AUPR01165769	Aqp 11a	Tiger rockfish	*Sebastes nigrocinctus*	Acanthopterygii	Scorpaeniformes	Sebastidae
ENSGACP00000027582	Aqp 11a	Three-spined stickleback	*Gasterosteus aculeatus*	Acanthopterygii	Gasterosteiformes	Gasterosteidae
ENSORLP00000018866	Aqp 11a	Japanese medaka	*Oryzias latipes*	Acanthopterygii	Beloniformes	Adrianichthyidae
GAIB01110151	Aqp 11a	Turquoise killifish	*Nothobranchius furzeri*	Acanthopterygii	Cyprinodontiformes	Nothobranchiidae
HO912098	Aqp 11a	Blackspotted livebearer	*Poeciliopsis turneri*	Acanthopterygii	Cyprinodontiformes	Poeciliidae
AYCK01009593	Aqp 11a	Amazon molly	*Poecilia formosa*	Acanthopterygii	Cyprinodontiformes	Poeciliidae
ENSXMAP00000000645	Aqp 11a	Southern platyfish	*Xiphophorus maculatus*	Acanthopterygii	Cyprinodontiformes	Poeciliidae
ENSGMOP00000006569	Aqp 11a	Atlantic cod	*Gadus morhua*	Paracanthopterygii	Gadiformes	Gadidae
JP737092	Aqp 11a	Ayu	*Plecoglossus altivelis*	Protacanthopterygii	Osmeriformes	Osmeridae
EZ807701/EZ814810/CCAF010005608	Aqp 11a1	Rainbow trout	*Oncorhynchus mykiss*	Protacanthopterygii	Salmoniformes	Salmonidae
AGKD01092847/GE766931	Aqp 11a1	Atlantic salmon	*Salmo salar*	Protacanthopterygii	Salmoniformes	Salmonidae
CCAF010031865	Aqp 11a2	Rainbow trout	*Oncorhynchus mykiss*	Protacanthopterygii	Salmoniformes	Salmonidae
AGKD01029804	Aqp 11a2	Atlantic salmon	*Salmo salar*	Protacanthopterygii	Salmoniformes	Salmonidae
APWO01075838/ENSAMXP00000007062	Aqp 11a	Mexican tetra	*Astyanax mexicanus*	Ostariophysi	Characiformes	Characidae
AVPY01012376	Aqp 11a	Japanese eel	*Anguilla japonica*	Elopomorpha	Anguilliformes	Anguillidae
ENSTRUP00000017679	Aqp 11b	Torafugu	*Takifugu rubripes*	Acanthopterygii	Tetraodontiformes	Tetraodontidae
AOOT01001028/AOOT01001029/AOOT01001030	Aqp 11b	Sansaifugu	*Takifugu flavidus*	Acanthopterygii	Tetraodontiformes	Tetraodontidae
BAL44701	Aqp 11b	Mefugu	*Takifugu obscurus*	Acanthopterygii	Tetraodontiformes	Tetraodontidae
AGRG01014057	Aqp 11b	Tongue sole	*Cynoglossus semilaevis*	Acanthopterygii	Pleuronectiformes	Cynoglossidae
AGTA02016585	Aqp 11b	Zebra mbuna	*Maylandia zebra*	Acanthopterygii	Perciformes	Cichlidae
AFNX01007330_1	Aqp 11b	Red mwanza	*Pundamilia nyererei*	Acanthopterygii	Perciformes	Cichlidae
AFNY01013174	Aqp 11b	Lyretail cichlid	*Neolamprologus brichardi*	Acanthopterygii	Perciformes	Cichlidae
AFNZ01003545	Aqp 11b	Burton's mouthbrooder	*Haplochromis burtoni*	Acanthopterygii	Perciformes	Cichlidae
ENSONIP00000001816/AERX01020914/AERX01020915/XR_269848	Aqp 11b	Nile tilapia	*Oreochromis niloticus*	Acanthopterygii	Perciformes	Cichlidae
BADN01123543/BADN01119082	Aqp 11b	Pacific bluefin tuna	*Thunnus orientalis*	Acanthopterygii	Perciformes	Scombridae
AWGY01083649/AWGY01116558	Aqp 11b	Sablefish	*Anoplopoma fimbria*	Acanthopterygii	Scorpaeniformes	Anoplopomatidae
AUPQ01071328	Aqp 11b	Flag rockfish	*Sebastes rubrivinctus*	Acanthopterygii	Scorpaeniformes	Sebastidae
AUPR01089814	Aqp 11b	Tiger rockfish	*Sebastes nigrocinctus*	Acanthopterygii	Scorpaeniformes	Sebastidae
DK099434	Aqp 11b	Japanese medaka	*Oryzias latipes*	Acanthopterygii	Beloniformes	Adrianichthyidae
GAIB01048953	Aqp 11b	Turquoise killifish	*Nothobranchius furzeri*	Acanthopterygii	Cyprinodontiformes	Nothobranchiidae
FK039477	Aqp 11b	Platyfish hybrid	*X. maculatus x X. hellerii*	Acanthopterygii	Cyprinodontiformes	Poeciliidae
AYCK01004885	Aqp 11b	Amazon molly	*Poecilia formosa*	Acanthopterygii	Cyprinodontiformes	Poeciliidae
AGAJ01029174	Aqp 11b	Southern platyfish	*Xiphophorus maculatus*	Acanthopterygii	Cyprinodontiformes	Poeciliidae
ENSGMOP00000020707/CAEA01239425/CAEA01158306	Aqp 11b	Atlantic cod	*Gadus morhua*	Paracanthopterygii	Gadiformes	Gadidae
ACO09336	Aqp 11b	Rainbow smelt	*Osmerus mordax*	Protacanthopterygii	Osmeriformes	Osmeridae
CCAF010079483	Aqp 11b1	Rainbow trout	*Oncorhynchus mykiss*	Protacanthopterygii	Salmoniformes	Salmonidae
AGKD01193048	Aqp 11b1	Atlantic salmon	*Salmo salar*	Protacanthopterygii	Salmoniformes	Salmonidae
CCAF010068337	Aqp 11b2	Rainbow trout	*Oncorhynchus mykiss*	Protacanthopterygii	Salmoniformes	Salmonidae
AGKD01088157	Aqp 11b2	Atlantic salmon	*Salmo salar*	Protacanthopterygii	Salmoniformes	Salmonidae
DT287811	Aqp 11b	Fathead minnow	*Pimephales promelas*	Ostariophysi	Cypriniformes	Cyprinidae
GAHO01136304/GAHO01095742/GAHO01095336	Aqp 11b	Small gill opening goldenline barbel	*Sinocyclocheilus angustiporus*	Ostariophysi	Cypriniformes	Cyprinidae
GAHL01087546	Aqp 11b	Blind goldenline barbel	*Sinocyclocheilus anophthalmus*	Ostariophysi	Cypriniformes	Cyprinidae
BC095775/ENSDARP00000062519	Aqp 11b	Zebrafish	*Danio rerio*	Ostariophysi	Cypriniformes	Cyprinidae
APWO01110971/ENSAMXP00000014175	Aqp 11b	Mexican tetra	*Astyanax mexicanus*	Ostariophysi	Characiformes	Characidae
FD343108	Aqp 11b	Channel catfish	*Ictalurus punctatus*	Ostariophysi	Siluriformes	Ictaluridae
AHH39648	Aqp 11b	Brown bullhead	*Ameiurus nebulosus*	Ostariophysi	Siluriformes	Ictaluridae
AVPY01077179	Aqp 11b	Japanese eel	*Anguilla japonica*	Elopomorpha	Anguilliformes	Anguillidae
AHAT01003066/XP_006628169	Aqp 11-1	Spotted gar	*Lepisosteus oculatus*	Holostei	Semionotiformes	Lepisosteidae
AHAT01003066/XP_006628168	Aqp 11-2	Spotted gar	*Lepisosteus oculatus*	Holostei	Semionotiformes	Lepisosteidae
AESE010020706/AESE012695651/AESE012564622	Aqp 11	Little skate	*Leucoraja erinacea*	Chondrichthyes	Rajiformes	Rajidae

AQP12

ENSP00000337144	AQP 12A	Human	*Homo sapiens*	Euarchontoglires	Primates	Hominidae
ENSP00000384894	AQP 12B	Human	*Homo sapiens*	Euarchontoglires	Primates	Hominidae
ENSGGOP00000019546	AQP 12A	Western lowland gorilla	*Gorilla gorilla gorilla*	Euarchontoglires	Primates	Hominidae
ENSGGOP00000022773	AQP 12B	Western lowland gorilla	*Gorilla gorilla gorilla*	Euarchontoglires	Primates	Hominidae
ENSPPYP00000014971	AQP 12A	Sumatran orangutan	*Pongo abelii*	Euarchontoglires	Primates	Hominidae
ENSNLEP00000016587	AQP 12	Northern white-cheeked gibbon	*Nomascus leucogenys*	Euarchontoglires	Primates	Hylobatidae
AEHK01046637	AQP 12	Rhesus macaque	*Macaca mulatta*	Euarchontoglires	Primates	Cercopithecidae
EHH55306	AQP 12	Crab-eating macaque	*Macaca fascicularis*	Euarchontoglires	Primates	Cercopithecidae
ENSP00000405899	AQP 12	Hamadryas baboon	*Papio hamadryas*	Euarchontoglires	Primates	Cercopithecidae
XP_003908224	AQP 12	Olive baboon	*Papio anubis*	Euarchontoglires	Primates	Cercopithecidae
ENSCJAP00000012100	AQP 12	White-tufted-ear marmoset	*Callithrix jacchus*	Euarchontoglires	Primates	Cebidae
XP_003936813	AQP 12	Bolivian squirrel monkey	*Saimiri boliviensis boliviensis*	Euarchontoglires	Primates	Cebidae
ABRT02338252	AQP 12	Philippine tarsier	*Tarsius syrichta*	Euarchontoglires	Primates	Tarsiidae
ENSOGAP00000012879/XP_003798328	AQP 12	Small-eared galago/Bushbaby	*Otolemur garnettii*	Euarchontoglires	Primates	Galagidae
ALAR01071477/ELW63513	AQP 12	Chinese tree shrew	*Tupaia chinensis*	Euarchontoglires	Scandentia	Tupaiidae
ENSMUSP00000060622	AQP 12	Mouse	*Mus musculus*	Euarchontoglires	Rodentia	Muridae
ENSRNOP00000005935	AQP 12	Norway rat	*Rattus norvegicus*	Euarchontoglires	Rodentia	Muridae
XP_003499323	AQP 12	Chinese hamster	*Cricetulus griseus*	Euarchontoglires	Rodentia	Cricetidae
ABRO01568331	AQP 12	Ord's kangaroo rat	*Dipodomys ordii*	Euarchontoglires	Rodentia	Heteromyidae
ENSCPOP00000005935	AQP 12	Domestic guinea pig	*Cavia porcellus*	Euarchontoglires	Rodentia	Caviidae
EHB16168	AQP 12	Naked mole-rat	*Heterocephalus glaber*	Euarchontoglires	Rodentia	Bathyergidae
AGTP01102324	AQP 12	Thirteen-lined ground squirrel	*Ictidomys tridecemlineatus*	Euarchontoglires	Rodentia	Sciuridae
ENSOPRP00000004455	AQP 12	American pika	*Ochotona princeps*	Euarchontoglires	Lagomorpha	Ochotonidae
AAGW02082056	AQP 12	Rabbit	*Oryctolagus cuniculus*	Euarchontoglires	Lagomorpha	Leporidae
ENSSARP00000004517	AQP 12	Common shrew	*Sorex araneus*	Laurasiatheria	Insectivora	Soricidae

XP_004695608	AQP 12	Star-nosed mole	*Condylura cristata*	Laurasiatheria	Insectivora	Talpidae
ALEH01019818	AQP 12	Big brown bat	*Eptesicus fuscus*	Laurasiatheria	Chiroptera	Vespertilionidae
ENSMLUP00000003942	AQP 12	Little brown bat/Microbat	*Myotis lucifugus*	Laurasiatheria	Chiroptera	Vespertilionidae
ELK32658	AQP 12	David´s myotis	*Myotis davidii*	Laurasiatheria	Chiroptera	Vespertilionidae
ACIV010275453/ACIV010275458	AQP 12	Sheep	*Ovis aries*	Laurasiatheria	Ruminantia	Bovidae
ENSBTAP00000004395/XP_005205120	AQP 12	Cow	*Bos taurus*	Laurasiatheria	Ruminantia	Bovidae
ELR52245	AQP 12	Yak	*Bos grunniens mutus*	Laurasiatheria	Ruminantia	Bovidae
AGFL01032459	AQP 12	Zebu	*Bos indicus*	Laurasiatheria	Ruminantia	Bovidae
ATDI01036423	AQP 12	Minke whale	*Balaenoptera acutorostrata*	Laurasiatheria	Cetacea	Balaenopteridae
AOCR01104471	AQP 12	Pig	*Sus scrofa*	Laurasiatheria	Suina	Suidae
XP_006209258	AQP 12	Alpaca	*Vicugna pacos*	Laurasiatheria	Tylopoda	Camelidae
XP_006175578	AQP 12	Wild Bactrian camel	*Camelus ferus*	Laurasiatheria	Tylopoda	Camelidae
ENSAMEP00000017752	AQP 12	Giant panda	*Ailuropoda melanoleuca*	Laurasiatheria	Carnivora	Ursidae
ENSCAFP00000032136/AACN011020224/ AAEX03014544	AQP 12	Dog	*Canis lupus familiaris*	Laurasiatheria	Carnivora	Canidae
ENSMPUP00000000138	AQP 12	Domestic ferret	*Mustela putorius furo*	Laurasiatheria	Carnivora	Mustelidae
ENSFCAP00000009684/XP_003991374	AQP 12	Domestic cat	*Felis catus*	Laurasiatheria	Carnivora	Felidae
ENSECAP00000011522	AQP 12	Horse	*Equus caballus*	Laurasiatheria	Perissodactyla	Equidae
XP_004427863	AQP 12	Southern white rhinocerus	*Ceratotherium simum simum*	Laurasiatheria	Perissodactyla	Rhinocerotidae
ABRQ01627555	AQP 12	Cape rock hyrax	*Procavia capensis*	Afrotheria	Hyracoidea	Procaviidae
ENSLAFP00000001690	AQP 12	African savanna elephant	*Loxodonta africana*	Afrotheria	Proboscidea	Elephantidae
ENSETEP00000015332	AQP 12	Lesser hedgehog tenrec	*Echinops telfairi*	Afrotheria	Afrosoricida	Tenrecidae
AHIN01119182	AQP 12	Florida manatee	*Trichechus manatus latirostris*	Afrotheria	Sirenia	Trichechidae
ALYB01014398	AQP 12	Ardvaark	*Orycteropus afer afer*	Afrotheria	Tubulidentata	Orycteropodidae
AAGV03163531/AAGV03163532/AAGV03310742	AQP 12	Nine-banded armadillo	*Dasypus novemcinctus*	Xenarthra	Cingulata	Dasypodidae
ENSMEUP00000006074	AQP 12	Tammar wallaby	*Macropus eugenii*	Metatheria	Diprotodontia	Macropodidae
ENSSHAP00000013776	AQP 12	Tasmanian devil	*Sarcophilus harrisii*	Metatheria	Dasyuromorphia	Dasyuridae
ENSMODP00000023015	AQP 12	Gray short-tailed opossum	*Monodelphis domestica*	Metatheria	Didelphimorphia	Didelphidae
XP_001507166	AQP 12	Platypus	*Ornithorhynchus anatinus*	Prototheria	Monotremata	Ornithorhynchidae
AGAI01047968	AQP 12	Budgerigar	*Melopsittacus undulatus*	Aves	Psittaciformes	Psittacidae
AOCU01008568	AQP 12	Puerto Rican parrot	*Amazona vittata*	Aves	Psittaciformes	Psittacidae
AOUJ01133298/AMXX01085365	AQP 12	Scarlet macaw	*Ara macao*	Aves	Psittaciformes	Psittacidae
CAVT010015752	AQP 12	Common canary	*Serinus canaria*	Aves	Passeriformes	Fringillidae
AKZB01087418	AQP 12	Medium ground finch	*Geospiza fortis*	Aves	Passeriformes	Fringillidae
XP_005489598	AQP 12	White-throated sparrow	*Zonotrichia albicollis*	Aves	Passeriformes	Fringillidae
ENSTGUP00000005991	AQP 12	Zebra finch	*Taeniopygia guttata*	Aves	Passeriformes	Estrildidae
XP_005050802	AQP 12	Tibetan ground-tit	*Pseudopodoces humilis*	Aves	Passeriformes	Paridae
AGTO01011305	AQP 12	Collared flycatcher	*Ficedula albicollis*	Aves	Passeriformes	Muscicapidae
XP_005241620	AQP 12	Peregrin falcon	*Falco peregrinus*	Aves	Falconiformes	Falconidae
XP_005437199	AQP 12	Saker falcon	*Falco cherrug*	Aves	Falconiformes	Falconidae
XP_005501622	AQP 12	Rock pigeon	*Columba livia*	Aves	Columbiformes	Columbidae
ENSAPLP00000015645	AQP 12	Mallard	*Anas platyrhynchos*	Aves	Anseriformes	Anatidae
ENSMGAP00000002820	AQP 12	Turkey	*Meleagris gallopavo*	Aves	Galliformes	Phasianidae
ENSGALP00000010373	AQP 12	Chicken	*Gallus gallus*	Aves	Galliformes	Phasianidae
AKHW01049300	AQP 12	American alligator	*Alligator mississippiensis*	Archosauria	Crocodylia	Alligatoridae
AVPB01013368	AQP 12	Chinese alligator	*Alligator sinensis*	Archosauria	Crocodylia	Alligatoridae
ENSPSIP00000008491	AQP 12	Chinese softshell turtle	*Pelodiscus sinensis*	Sauropsida	Testudines	Trionychidae
EMP38415	AQP 12	Green seaturtle	*Chelonia mydas*	Sauropsida	Testudines	Cheloniidae
AHGY01268029/AHGY01268030	AQP 12	Western painted turtle	*Chrysemys picta bellii*	Sauropsida	Testudines	Emydidae
AZIM01003495	AQP 12	King cobra	*Ophiophagus hannah*	Lepidosauria	Squamata	Elapidae
AEQU02097685	AQP 12	Burmese python	*Python molurus bivittatus*	Lepidosauria	Squamata	Pythonidae
ENSACAP00000015972	AQP 12	Green anole	*Anolis carolinensis*	Lepidosauria	Squamata	Iguanidae
AAH82904	AQP 12	African clawed frog	*Xenopus laevis*	Amphibia	Anura	Pipidae
ENSXETP00000059277	AQP 12	Western clawed frog	*Xenopus (Silurana) tropicalis*	Amphibia	Anura	Pipidae
GAQK01013142	AQP 12	Chinese salamander	*Hynobius chinensis*	Amphibia	Caudata	Hynobiidae
BAHO01076624/AFYH01210544	Aqp 12	Coelacanth	*Latimeria chalumnae*	Actinistia	Coelacanthiformes	Coelacanthidae
GAPS01018381	Aqp 12	Menado coelacanth	*Latimeria menadoensis*	Actinistia	Coelacanthiformes	Coelacanthidae
ENSTRUP00000041552	Aqp 12	Torafugu	*Takifugu rubripes*	Acanthopterygii	Tetraodontiformes	Tetraodontidae
AOOT01027882	Aqp 12	Sansaifugu	*Takifugu flavidus*	Acanthopterygii	Tetraodontiformes	Tetraodontidae
BAL44702	Aqp 12	Mefugu	*Takifugu obscurus*	Acanthopterygii	Tetraodontiformes	Tetraodontidae
ENSTNIP00000020773	Aqp 12	Green-spotted pufferfish	*Tetraodon nigroviridis*	Acanthopterygii	Tetraodontiformes	Tetraodontidae
JU383146	Aqp 12	Turbot	*Scophthalmus maximus*	Acanthopterygii	Pleuronectiformes	Scophthalmidae
GAAQ01001383	Aqp 12	Dover sole	*Solea solea*	Acanthopterygii	Pleuronectiformes	Soleidae
AGRG01015271	Aqp 12	Tongue sole	*Cynoglossus semilaevis*	Acanthopterygii	Pleuronectiformes	Cynoglossidae
FM150229	Aqp 12	Gilthead seabream	*Sparus aurata*	Acanthopterygii	Perciformes	Sparidae
GW670618	Aqp 12	Mi-iuy croaker	*Miichthys miiuy*	Acanthopterygii	Perciformes	Sciaenidae
AGTA02025659	Aqp 12	Zebra mbuna	*Maylandia zebra*	Acanthopterygii	Perciformes	Cichlidae
AFNX01016726	Aqp 12	Red mwanza	*Pundamilia nyererei*	Acanthopterygii	Perciformes	Cichlidae
AFNY01023777	Aqp 12	Lyretail cichlid	*Neolamprologus brichardi*	Acanthopterygii	Perciformes	Cichlidae
AFNZ01010976	Aqp 12	Burton's mouthbrooder	*Haplochromis burtoni*	Acanthopterygii	Perciformes	Cichlidae
ENSONIP00000003560	Aqp 12	Nile tilapia	*Oreochromis niloticus*	Acanthopterygii	Perciformes	Cichlidae
BADN01023638	Aqp 12	Pacific bluefin tuna	*Thunnus orientalis*	Acanthopterygii	Perciformes	Scombridae
GW344888	Aqp 12	Antarctic spiny plunderfish	*Harpagifer antarcticus*	Acanthopterygii	Perciformes	Harpagiferidae
AWGY01139415/AWGY01139417/AWGY01148047/ GAJJ01023328/GAJJ01023327	Aqp 12	Sablefish	*Anoplopoma fimbria*	Acanthopterygii	Scorpaeniformes	Anoplopomatidae
AUPQ01029045	Aqp 12	Flag rockfish	*Sebastes rubrivinctus*	Acanthopterygii	Scorpaeniformes	Sebastidae
AUPR01054694	Aqp 12	Tiger rockfish	*Sebastes nigrocinctus*	Acanthopterygii	Scorpaeniformes	Sebastidae
ENSGACP00000014661	Aqp 12	Three-spined stickleback	*Gasterosteus aculeatus*	Acanthopterygii	Gasterosteiformes	Gasterosteidae
ENSORLP00000001949	Aqp 12	Japanese medaka	*Oryzias latipes*	Acanthopterygii	Beloniformes	Adrianichthyidae
GAIB01022997	Aqp 12	Turquoise killifish	*Nothobranchius furzeri*	Acanthopterygii	Cyprinodontiformes	Nothobranchiidae
CN980894	Aqp 12	Common mummichog	*Fundulus heteroclitus*	Acanthopterygii	Cyprinodontiformes	Fundulidae
ES376331	Aqp 12	Guppy	*Poecilia reticulata*	Acanthopterygii	Cyprinodontiformes	Poeciliidae
AYCK01004047	Aqp 12	Amazon molly	*Poecilia formosa*	Acanthopterygii	Cyprinodontiformes	Poeciliidae
ENSXMAP00000011346	Aqp 12	Southern platyfish	*Xiphophorus maculatus*	Acanthopterygii	Cyprinodontiformes	Poeciliidae
ENSGMOP00000020613	Aqp 12	Atlantic cod	*Gadus morhua*	Paracanthopterygii	Gadiformes	Gadidae
CCAF010102094	Aqp 12-1	Rainbow trout	*Oncorhynchus mykiss*	Protacanthopterygii	Salmoniformes	Salmonidae
AGKD01402623	Aqp 12-1	Atlantic salmon	*Salmo salar*	Protacanthopterygii	Salmoniformes	Salmonidae
CCAF010023537	Aqp 12-2	Rainbow trout	*Oncorhynchus mykiss*	Protacanthopterygii	Salmoniformes	Salmonidae
AGKD01127630	Aqp 12-2	Atlantic salmon	*Salmo salar*	Protacanthopterygii	Salmoniformes	Salmonidae
EE396542/EE393754	Aqp 12	Rare gudgeon	*Gobiocypris rarus*	Ostariophysi	Cypriniformes	Cyprinidae
CA968566	Aqp 12	Common carp	*Cyprinus carpio*	Ostariophysi	Cypriniformes	Cyprinidae
GAHO01106926/GAHO01011211/GAHO01072357/G AHO01107961/GAHO01134744	Aqp 12	Small gill opening goldenline barbel	*Sinocyclocheilus angustiporus*	Ostariophysi	Cypriniformes	Cyprinidae
DT137447/GH711388	Aqp 12	Fathead minnow	*Pimephales promelas*	Ostariophysi	Cypriniformes	Cyprinidae
BC095564/ENSDARP00000063532	Aqp 12	Zebrafish	*Danio rerio*	Ostariophysi	Cypriniformes	Cyprinidae
APWO01096620/ENSAMXP00000015796	Aqp 12	Mexican tetra	*Astyanax mexicanus*	Ostariophysi	Characiformes	Characidae
GAGX01084582/GAGX01029051/GAGX01074097	Aqp 12	Brown bullhead	*Ameiurus nebulosus*	Ostariophysi	Siluriformes	Ictaluridae
CK408292	Aqp 12	Blue catfish	*Ictalurus furcatus*	Ostariophysi	Siluriformes	Ictaluridae
FD343108	Aqp 12	Channel catfish	*Ictalurus punctatus*	Ostariophysi	Siluriformes	Ictaluridae
AVPY01366016	Aqp 12	Japanese eel	*Anguilla japonica*	Elopomorpha	Anguilliformes	Anguillidae

AHAT01015202	Aqp 12	Spotted gar	*Lepisosteus oculatus*	Holostei	Semionotiformes	Lepisosteidae
AESE011498082/AESE010748237/AESE011637899	Aqp 12	Little skate	*Leucoraja erinacea*	Chondrichthyes	Rajiformes	Rajidae
AAVX02000347	Aqp 12	Ghost shark	*Callorhinchus milii*	Chondrichthyes	Chimaeriformes	Callorhinchidae
APJL01034632/APJL01136199/APJL01075977	Aqp 12L	Arctic lamprey	*Lethenteron camtschaticum*	Hyperoartia	Petromyzontiformes	Petromyzontidae
XP_002592039/ABEP02030099/JT884843/ JT866466	Aqp 12L1	Florida lancelet	*Branchiostoma floridae*	Cephalochordata	Amphioxiformes	Branchiostomidae
XP_002592040/ABEP02030099/JT884843	Aqp 12L2	Florida lancelet	*Branchiostoma floridae*	Cephalochordata	Amphioxiformes	Branchiostomidae
XP_002739302	Aqp 12L1	Acorn worm	*Saccoglossus kowalevskii*	Hemichordata	Enteropneusta	Harrimaniidae
XP_002741885	Aqp 12L2	Acorn worm	*Saccoglossus kowalevskii*	Hemichordata	Enteropneusta	Harrimaniidae
XP_780933/SPU_005585-tr	Aqp 12L1	Purple sea urchin	*Strongylocentrotus purpuratus*	Echinodermata	Echinozoa	Strongylocentrotidae
GAPB01023608	Aqp 12L1	Kina	*Evechinus chloroticus*	Echinodermata	Echinozoa	Echinometridae
AGCV01082820	Aqp 12L1	Green sea urchin	*Lytechinus variegatus*	Echinodermata	Echinozoa	Toxopneustidae
AKZP01011511	Aqp 12L1	Bat star	*Patiria miniata*	Echinodermata	Asterozoa	Asterinidae
AM198294/AM545885	Aqp 12L2	Common sea urchin	*Paracentrotus lividus*	Echinodermata	Echinozoa	Echinidae
JT108126/SPU_005947-tr	Aqp 12L2	Purple sea urchin	*Strongylocentrotus purpuratus*	Echinodermata	Echinozoa	Strongylocentrotidae
GAPB01014568	Aqp 12L2	Kina	*Evechinus chloroticus*	Echinodermata	Echinozoa	Echinometridae
AGCV01019958	Aqp 12L2	Green sea urchin	*Lytechinus variegatus*	Echinodermata	Echinozoa	Toxopneustidae
AKZP01028600	Aqp 12L2	Bat star	*Patiria miniata*	Echinodermata	Asterozoa	Asterinidae

Protostomia unorthodox aquaporins

Arthropoda unorthodox aquaporins

FBpp0086879	Aqp 12L	Fruit fly	*Drosophila melanogaster*	Hexapoda	Diptera	Drosophilidae
FBpp0201777	Aqp 12L	Fruit fly	*Drosophila sechellia*	Hexapoda	Diptera	Drosophilidae
FBpp0258396	Aqp 12L	Fruit fly	*Drosophila yakuba*	Hexapoda	Diptera	Drosophilidae
FBpp0141060	Aqp 12L	Fruit fly	*Drosophila erecta*	Hexapoda	Diptera	Drosophilidae
FBpp0224314	Aqp 12L	Fruit fly	*Drosophila simulans*	Hexapoda	Diptera	Drosophilidae
FBpp0116155	Aqp 12L	Fruit fly	*Drosophila ananassae*	Hexapoda	Diptera	Drosophilidae
FBpp0276666	Aqp 12L	Fruit fly	*Drosophila pseudoobscura*	Hexapoda	Diptera	Drosophilidae
FBpp0235888	Aqp 12L	Fruit fly	*Drosophila virilis*	Hexapoda	Diptera	Drosophilidae
FBpp0167611	Aqp 12L	Fruit fly	*Drosophila mojavensis*	Hexapoda	Diptera	Drosophilidae
FBpp0158826	Aqp 12L	Fruit fly	*Drosophila grimshawi*	Hexapoda	Diptera	Drosophilidae
FBpp0247034	Aqp 12L	Fruit fly	*Drosophila willistoni*	Hexapoda	Diptera	Drosophilidae
GAKB01003759	Aqp 12L	Olive fruit fly	*Bactrocera oleae*	Hexapoda	Diptera	Tephritidae
XP_004529905	Aqp 12L	Mediterranean fruit fly	*Ceratitis capitata*	Hexapoda	Diptera	Tephritidae
CAQQ02174372/CAQQ02174371/CAQQ02389937	Aqp 12L	Scuttle fly	*Megaselia scalaris*	Hexapoda	Diptera	Phoridae
EZ602043	Aqp 12L	Flesh fly	*Sarcophaga crassipalpis*	Hexapoda	Diptera	Sarcophagidae
FD463879	Aqp 12L	Horn fly	*Haematobia irritans irritans*	Hexapoda	Diptera	Muscidae
AQPM01092757	Aqp 12L	House fly	*Haematobia irritans irritans*	Hexapoda	Diptera	Muscidae
AFP49900	Aqp 12L	Tsetse fly	*Glossina morsitans morsitans*	Hexapoda	Diptera	Glossinidae
AAEL014255/XP_001648046	Aqp 12L	Yellow fever mosquito	*Aedes aegypti*	Hexapoda	Diptera	Culicidae
CPIJ004456	Aqp 12L	Southern house mosquito	*Culex quinquefasciatus*	Hexapoda	Diptera	Culicidae
AGAP010878	Aqp 12L	African malaria mosquito	*Anopheles gambiae*	Hexapoda	Diptera	Culicidae
EZ978777	Aqp 12L	African malaria mosquito	*Anopheles funestus*	Hexapoda	Diptera	Culicidae
EFR26850	Aqp 12L	American malaria mosquito	*Anopheles darlingi*	Hexapoda	Diptera	Culicidae
GAFE01006687	Aqp 12L	Indonesian malaria mosquito	*Anopheles sinensis*	Hexapoda	Diptera	Culicidae
GAAK01006945	Aqp 12L	Antarctic flightless midge	*Belgica antarctica*	Hexapoda	Diptera	Chironomidae
AEGA01009001/AEGA01031906	Aqp 12L	Hessian fly	*Mayetiola destructor*	Hexapoda	Diptera	Cecidomyiidae
JP551754	Aqp 12L	Sand fly	*Phlebotomus papatasi*	Hexapoda	Diptera	Psychodidae
AM094572/AM094570	Aqp 12L	Sand fly	*Lutzomyia longipalpis*	Hexapoda	Diptera	Psychodidae
HX266982	Aqp 12L	Domestic silkworm	*Bombyx mori*	Hexapoda	Lepidoptera	Bombycidae
CAEZ01010288	Aqp 12L	Postman butterfly	*Heliconius melpomene melpomene*	Hexapoda	Lepidoptera	Nymphalidae
EHJ69710	Aqp 12L	Monarch butterfly	*Danaus plexippus*	Hexapoda	Lepidoptera	Nymphalidae
GAFU01002466	Aqp 12L	Beet armyworm	*Spodoptera exigua*	Hexapoda	Lepidoptera	Noctuidae
GT199640/GT196938	Aqp 12L	Tobacco budworm	*Heliothis virescens*	Hexapoda	Lepidoptera	Noctuidae
HS099242	Aqp 12L	Maruca pod borer	*Maruca vitrata*	Hexapoda	Lepidoptera	Crambidae
JP615554/JP615553	Aqp 12L	Propertius duskywing	*Erynnis propertius*	Hexapoda	Lepidoptera	Hesperiidae
AIXA01009041	Aqp 12L	Tobacco hornworm	*Manduca sexta*	Hexapoda	Lepidoptera	Sphingidae
HS099242	Aqp 12L	Maruca pod borer	*Maruca vitrata*	Hexapoda	Lepidoptera	Crambidae
BAGR01006886/BAGR01078921/BAGR01078920	Aqp 12L	Diamondback Moth	*Plutella xylostella*	Hexapoda	Lepidoptera	Plutellidae
GAXW01087864	Aqp 12L	Antlion	*Euroleon nostras*	Hexapoda	Neoptera	Myrmeleontidae
GAVV01020125	Aqp 12L	Green lacewing	*Pseudomallada prasinus*	Hexapoda	Neoptera	Chrysopidae
XP_974208	Aqp 12L	Red flour Beetle	*Tribolium castaneum*	Hexapoda	Coleoptera	Tenebrionidae
AQHT01000891/AQHT01000892	Aqp 12L	Asian longhorned beetle	*Anoplophora glabripennis*	Hexapoda	Coleoptera	Cerambycidae
GAFI01011142	Aqp 12L	Southern pine beetle	*Dendroctonus frontalis*	Hexapoda	Coleoptera	Curculionidae
APGL01029398	Aqp 12L	Mountain pine beetle	*Dendroctonus ponderosae*	Hexapoda	Coleoptera	Curculionidae
JR469890	Aqp 12L	Red palm weevil	*Rhynchophorus ferrugineus*	Hexapoda	Coleoptera	Curculionidae
GAEO01001684	Aqp 12L	White pine weevil	*Pissodes strobi*	Hexapoda	Coleoptera	Curculionidae
AGRH01000202	Aqp 12L	Reticulated beetle	*Priacma serrata*	Hexapoda	Coleoptera	Cupedidae
XP_003393649	Aqp 12L	Buff-tailed bumblebee	*Bombus terrestris*	Hexapoda	Hymenoptera	Apidae
XP_003490105	Aqp 12L	Common eastern bumble bee	*Bombus impatiens*	Hexapoda	Hymenoptera	Apidae
XP_001119893	Aqp 12L	Honey bee	*Apis mellifera*	Hexapoda	Hymenoptera	Apidae
ANOB01024207	Aqp 12L	Halictid Bee	*Lasioglossum albipes*	Hexapoda	Hymenoptera	Halictidae
GAGH01010151	Aqp 12L	Mason bee	*Osmia cornuta*	Hexapoda	Hymenoptera	Megachilidae
AFJA01002269	Aqp 12L	Alfalfa leafcutting bee	*Megachile rotundata*	Hexapoda	Hymenoptera	Megachilidae
EFN85024	Aqp 12L	Jerdon's jumping ant	*Harpegnathos saltator*	Hexapoda	Hymenoptera	Formicidae
EFN70241	Aqp 12L	Florida carpenter ant	*Camponotus floridanus*	Hexapoda	Hymenoptera	Formicidae
ADTU01018182	Aqp 12L	Leafcutter ant	*Atta cephalotes*	Hexapoda	Hymenoptera	Formicidae
EGI57993	Aqp 12L	Panamanian leafcutter ant	*Acromyrmex echinatior*	Hexapoda	Hymenoptera	Formicidae
FQ836707/FQ840903	Aqp 12L	Parasitoid wasp	*Asobara tabida*	Hexapoda	Hymenoptera	Braconidae
AOFN01007317	Aqp 12L	Coleseed sawfly	*Athalia rosae*	Hexapoda	Hymenoptera	Tenthredinidae
PHUM127940	Aqp 12L	Human body louse	*Pediculus humanus corporis*	Hexapoda	Phthiraptera	Pediculidae
ACPB02038779/ACPB02002207	Aqp 12L	Assassin bug	*Rhodnius prolixus*	Hexapoda	Hemiptera	Reduviidae
KF048101	Aqp 12L	Lygus bug	*Lygus hesperus*	Hexapoda	Hemiptera	Miridae
EZ940769/HP803079	Aqp 12L	Sweet potato whitefly	*Bemisia tabaci*	Hexapoda	Hemiptera	Aleyrodidae
HS430594	Aqp 12L	Brown planthopper	*Nilaparvata lugens*	Hexapoda	Hemiptera	Delphacidae
GAGF01017370	Aqp 12L	Green lacewing	*Chrysopa pallens*	Hexapoda	Neoptera	Chrysopidae
FG130040	Aqp 12L	American cockroach	*Periplaneta americana*	Hexapoda	Blattodea	Blattidae
GBID01003279	Aqp 12L	German cockroach	*Blattella germanica*	Hexapoda	Blattodea	Ectobiidae
AUST01032598/AUST01032597	Aqp 12L	Nevada dampwood termite	*Zootermopsis nevadensis*	Hexapoda	Isoptera	Termopsidae
FX376324	Aqp 12L	Wood-eating higher termite	*Nasutitermes takasagoensis*	Hexapoda	Isoptera	Termitidae
GABA01001239	Aqp 12L	Zoraptid	*Zorotypus gurneyi*	Hexapoda	Zoraptera	Zorotypidae
GAWE01136841/GAWE01162972/GAWE01022485	Aqp 12L	Vietnamese walking stick insect	*Ramulus artemis*	Hexapoda	Phasmatodea	Phasmatidae
GAIZ01025748	Aqp 12L	Sand field cricket	*Gryllus firmus*	Hexapoda	Orthoptera	Gryllidae
EH632839	Aqp 12L	Hawaiian swordtail cricket	*Laupala kohalensis*	Hexapoda	Orthoptera	Gryllidae
CO854318/CO854607	Aqp 12L	Migratory locust	*Locusta migratoria*	Hexapoda	Orthoptera	Acrididae
GAEQ01010065	Aqp 12L	Hagen´s bluet	*Enallagma hageni*	Hexapoda	Odonata	Coenagrionidae
APVN01144267	Aqp 12L	Scarce chaser	*Ladona fulva*	Hexapoda	Odonata	Libellulidae
AYNC01076740	Aqp 12L	Green drake	*Ephemera danica*	Hexapoda	Ephemeroptera	Ephemeridae
GAMM01011672	Aqp 12L	Springtail	*Orchesella cincta*	Hexapoda	Collembola	Entomobryidae

GAMN01009381	Aqp 12L	Springtail	*Folsomia candida*	Hexapoda	Collembola	Isotomidae
AFFK01022088	Aqp 12L	Coastal European centipede	*Strigamia maritima*	Chilopoda	Geophilomorpha	Linotaeniidae
JR223268	Aqp 12L	Black tiger shrimp	*Penaeus monodon*	Malacostraca	Decapoda	Penaeidae
JP419814/JP406388	Aqp 12L	Pacific white shrimp	*Litopenaeus vannamei*	Malacostraca	Decapoda	Penaeidae
EX568605	Aqp 12L	American lobster	*Homarus americanus*	Malacostraca	Decapoda	Nephropidae
FE773872	Aqp 12L	Flat porcelain crab	*Petrolisthes cinctipes*	Malacostraca	Decapoda	Porcellanidae
DN634964	Aqp 12L	Green shore Crab	*Carcinus maenas*	Malacostraca	Decapoda	Carcinidae
GAKD01002926	Aqp 12L	Amphipod	*Melita plumulosa*	Malacostraca	Amphipoda	Melitidae
ES505590	Aqp 12L	Brine Shrimp	*Artemia franciscana*	Branchiopoda	Anostraca	Artemiidae
EFX67799	Aqp 12L	Common water flea	*Daphnia pulex*	Branchiopoda	Diplostraca	Daphniidae
ACO11400	Aqp 12L	Caligid copepod	*Caligus rogercresseyi*	Maxillopoda	Siphonostomatoida	Caligidae
ACO14967	Aqp 12L	Caligid copepod	*Caligus clemensi*	Maxillopoda	Siphonostomatoida	Caligidae
GO412603	Aqp 12L	Cod worm	*Lernaeocera branchialis*	Maxillopoda	Siphonostomatoida	Pennellidae
EH666294	Aqp 12L	Calanus	*Calanus finmarchicus*	Maxillopoda	Calanoida	Calanidae
XP_002416580	Aqp 12L	Blacklegged tick	*Ixodes scapularis*	Acari	Ixodida	Ixodidae
ADDG01005800	Aqp 12L	Honeybee mite	*Varroa destructor*	Acari	Mesostigmata	Varroidae
XP_003740565	Aqp 12L	Western predatory mite	*Metaseiulus occidentalis*	Acari	Mesostigmata	Phytoseiidae
JR698214	Aqp 12L	Two-spotted spider mite	*Tetranychus urticae*	Acari	Acariformes	Tetranychidae
GANL01000406	Aqp 12L	Black widow	*Latrodectus tredecimguttatus*	Arachnida	Araneae	Theridiidae
GBCS01016346	Aqp 12L	Western black widow	*Latrodectus hesperus*	Arachnida	Araneae	Theridiidae
AOMJ01188201/AOMJ01188200/AOMJ01188199	Aqp 12L	Common house spider	*Parasteatoda tepidariorum*	Arachnida	Araneae	Theridiidae
JT033535	Aqp 12L	Subsocial spider	*Stegodyphus lineatus*	Arachnida	Araneae	Theridiidae
EY189672	Aqp 12L	Spider	*Loxosceles laeta*	Arachnida	Araneae	Sicariidae
GAZS01072938	Aqp 12L	Brazilian giant whiteknee tarantula	*Acanthoscurria geniculata*	Arachnida	Araneae	Theraphosidae
AYEL01083560	Aqp 12L	Chinese scorpion	*Mesobuthus martensii*	Arachnida	Scorpiones	Buthidae
AXZI01164565/AXZI01164567	Aqp 12L	Baja California bark scorpion	*Centruroides exilicauda*	Arachnida	Scorpiones	Buthidae
AEP14565	Aqp 12L(11)	Water bear	*Milnesium tardigradum*	Tardigrada	Apochela	Milnesiidae

Other Protostomia unorthodox aquaporins

AFFK01022088	Aqp 12L	Coastal European centipede	*Strigamia maritima*	Myriapoda	Geophilomorpha	Linotaeniidae
JR223268	Aqp 12L	Black tiger shrimp	*Penaeus monodon*	Malacostraca	Decapoda	Penaeidae
JP419814/JP406388	Aqp 12L	Pacific white shrimp	*Litopenaeus vannamei*	Malacostraca	Decapoda	Penaeidae
EX568605	Aqp 12L	American lobster	*Homarus americanus*	Malacostraca	Decapoda	Nephropidae
FE773872	Aqp 12L	Flat porcelain crab	*Petrolisthes cinctipes*	Malacostraca	Decapoda	Porcellanidae
DN634964	Aqp 12L	Green shore Crab	*Carcinus maenas*	Malacostraca	Decapoda	Carcinidae
GAKD01002926	Aqp 12L	Amphipod	*Melita plumulosa*	Malacostraca	Amphipoda	Melitidae
ES505590	Aqp 12L	Brine Shrimp	*Artemia franciscana*	Branchiopoda	Anostraca	Artemiidae
EFX67799	Aqp 12L	Common water flea	*Daphnia pulex*	Branchiopoda	Diplostraca	Daphniidae
ACO11400	Aqp 12L	Caligid copepod	*Caligus rogercresseyi*	Maxillopoda	Siphonostomatoida	Caligidae
ACO14967	Aqp 12L	Caligid copepod	*Caligus clemensi*	Maxillopoda	Siphonostomatoida	Caligidae
GO412603	Aqp 12L	Cod worm	*Lernaeocera branchialis*	Maxillopoda	Siphonostomatoida	Pennellidae
EH666294	Aqp 12L	Calanus	*Calanus finmarchicus*	Maxillopoda	Calanoida	Calanidae
XP_002416580	Aqp 12L	Blacklegged tick	*Ixodes scapularis*	Acari	Ixodida	Ixodidae
ADDG01005800	Aqp 12L	Honeybee mite	*Varroa destructor*	Acari	Mesostigmata	Varroidae
XP_003740565	Aqp 12L	Western predatory mite	*Metaseiulus occidentalis*	Acari	Mesostigmata	Phytoseiidae
JR698214	Aqp 12L	Two-spotted spider mite	*Tetranychus urticae*	Acari	Acariformes	Tetranychidae
GANL01000406	Aqp 12L	Black widow	*Latrodectus tredecimguttatus*	Arachnida	Araneae	Theridiidae
GBCS01016346	Aqp 12L	Western black widow	*Latrodectus hesperus*	Arachnida	Araneae	Theridiidae
AOMJ01188201/AOMJ01188200/AOMJ01188199	Aqp 12L	Common house spider	*Parasteatoda tepidariorum*	Arachnida	Araneae	Theridiidae
JT033535	Aqp 12L	Subsocial spider	*Stegodyphus lineatus*	Arachnida	Araneae	Theridiidae
EY189672	Aqp 12L	Spider	*Loxosceles laeta*	Arachnida	Araneae	Sicariidae
GAZS01072938	Aqp 12L	Brazilian giant whiteknee tarantula	*Acanthoscurria geniculata*	Arachnida	Araneae	Theraphosidae
AYEL01083560	Aqp 12L	Chinese scorpion	*Mesobuthus martensii*	Arachnida	Scorpiones	Buthidae
AXZI01164565/AXZI01164567	Aqp 12L	Baja California bark scorpion	*Centruroides exilicauda*	Arachnida	Scorpiones	Buthidae
AEP14565	Aqp 12L	Water bear	*Milnesium tardigradum*	Tardigrada	Apochela	Milnesiidae
LotgiP205153	Aqp 12L	Owl limpet	*Lottia gigantea*	Mollusca		Lottiidae
GAGS01057511	Aqp 12L	Freshwater snail	*Bithynia siamensis goniomphalos*	Mollusca		Bithyniidae
XP_005090480	Aqp 12L	California sea hare	*Aplysia californica*	Mollusca		Aplysiidae
GADG01003539	Aqp 12L	Sea scallop	*Placopecten magellanicus*	Mollusca	Pectinoida	Pectinidae
EKC30861	Aqp 12L1	Pacific oyster	*Crassostrea gigas*	Mollusca	Ostreoida	Ostreidae
EKC22781	Aqp 12L2	Pacific oyster	*Crassostrea gigas*	Mollusca	Ostreoida	Ostreidae
JI267975	Aqp 12L	Asiatic hard clam	*Meretrix meretrix*	Mollusca	Veneroida	Veneridae
FO193330	Aqp 12L	Common cuttlefish	*Sepia officinalis*	Mollusca	Sepiida	Sepiidae
CaptePP65598	Aqp 12L	Segmented worm	*Capitella teleta*	Annelida	Capitellida	Capitellidae
K07A1.16a	Aqp 12L	Roundworm	*Caenorhabditis elegans*	Nematoda	Rhabditida	Rhabditidae
ZK1321.3	Aqp 12L	Roundworm	*Caenorhabditis elegans*	Nematoda	Rhabditida	Rhabditidae
ZK525.2	Aqp 12L	Roundworm	*Caenorhabditis elegans*	Nematoda	Rhabditida	Rhabditidae
Bm7672b	Aqp 12L	Roundworm	*Brugia malayi*	Nematoda	Spirurida	Onchocercidae
XP_001900613	Aqp 12L	Roundworm	*Brugia malayi*	Nematoda	Spirurida	Onchocercidae
EJW87628	Aqp 12L	Roundworm	*Wuchereria bancrofti*	Nematoda	Spirurida	Onchocercidae
XP_003142473	Aqp 12L	Eye worm	*Loa loa*	Nematoda	Spirurida	Onchocercidae
PPA02276	Aqp 12L	Roundworm	*Pristionchus pacificus*	Nematoda	Diplogasterida	Neodiplogasteridae

Radiata unorthodox aquaporins

BACK01037703	Aqp 12L	Stony coral	*Acropora digitifera*	Cnidaria	Scleractinia	Acroporidae
JT003875	Aqp 12L	Stony coral	*Acropora millepora*	Cnidaria	Scleractinia	Acroporidae
GASU01017125	Aqp 12L	Stony coral	*Acropora cervicornis*	Cnidaria	Scleractinia	Acroporidae
DR983976/DR983975	Aqp 12L	Stony coral	*Acropora palmata*	Cnidaria	Scleractinia	Acroporidae
FX461932	Aqp 12L	Stony coral	*Porites australiensis*	Cnidaria	Scleractinia	Poritidae
v1g213742/XP_001647522	Aqp 12L	Starlet sea anemone	*Nematostella vectensis*	Cnidaria	Actiniaria	Edwardsiidae
ACZU01104846	Aqp 12L	Freshwater anemone	*Hydra vulgaris*	Cnidaria	Hydroida	Hydridae
FP957939	Aqp 12L	Thecate hydroid	*Clytia hemisphaerica*	Cnidaria	Hydroida	Campanulariidae

Archaea AqpM

AB055880	Aqp M	Archaea	*Methanothermobacter thermautotrophicus*	Euryarchaeota	Methanobacteriales	Methanobacteriaceae
NP_988083	Aqp M	Archaea	*Methanococcus maripaludis*	Euryarchaeota	Methanococcales	Methanococcaceae
YP_003435795	Aqp M	Archaea	*Ferroglobus placidus*	Euryarchaeota	Archaeoglobales	Archaeoglobaceae
YP_843562	Aqp M	Archaea	*Methanosaeta thermophila*	Euryarchaeota	Methanosarcinales	Methanosaetaceae
YP_305556	Aqp M	Archaea	*Methanosarcina barkeri*	Euryarchaeota	Methanosarcinales	Methanosarcinaceae

Supplementary Table 2. Aromatic-arginine constriction residues in vertebrate and hexapod aquaporins

	AQP8				AQP4			
Vertebrata	TMD2	TMD5	LE1	LE2	TMD2	TMD5	LE1	LE2
**Mammalia**	H	I	C	R	F	H	A	R
**Sauropsida**	H	I,V	C	R	F	H	A	R
**Amphibia**	H	I	C	R	F	H	A	R
**Actinistia**	H	I	C	R	F	H	A	R
**Teleostei**	H	I,V	C	R	F	H	A	R
**Holostei**	H	I	C	R	F	H	A	R
**Chondrichthyes**					F	H	A	R
**Hyperoartia**	H	I	C	R	F	H	A	R
**Hyperotreti**					F	H	S	R

	Drip				Prip				Eglp				Glp			
Hexapoda	TMD2	TMD5	LE1	LE2	TMD2	TMD5	LE1	LE2	TMD2	TMD5	LE1	LE2	TMD2	TMD5	LE1	LE2
**Diptera**	F	H	A	R	F	H	S,A,G	R	F	A,S,V,I,G	A,G,S	R				
**Lepidoptera**	F	H	S	R	F	H	S	R	F	A,S,V,I	A,G	R				
**Coleoptera**	F	H	S	R	F	H	S,A	R	F	A,S,V	A,G	R				
**Neuroptera**	F	H	S	R	F	H	S	R	F	A,S,C	C	R				
**Hymenoptera**	F	H	S	R	F	H	A,T	R	F	A,S,C	C	R				
**Psocodea**	F	H	S,A	R	F	H	S,A	R	F	A,S,V,G,D	A,G,C	R	Y,F	G,A	F,Y	R
**Hemiptera**	F	H	S,A	R	F	H	S,A	R	F,S	A,S,L	A,S,G,C,N	R				
**Isoptera**	F	H	A	R	F	H	S	R	F	A	G	R				
**Blattodea**	F	H	S,A	R	F	H	S,A	R	F	A	S	R				
**Mantodea**	F	H	A	R	F	H	S	R	F	A	A	R				
**Phasmatodea**	F	H	S	R	F	H	S	R	F	A	A	R				
**Embioptera**	F	H	S	R	F	H	S	R	F	A	A	R	-	G	Y	R
**Mantophasmatodea**	F	H	A	R	F	H	S	R	F	A	A,G	R	Y	G	Y	R
**Orthoptera**	F	H	S,A	R	F	H	S,A	R	F	A,S	A,G	R	Y	G,I	Y	R
**Plecoptera**	F	H	S,A	R	F	H	S,A	R	F	A,S	A	R	Y	G	Y	R
**Dermaptera**	F	H	S	R	F	H	A	R					Y	-	Y	-
**Zoraptera**	F	H	S	R					F	S	A	R	Y	G	Y	R
**Ephemeroptera**	F	H	S	R	F	H	S	R	F	A,G,V	A,G	R	Y	G	Y	R
**Odonata**	F	H	S	R	F	H	S,A	R	F	A,G	A,P	R	W	G	Y	R
**Zygentoma**	F	H	S,A	R	F	H	S,A,G	R	F	S	A	R	Y	G	Y	R
**Archaeognatha**	F	H	S,A	R	F	H	S,A,G	R	F,A	A	A	R	Y	G	Y	R
**Diplura**	F	H	S	R	F,V	H	S,A,G	R					W	G	Y	R
**Collembola**	F	H	S,A,T	R	F	H	S	R	F,W	I,V	G,S	R	W,G,S,T,Q,I,V	G,A,I,N,S	Y,G,A,T	R
**Protura**					F	H	C	R	A,G	S,I	S	R	W	G	Y	R
**Crustacea**					F,V	H	S,A	R					Y,W,F	G,H,A,F,L	Y,A,T,C	R
**Myriapoda**					F,A	H	S,G	R					W	G	Y	R
**Chelicerata**					F	H	S	R					W,F,A,S	A,C,I,M	A,C,N,E	R
